# Supplementary material for: Tunable and nonlinearity-enhanced dispersive-plus-dissipative coupling in photon-pressure circuits
Source: Nat Commun. 2026 Mar 24;17:2789. doi: 10.1038/s41467-026-70459-3 (PMC13018200; doi:10.1038/s41467-026-70459-3)
Supplement: Supplementary file 1 — Supplementary Information [file 41467_2026_70459_MOESM1_ESM.pdf]

Supplementary Material for:

Tunable and nonlinearity-enhanced dispersive-plus-dissipative coupling in photon-pressure circuits

M. Kazouini, J. Peter, Z. E. Guo, B. Wilde, K. Uhl, D. Koelle, R. Kleiner, and D. Bothner

CONTENTS

List of most important parameters and variables 3

1. Supplementary Note 1: Measurement setup 4

2. Supplementary Note 2: The individual circuits without constrictions 6
   A. The high-frequency circuit without constrictions 6
   B. The low-frequency circuit without constrictions 6
   C. Circuit parameters 6

3. Supplementary Note 3: The individual circuits with constrictions 8
   A. Including the constrictions 8
   B. Describing the SQUID 8
   C. The total circuit including constrictions – Dispersive part 9
   D. The total circuit including constrictions – Dissipative part 11
   E. Single-photon coupling rates 12

4. Supplementary Note 4: Theoretical response of the individual circuits 14
   A. The low-frequency circuit – Complete equation of motion 14
   B. The low-frequency circuit – Single-tone response in the linear regime 14
   C. The low-frequency circuit – Single-tone response in the nonlinear regime 14
   D. The high-frequency circuit – Complete equation of motion 15
   E. The high-frequency circuit – Single-tone response in the linear regime 15
   F. The high-frequency circuit – The linearized two-tone response 15
   G. The pumped Kerr eigenmodes 17
   H. Intracircuit pump photon number 18

5. Supplementary Note 5: Theoretical model of the coupled circuits 19
   A. Hamiltonian and equations of motion 19
   B. Dropping constants, small shifts, the quadratic term and input noise 21
   C. Adding nonlinear damping 22
   D. Low-pump-power regime – Equations of motion 22
   E. Dynamical backaction in the low-pump-power regime 23
   F. Photon-pressure induced transparency in the low-pump-power regime 24
   G. High-pump-power regime – Equations of motion 25
   H. Dynamical backaction in the high-pump-power regime 26
   I. Photon-pressure induced transparency in the high-pump-power regime 26

6. Supplementary Note 6: Effects of dissipative coupling 26
   A. Dynamical backaction and cross-cooperativity 26
   B. Photon-pressure induced transparency 30
   C. The strong-coupling regime 31
   D. Impact of an additional external-dissipative coupling 33

7. Supplementary Note 7: Data analysis and fitting routines 35
   A. Background correction and basic fitting routine 35

B. PPIT fitting routine 37
C. LF reflection fitting routine for dynamical backaction 38
D. Photon number calibration 38
E. On-chip pump power calibration 39

8. Supplementary Note 8: Additional data 40
A. Resonant PPIT data for all pump powers 40
B. Dynamical backaction for all pump powers 40

9. Supplementary References 42

LIST OF MOST IMPORTANT PARAMETERS AND VARIABLES

Supplementary Table 1. **List of the most important basic variables used in the manuscript and supplementary material.**

Symbol	Meaning	Important relation to other quantities
Ω_0	Bare resonance frequency of the LF mode	–
Γ_{int}	Bare internal decay rate of the LF mode	–
Γ_{ext}	Bare external decay rate of the LF mode	–
Γ_0	Bare total decay rate of the LF mode	$\Gamma_0 = \Gamma_{int} + \Gamma_{ext}$
ω_0	Bare resonance frequency of the HF mode	–
κ_{int}	Bare internal decay rate of the HF mode	–
κ_{ext}	Bare external decay rate of the HF mode	–
κ_0	Bare total decay rate of the HF mode	$\kappa_0 = \kappa_{int} + \kappa_{ext}$
$g_{0\omega}$	Dispersive single-photon coupling rate	–
$g_{0\kappa}$	Dissipative single-photon coupling rate	–
ω_p	Frequency of the HF sideband pump tone	–
Δ_p	Detuning between pump tone and bare HF resonance	$\Delta_p = \omega_p - \omega_0$
P_p	On-chip power of the HF sideband pump tone	–
n_c	Intracavity pump-photon number in the HF mode	–
Ω_0'	Pump-shifted angular resonance frequency of the LF mode	$\Omega_0' = \Omega_0 + \mathcal{K}_c n_c$
$\mathcal{K}_c$	Cross-Kerr constant; LF dispersive shift per HF photon	–
Γ_0'	Pump-broadened total decay rate of the LF mode	$\Gamma_0' = \Gamma_0 + \kappa_c n_c$
κ_c	Cross-damping constant; LF linewidth-broadening per HF photon	–
ω_0'	Pump-shifted resonance frequency of the HF mode for a probe tone	$\omega_0' = \omega_p + \sqrt{\left(\Delta_p - \mathcal{K} n_c\right)\left(\Delta_p - 3\mathcal{K} n_c\right) - \frac{\kappa_{aux}^2}{4}}$
$\mathcal{K}$	Self-Kerr constant of the HF mode; frequency shift per photon	–
κ_0'	Pump-broadened linewidth of the HF mode for a probe tone	$\kappa_0' = \kappa_0 + 2\kappa_1 n_c + 3\kappa_2 n_c^2 + 4\kappa_3 n_c^3$
κ_m	m-th order nonlinear damping coefficient, $m \in \{1, 2, 3\}$	–
κ_{eff}	Pump-broadened linewidth of the HF mode for the pump tone	$\kappa_{eff} = \kappa_0 + \kappa_1 n_c + \kappa_2 n_c^2 + \kappa_3 n_c^3$
κ_{aux}	Auxiliary linewidth variable introduced for brevity	$\kappa_{aux} = \kappa_0' - \kappa_{eff}$
g_ω'	Pump- and nonlinearity-enhanced total dispersive coupling rate	$g_\omega' = g_{0\omega}\sqrt{n_c} + g_{0\mathcal{K}}\sqrt{n_c^3}$
$g_{0\mathcal{K}}$	Single-photon coupling rate due to Kerr nonlinearity	–
g_κ'	Pump- and nonlinearity-enhanced total dissipative coupling rate	$g_\kappa' = g_{0\kappa}\sqrt{n_c} + g_{nl1}\sqrt{n_c^3} + g_{nl2}\sqrt{n_c^5} + g_{nl3}\sqrt{n_c^7}$
g_{nlm}	Single-photon coupling rate due to m-th order nonlinear damping, $m \in \{1, 2, 3\}$	–
Δ_p'	Detuning between pump tone and pump-shifted HF probe resonance	$\Delta_p' = \omega_p - \omega_0'$
δ	Detuning between pump tone and bare HF mode red sideband	$\delta = \omega_p - (\omega_0 - \Omega_0)$
δ'	Detuning between pump tone and pump-shifted HF mode red sideband	$\delta' = \omega_p - \left(\omega_0' - \Omega_0'\right)$
$\delta\Omega_{pp}$	Photon-pressure backaction frequency shift	–
Ω_{eff}	LF resonance frequency including backaction and cross-Kerr shift	$\Omega_{eff} = \Omega_0' + \delta\Omega_{pp}$
Γ_{pp}	Photon-pressure backaction damping rate	–
Γ_{eff}	LF damping rate including backaction and cross-damping	$\Gamma_{eff} = \Gamma_0' + \Gamma_{pp}$
δ_{eff}	Detuning between pump tone and effective HF mode red sideband	$\delta_{eff} = \omega_p - \left(\omega_0' - \Omega_{eff}\right)$
χ_0	Bare LF mode susceptibility	$\chi_0 = \left[\Gamma_0/2 + i(\Omega - \Omega_0)\right]^{-1}$
χ_0^{eff}	Effective LF mode susceptibility including backaction and cross-effects	$\chi_0^{eff} = \left[\Gamma_{eff}/2 + i(\Omega - \Omega_{eff})\right]^{-1}$
χ_c	Bare HF mode susceptibility	$\chi_c = \left[\kappa_0/2 + i\left(\Delta_p + \Omega\right)\right]^{-1}$
χ_c'	Pump-modified HF mode susceptibility for a probe tone	$\chi_c' = \left[\kappa_0'/2 + i\left(\Delta_p' + \Omega\right)\right]^{-1}$

1. SUPPLEMENTARY NOTE 1: MEASUREMENT SETUP

During our experiments, we have used two different experimental setups, a simple one for pre-characterization of the device before the constriction Josephson junctions (cJJs) were milled into the circuit, and a more sophisticated one for after cJJ nano-patterning and for all the photon-pressure (PP) experiments. Both variants are schematically shown in Supplementary Fig. 1.

All experiments were conducted directly in liquid helium at the sample temperature $T_s \approx 4.2\,$K. The copper housing of the sample was always attached to a copper or brass mounting bracket at the end of a dipstick for microwave experiments in liquid helium transport dewars, cf. Supplementary Fig. 2 for pictures of the sample box and the dipstick tip of the more sophisticated setup. Both of the dipsticks we used have four coaxial lines and the whole sample space is enclosed in a ~30 cm long cryoperm magnetic shielding tube with a diameter of ~5 cm. The two circuits of the device, high-frequency (HF) and low-frequency (LF) circuit, were in both cases simultaneously connected to the coaxial lines by means of a directional coupler, which allowed us to measure the reflection S_{11} as transmission S_{21} using a vector network analyzer (VNA) and separate input and output lines, thus

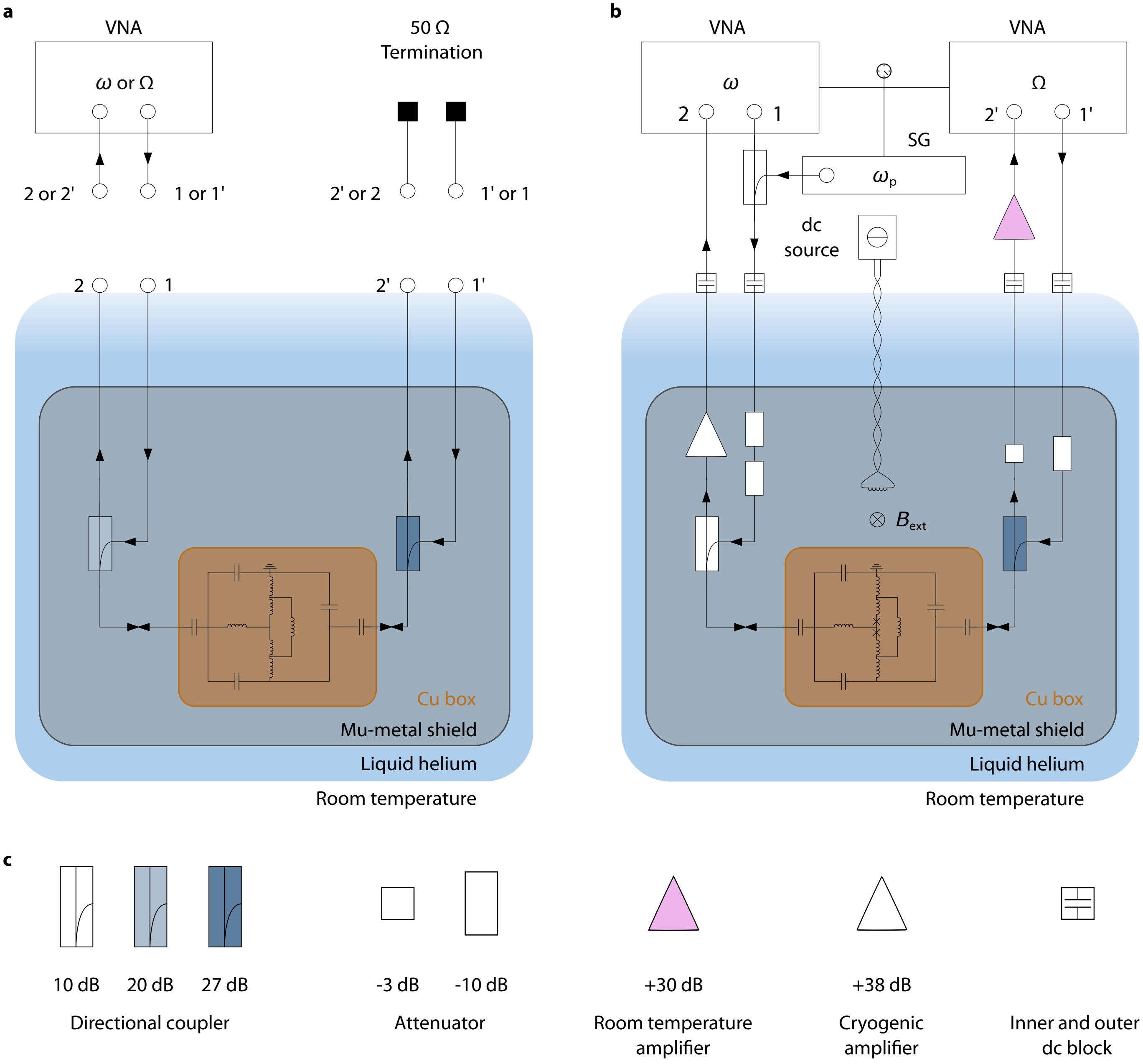

Supplementary Figure 1. **Schematics of the measurement setups.** **a** Setup for measurements without cJJs. **b** Setup for measurements with cJJs and for photon-pressure experiments. **c** Symbol legend for setup elements not directly labeled in **a**, **b**. Details are given in the text.

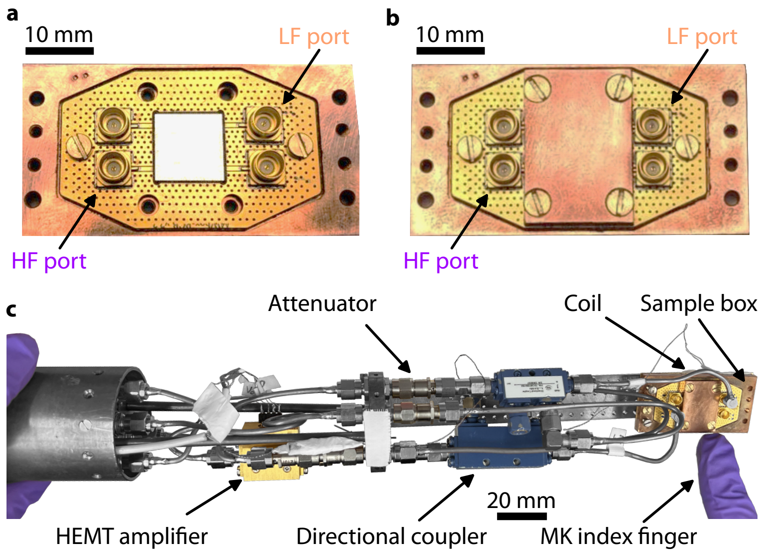

Supplementary Figure 2. **Photographs of sample box and dipstick tip.** **a** The square-shaped 10 mm × 10 mm large microchip with the PP device (image center, light gray) is mounted into the square-shaped cutout of a microwave PCB (golden) with four coplanar waveguide feedlines and four SMP connectors. Both chip and PCB are mounted onto the bottom part of the copper sample box; the chip is fixed by carbon tape and the PCB is fixed by screws. PCB and chip are thoroughly contacted by wirebonds all around the chip. The top right SMP connector leads to the on-chip feedline for the LF mode, the bottom left SMP connector to the feedline for the HF mode. **b** For the experiments, the chip is covered with a copper lid, which is fixed by four additional screws to the copper sample holder with chip and PCB as it is depicted in **a**. **c** Tip of the microwave dipstick for measurements in a liquid helium transport dewar. At the right end, the sample box shown in **b** is mounted with a small electromagnetic coil below it. Both HF and LF SMP ports are connected to copper coaxial cables. Also visible are the two directional couplers, multiple attenuators and the cryogenic HEMT amplifier. For clearer emphasis on the most important components, all microwave cables, dc wires and mounting parts are grayed out. Visible in purple are the gloved fingers of M. Kazouini.

minimizing the accumulation of parasitic reflection contributions in the measurement signal.

In the simpler measurements before cJJ-milling, cf. Supplementary Fig. 1**a**, we connected a VNA to either of the two circuits and terminated the two coaxial lines of the other circuit at the top of the dipstick at room temperature with a 50 Ω termination. Then, we either characterized the device response around the resonance frequency of the HF mode, i.e., at high frequencies ω, or around the resonance frequency of the LF mode, i.e., at low frequencies Ω.

Once the cJJs are introduced and we characterize the photon-pressure interaction, the setup gets much more sophisticated, cf. Supplementary Fig. 1**b** and Supplementary Fig. 2**c**. Firstly, we need a variable magnetic field $B_{\rm ext}$ perpendicular to the chip surface to change the magnetic flux in the superconducting quantum interference device (SQUID) and to pick the ideal operation point. To this end, a homemade electromagnetic coil is attached to the copper housing of the sample, which is connected by a pair of twisted copper wires to a battery-powered direct current (dc) source at room-temperature. Secondly, we need to minimize incoming thermal noise on the coaxial lines and to equilibrate the equivalent temperature of that noise to ~4.2 K, at least on the HF side of the device. The LF mode is much less nonlinear than the HF mode due to the circuit design, and since at the time of this experiment we did not have a cryogenic amplifier for the frequencies of the LF mode, we could not completely attenuate the corresponding coaxial lines. Hence, the HF input line is attenuated at 4.2 K with two 10 dB attenuators and additionally with a ~10 dB directional coupler. After reflection at the HF circuit, the signal is routed straight through the directional coupler to a cryogenic high-electron-mobility-transistor (HEMT) amplifier with a gain of ~38 dB. The LF input line has a single 10 dB attenuator in series with additional 27 dB attenuation at its directional coupler. The output line on the other hand has a small attenuator with 3 dB to provide at least some more attenuation of input noise than just the coaxial cable damping, while preserving the detectability of the reflected signal. At room temperature, the reflected LF signal is also amplified by a HEMT amplifier with a gain of ~30 dB to improve the signal-to-noise ratio. Note that all four coaxial lines in the photon-pressure setup are made of stainless steel cables (and short copper cables in the cryogenic region), which provide additional attenuation of 5 dB to 10 dB per line. Furthermore, directly at the top of the dipstick on the coaxial feedthrough flange, four dc blocks were inserted into the four coaxial lines.

Regarding the microwave electronics, we conducted all experiments after cJJ-cutting with a single setup. For the spectroscopic characterization of the HF and LF reflection, we permanently had two independent VNAs connected to the two device modes. In addition, we connected a microwave signal generator (SG), that provided the sideband-pump tone for the photon-pressure experiments. The SG pump tone with frequency $\omega_{\rm p}$ is combined with the VNA input signal of the HF circuit by means of a directional coupler at room temperature. The three microwave electronics components were referenced to the 10 MHz oscillator

of the signal generator.

All active electronics except for the statically operated HEMT amplifiers are controlled and read out during the measurements via a single computer, using Python-based measurement scripts.

2. SUPPLEMENTARY NOTE 2: THE INDIVIDUAL CIRCUITS WITHOUT CONSTRICTIONS

We describe both circuits by lumped element parallel RLC circuits, that are capacitively coupled to a single transmission line with characteristic impedance Z_0.

A. The high-frequency circuit without constrictions

The high-frequency circuit without the constrictions consists of two interdigitated capacitors C_{idc} that we formally combine into a single capacitance $C = 2C_{\mathrm{idc}}$, and of several inductors, that we combine into a single inductance L. The two small parts of the loop, into which the constrictions are milled, have each the inductance L_{arm} and therefore the high-frequency inductance without these bridges would be given by $L' = L - L_{\mathrm{arm}}/2$. The total loop inductance we denote as L_{loop} and we estimate $L_{\mathrm{arm}} \approx L_{\mathrm{loop}}/10$. By means of a coupling capacitor C_{c}, the circuit is capacitively coupled to its coplanar waveguide feedline with characteristic impedance Z_0. All internal losses we summarize in a resistor R. Using these parameters and assuming a low-loss oscillator, the resonance frequency of the junction-less circuit is given by

$$\omega_{\mathrm{b}} = \frac{1}{\sqrt{LC_{\mathrm{tot}}}}, \qquad C_{\mathrm{tot}} = C + C_{\mathrm{c}} \tag{S1}$$

and the internal and external linewidths by

$$\kappa_{\mathrm{int,b}} = \frac{1}{RC_{\mathrm{tot}}}, \qquad \kappa_{\mathrm{ext,b}} = \frac{Z_0 C_{\mathrm{c}}^2}{LC_{\mathrm{tot}}^2}. \tag{S2}$$

B. The low-frequency circuit without constrictions

The low-frequency circuit consists of a parallel plate capacitance C_0 and an effective inductance L_0, and it is coupled by means of a coupling capacitor C_{c0} to its coplanar waveguide feedline, also with characteristic impedance Z_0. Analogous to the high-frequency case, we can write the relevant relations as

$$\Omega_{\mathrm{b}} = \frac{1}{\sqrt{L_0 C_\Sigma}}, \qquad C_\Sigma = C_0 + C_{\mathrm{c0}} \tag{S3}$$

and

$$\Gamma_{\mathrm{int,b}} = \frac{1}{R_0 C_\Sigma}, \qquad \Gamma_{\mathrm{ext,b}} = \frac{Z_0 C_{\mathrm{c0}}^2}{L_0 C_\Sigma^2}. \tag{S4}$$

C. Circuit parameters

We determine the circuit parameters starting from the capacitances C_0 and C. The low-frequency parallel plate capacitance is calculated by

$$C_0 = \epsilon_0 \epsilon_{\mathrm{r}} \frac{A}{d_{\mathrm{SiN}}} \tag{S5}$$

and with the silicon-nitride dielectric constant $\epsilon_{\mathrm{r}} \approx 7$, the silicon-nitride thickness $d_{\mathrm{SiN}} \approx 200\,\mathrm{nm}$, and the capacitor area $A = 1.583\,\mathrm{mm}^2$ we find $C_0 = 490.5\,\mathrm{pF}$. The high-frequency capacitance we obtain using the analytical equations of Ref. [1] for interdigitated capacitors (IDCs), given by

$$C_{\mathrm{idc}} = (N_{\mathrm{idc}} - 3)\,\frac{C_1}{2} + 2\frac{C_1 C_2}{C_1 + C_2} \tag{S6}$$

$$C_i = 2\epsilon_0 \epsilon_{\mathrm{eff}} l_{\mathrm{idc}} \frac{K(k_i)}{K(k_i')}, \qquad i = 1, 2 \tag{S7}$$

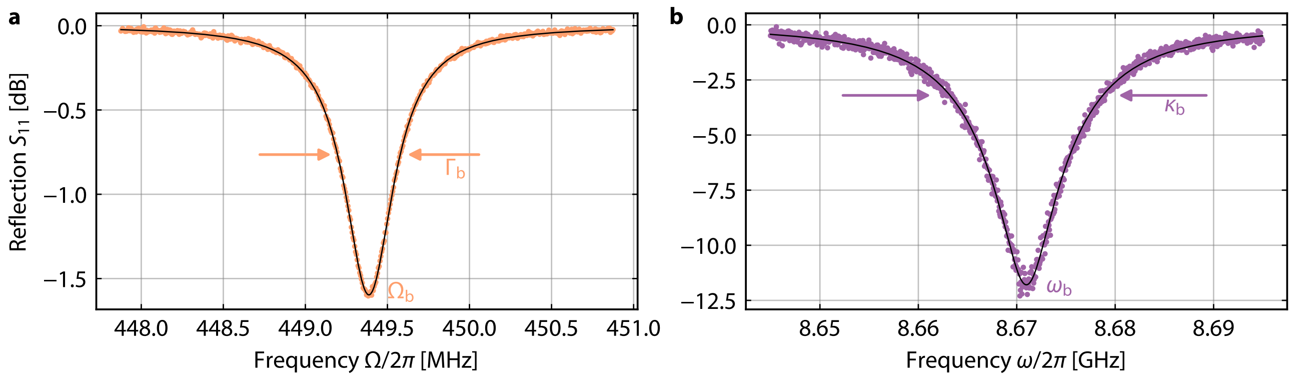

Supplementary Figure 3. **Device resonances before cutting the nano-constrictions.** **a** Reflection S_{11} of the low-frequency (LF) circuit around its resonance frequency. Orange symbols are data, black line is a fit. From the fit curve, we obtain the resonance frequency before constriction cutting $\Omega_b = 2\pi \times 449.4$ MHz and the total linewidth $\Gamma_b = 2\pi \times 401$ kHz. **b** Reflection S_{11} of the high-frequency (HF) circuit around its resonance frequency. Purple symbols are data, black line is a fit. From the fit curve, we obtain the resonance frequency before constriction cutting $\omega_b = 2\pi \times 8.671$ GHz and the total linewidth $\kappa_b = 2\pi \times 14.8$ MHz. Internal and external contributions to Γ_b and κ_b can be found in the text and in Supplementary Tables 2 and 3.

where $K(k_i)$ are complete elliptic integrals of the first kind, $l_{\rm idc} = 210\,\mu$m is the finger length, $\epsilon_{\rm eff} = (\epsilon_{\rm Si} + 1)/2$ is the effective permittivity with the silicon permittivity $\epsilon_{\rm Si} = 11.8$, and $N_{\rm idc} = 48$ is the total number of fingers in the capacitor. The remaining parameters k_i are given by

$$k_1 = \sin\left(\frac{\pi}{2}\frac{a}{a+b}\right) \tag{S8}$$

$$k_2 = 2\frac{\sqrt{a(a+b)}}{2a+b} \tag{S9}$$

$$k_i' = \sqrt{1-k_i^2} \tag{S10}$$

with $a = 3\,\mu$m the finger width and $b = 5\,\mu$m the gap width in between two fingers. Finally, we obtain $C = 2C_{\rm idc} = 941.5$ fF. Similarly, we calculate the coupling capacitors as $C_c = 15.3$ fF by the IDC model and $C_{c0} = 559$ fF using the parallel plate expression.

Next, we measure the resonance frequencies $\omega_b = 2\pi \times 8.671$ GHz and $\Omega_b = 2\pi \times 449.4$ MHz, cf. Supplementary Fig. 3 for the two resonance datsets. In combination with $C_{\rm tot} = 956.8$ fF and $C_\Sigma = 491$ pF we calculate $L = 352$ pH and $L_0 = 255$ pH. From the fits to the reflection data, we furthermore obtain the linewidths $\Gamma_{\rm int,b} = 2\pi \times 367$ kHz, $\Gamma_{\rm ext,b} = 2\pi \times 34$ kHz, $\kappa_{\rm int,b} = 2\pi \times 5.1$ MHz and $\kappa_{\rm ext,b} = 2\pi \times 9.7$ MHz. With the characteristic impedance $Z_0 = 50\,\Omega$, we can also calculate theoretical numbers for $\Gamma^{\rm th}_{\rm ext,b} = 2\pi \times 40$ kHz and $\kappa^{\rm th}_{\rm ext,b} = 2\pi \times 5.76$ MHz using Eqs. (S2) and (S4). The agreement with the experimental result is not extremely high, but acceptable. Deviations between theory and experiment in the external coupling rates are very common and can be explained by stray capacitances, parasitic reflections (e.g. at the crossover from cable to PCB or from PCB to chip), impedance mismatches in the microwave cabling leading to the sample or interferences by the imperfect isolation e.g. in the directional couplers, cf. Refs. [2, 3].

Finally, we use the software package *3D-MLSI* [4] with the London penetration depth $\lambda_L = 130$ nm (typical value for our Nb films at 4.2 K) to calculate the SQUID loop self-inductance $L^{\rm sim}_{\rm loop} = 19.3$ pH. The loop has a rectangular shape, the inner hole is $11 \times 6\,\mu$m^2 large, the outer dimensions are $17 \times 12\,\mu$m^2. Supplementary Tables 2 and 3 summarize all the relevant parameters of our device before constriction cutting and as determined from theoretical calculations, simulations and experiments. Note that these values are good estimates, but not exact, since there are various sources of uncertainty (ϵ_r, $d_{\rm SiN}$, $\epsilon_{\rm eff}$ or λ_L) and we also observe that the resonance frequencies of our devices can change from cooldown to cooldown on the order $0.1\% - 0.5\%$. Hence, we will not fix the values of the circuit elements rigidly in our further analyses except for the capacitances, and allow the inductances to slightly vary in the description of different cooldowns/experiments. We label the preliminary inductances $L = L^{\rm pre}$ and $L_0 = L_0^{\rm pre}$ in the tables.

Supplementary Table 2. **Preliminary circuit parameters of the LF mode estimated before cutting the nanobridge junctions.**

$A\,[\mathrm{mm}^2]$	d_SiN [nm]	C_0 [pF]	C_c0 [fF]	L_0^pre [pH]	$\Omega_\mathrm{b}/2\pi$ [MHz]	$\Gamma_\mathrm{int,b}/2\pi$ [kHz]	$\Gamma_\mathrm{ext,b}/2\pi$ [kHz]
1.583	200	490.5	559	255.4	449.4	367	34

Supplementary Table 3. **Preliminary circuit parameters of the HF mode estimated before cutting the nanobridge junctions.**

N_idc	l_idc [µm]	a [µm]	b [µm]	C [fF]	C_c [fF]	L^pre [pH]	$\omega_\mathrm{b}/2\pi$ [GHz]	$\kappa_\mathrm{int,b}/2\pi$ [MHz]	$\kappa_\mathrm{ext,b}/2\pi$ [MHz]	$L_\mathrm{loop}^\mathrm{sim}$ [pH]
48	210	3	5	941.5	15.3	352.1	8.671	5.1	9.7	19.3

3. SUPPLEMENTARY NOTE 3: THE INDIVIDUAL CIRCUITS WITH CONSTRICTIONS

A. Including the constrictions

Our treatment is analogous to the descriptions given in Refs. [5–7]. For low microwave powers we treat each constriction as a bias-flux-dependent but linear inductance L_c, for high powers we add a Kerr nonlinearity and nonlinear damping. It is a very simple but quite accurate approach to describe the constriction inductance as a series combination of a linear inductance L_lin and a Josephson inductance L_J, i.e.,

$$L_\mathrm{c} = L_\mathrm{lin} + L_\mathrm{J} \tag{S11}$$

where the linear contribution considers deviations from the sinusoidal current-phase-relation [7, 8] and the Josephson part is given by

$$L_\mathrm{J} = \frac{\Phi_0}{2\pi I_0 \cos\delta_0} \tag{S12}$$

with δ_0 the bias-flux-induced equilibrium phase difference, I_0 the critical current of the cJJ, and $\Phi_0 \approx 2.068\times10^{-15}\,\mathrm{T\,m^2}$ the flux quantum. The equilibrium phase difference is related to the total flux Φ in the SQUID by

$$\delta_0 = \pi\frac{\Phi}{\Phi_0}, \tag{S13}$$

and so the Josephson inductance of a single constriction is

$$L_\mathrm{J} = \frac{\Phi_0}{2\pi I_0 \cos\left(\pi\frac{\Phi}{\Phi_0}\right)}. \tag{S14}$$

In general, the constrictions also change the internal decay rate of the two modes, which could be considered by a (flux-dependent) constriction resistance R_c in parallel to L_c. Since the impact of this resistance on the resonance frequency is typically very small in our parameter regime [7], we omit it for all inductive calculations and phenomenologically model the linewidth as a function of flux by a polynomial.

B. Describing the SQUID

To describe the flux-biased SQUID, we start with the flux equation for a symmetric SQUID without bias current, which is given by

$$\frac{\Phi}{\Phi_0} = \frac{\Phi_\mathrm{b}}{\Phi_0} + \frac{\tilde{L}_\mathrm{loop}J}{\Phi_0} \tag{S15}$$

where Φ_b is the external flux threading the SQUID loop, J is the induced circulating current and $\tilde{L}_\mathrm{loop} = L_\mathrm{loop} + 2L_\mathrm{lin}$ is the effective linear loop inductance, which contains both geometric and kinetic contributions.

Without a bias current and for two identical junctions, the circulating current is described by

$$J = -I_0 \sin\left(\pi\frac{\Phi}{\Phi_0}\right). \tag{S16}$$

The minus sign in this relation can be intuitively understood as follows: for small but finite external flux $0 < \Phi_\mathrm{b}/\Phi_0 < 0.5$ (and zero flux trapped in the SQUID loop), the screening-current flux $\tilde{L}_\mathrm{loop}J$ reduces the total flux in the SQUID compared to the external flux, i.e., it must be $J < 0$ to satisfy the condition $\Phi < \Phi_\mathrm{b}$. Since also $0 < \Phi/\Phi_0 < 0.5$ in this situation we find $\sin(\pi\Phi/\Phi_0) > 0$ and the condition $J < 0$ can only be fulfilled with the minus sign in Eq. (S16).

With the screening parameter $\beta_L = 2\tilde{L}_\mathrm{loop}I_0/\Phi_0 = \tilde{L}_\mathrm{loop}/\pi L_{\mathrm{J}0}$ and $L_{\mathrm{J}0} = \Phi_0/2\pi I_0$ we can finally re-write the flux relation Eq. (S15) as

$$\frac{\Phi}{\Phi_0} = \frac{\Phi_\mathrm{b}}{\Phi_0} - \frac{\beta_L}{2}\sin\left(\pi\frac{\Phi}{\Phi_0}\right). \tag{S17}$$

For each external flux Φ_b, we numerically solve this equation to find the total flux Φ, and subsequently the corresponding Josephson inductance $L_\mathrm{J}(\Phi)$.

C. The total circuit including constrictions – Dispersive part

To model the complete circuit including the constrictions, we separate the inductances into multiple parts, cf. Supplementary Fig. 4. First, we take a part of the HF inductance as not contributing to the LF mode and label this part with L_1. Then we add two symmetric inductors L_2, which contribute to both the HF and the LF circuit. Note, however, that they contribute in series to the LF mode and in parallel to the HF mode. The most complicated part is the SQUID loop including the cJJs. In combination with the linear part of the constrictions, each loop part with a constriction has the linear inductance $L_\mathrm{lin} + L_\mathrm{arm} \approx L_\mathrm{lin} + 0.1L_\mathrm{loop}$, and the total inductance

$$L_\mathrm{p} = L_\mathrm{J} + L_\mathrm{lin} + 0.1L_\mathrm{loop}. \tag{S18}$$

The large part of the loop without the constrictions has then the inductance $L_\mathrm{l} \approx 0.8L_\mathrm{loop}$, and we will assume that this part does not contribute significantly to the HF mode inductance, since the HF currents will not significantly pass through that loop

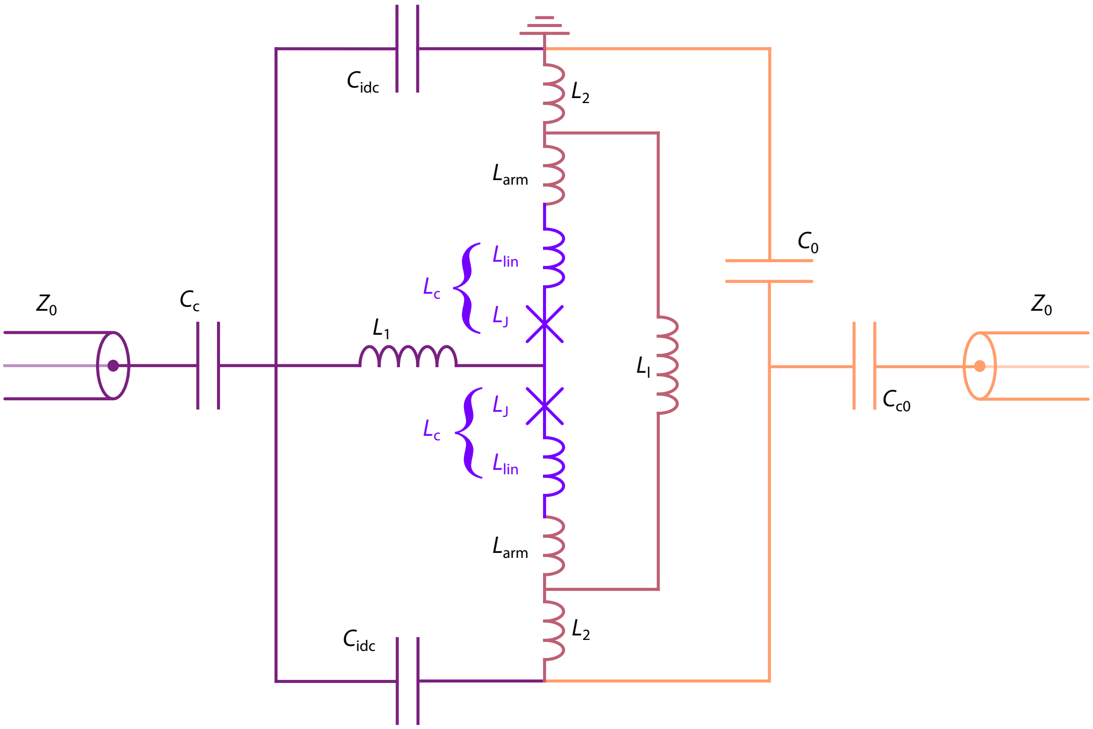

Supplementary Figure 4. **The complete equivalent circuit of the device.** The low-frequency mode (right half) comprises a capacitor C_0 and the inductive branch, consisting of all inductors except for L_1. The mode is capacitively coupled to a feedline with characteristic impedance Z_0 by means of a coupling capacitance C_c0. The high-frequency mode (left half) has two interdigitated capacitors C_idc, the linear inductance L_1 and a parallel combination of two composite inductive branches. The mode is coupled to a Z_0-feedline by a coupling capacitance C_c. The SQUID loop has the inductance $L_\mathrm{loop} = L_\mathrm{l} + 2L_\mathrm{arm}$, each constriction contributes $L_\mathrm{c} = L_\mathrm{J} + L_\mathrm{lin}$ and the two modes share the inductors L_2, which connect from top and bottom to the SQUID loop. More details and the values for all inductors can be found in the text and in Supplementary Table 4.

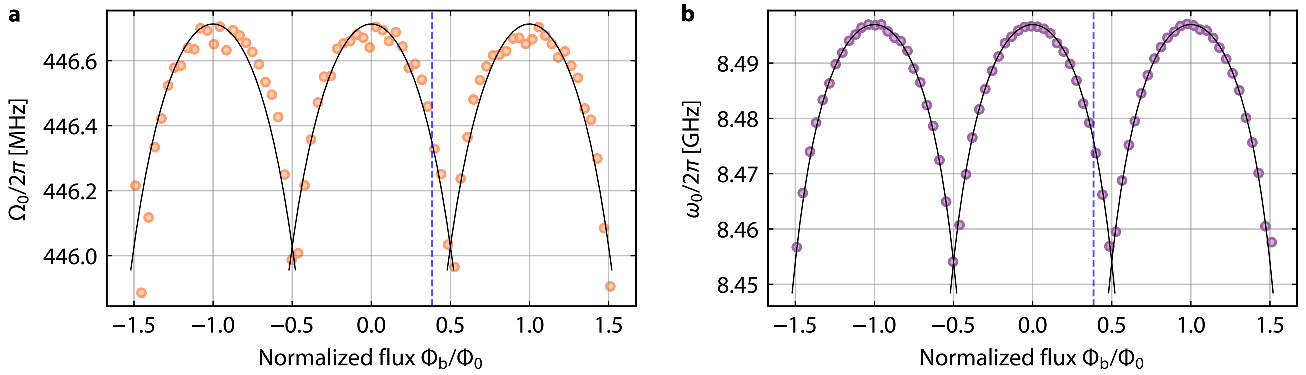

Supplementary Figure 5. **Flux-tuning of LF and HF resonance frequencies.** **a** Resonance frequency of the LF mode Ω_0 vs. normalized bias flux Φ_b/Φ_0. Total tuning range is ~700 kHz. **b** Resonance frequency of the HF mode ω_0 vs. normalized bias flux Φ_b/Φ_0. Total tuning range is ~43 MHz. In both panels symbols are data and lines are a simultaneous fit of both modes with a single parameter set. From the fit, we obtain the parameters $L = 355.2$ pH, $L_0 = 249.4$ pH, $L_{\text{loop}} = 21.5$ pH, $L_{\text{lin}} = 6.5$ pH and $L_{\text{J0}} = 16.5$ pH. Blue dashed vertical lines indicate the operation point $\Phi_b/\Phi_0 = 0.385$ for all photon-pressure experiments in this work.

section. Subsequently, we get for the total inductances of LF and HF mode, respectively,

$$L_{\text{LF}} = 2L_2 + \frac{1.6L_{\text{p}}L_{\text{loop}}}{2L_{\text{p}} + 0.8L_{\text{loop}}} \tag{S19}$$

$$L_{\text{HF}} = L_1 + \frac{L_{\text{p}} + L_2}{2}. \tag{S20}$$

Plugging these relations into the equations for the resonance frequencies

$$\omega_0 = \frac{1}{\sqrt{C_{\text{tot}}L_{\text{HF}}}} \tag{S21}$$

$$\Omega_0 = \frac{1}{\sqrt{C_\Sigma L_{\text{LF}}}} \tag{S22}$$

allows us to apply a combined, complex-valued fit function

$$\tilde{\omega}_0 = \omega_0(\Phi) + \mathrm{i}\Omega_0(\Phi) \tag{S23}$$

to both datasets of flux-dependence. Supplementary Fig. 5 shows the resonance frequency of both modes as a function of bias flux Φ_b and the corresponding simultaneous fit curves. In practice we multiply the LF resonance Ω_0 by a factor 19 (both dataset and theory expression) for the fitting, in order to bring both resonances to the same frequency scale and to get identical weights for the fits from both modes.

For the fit, we keep C_{tot} and C_Σ fixed from the calculations and we fit for $L, L_0, L_{\text{loop}}, L_{\text{lin}}$ and L_{J0} (in practice I_0), which together determine β_L and the flux-dependence of ω_0 and Ω_0. From the fit, we get $L = 355.2$ pH, $L_0 = 249.4$ pH, $L_{\text{loop}} = 21.5$ pH, $L_{\text{lin}} = 6.5$ pH and $L_{\text{J0}} = 16.5$ pH, which corresponds to a critical constriction current $I_0 = 20\,\mu$A. The corresponding screening parameter is $\beta_L = 0.67$. By inverting above relations, we can also get the values for $L_1 = 292.7$ pH and $L_2 = 123$ pH. Supplementary Table 4 summarizes all the relevant parameters of this section, i.e., all inductances of the device as extracted from the flux-tuning fits. The values for L and L_0 differ from the original ones obtained from the measurements before constriction-cutting by 0.6% and −1.9%, respectively, which is an acceptable and non-significant deviation considering our typical fluctuations between different cooldowns. Additionally the device has been re-wirebonded after the cJJ cutting, which can also modify the effective feedline impedance and contribute to small frequency shifts.

Supplementary Table 4. **Best-fit inductance parameters extracted from a simultaneous fit of the frequency flux-tuning curves.**

L [pH]	L_0 [pH]	L_1 [pH]	L_2 [pH]	L_{lin} [pH]	L_{J0} [pH]	I_0 [μA]	L_{loop} [pH]	β_L
355.2	249.4	292.7	123	6.5	16.5	20	21.5	0.67

D. The total circuit including constrictions – Dissipative part

The introduction of the constrictions not only changes the resonance frequencies of the circuits and makes them flux-tunable, also the circuit linewidths are impacted similarly. The origin of the additional dissipation is likely thermal quasiparticles in the constrictions, since the cJJs have a reduced transition temperature $T_{cc} < T_c$ compared to the critical temperature of the bare niobium film $T_c \approx 9\,$K [7, 8]. All our measurements have been taken at a sample temperature $T_s = 4.2\,$K in a liquid helium bath. At the flux sweetspots, the fit-curve linewidths we find with the constrictions are $\Gamma_{ext} = 2\pi \times 37\,$kHz, $\Gamma_{int} = 2\pi \times 503\,$kHz, $\kappa_{ext} = 2\pi \times 6.7\,$MHz and $\kappa_{int} = 2\pi \times 38\,$MHz, for the corresponding S_{11} data of one specific dataset cf. main paper Fig. 1. As expected, mainly the internal linewidths are affected by the constriction cutting. The external linewidths stay roughly the same as before the cutting, and the differences once again might be related to interferences and impedance mismatches in the cabling; the change from before to after cutting could either be due to the circuits having different resonance frequencies after the cutting (interferences e.g. are frequency-dependent), or because the chip has been taken out of the sample holder for the constriction fabrication and afterwards was re-wirebonded to the PCB, a process that is never completely identical when repeated. Interestingly, the HF external linewidth κ_{ext} is now much closer to its theoretical value than before the cutting.

When a magnetic flux is applied to the SQUID, we observe that the linewidths modulate periodically with flux, similar to earlier observations in niobium Ne-FIB constriction circuits [7, 8]. The extracted flux-dependent linewidths $\Gamma_{int}, \Gamma_{ext}$ and $\kappa_{int}, \kappa_{ext}$ are shown as a function of Φ_b/Φ_0 in Supplementary Fig. 6. Mainly the internal circuit linewidths are modulating, which is consistent with the initial change of predominantly the internal linewidth by the cJJ patterning and its interpretation based on thermal quasiparticles, which are now additionally increased by the screening current when the SQUID is flux biased. The internal linewidth of the HF circuit increases from $\kappa_{int}/2\pi \approx 38\,$MHz at the flux-sweetspot to $\sim$118 MHz at half-integer flux values. The external HF linewidth increases in the same flux range from $\kappa_{ext}/2\pi = 6.7\,$MHz at the sweetspot to $\sim$7.6 MHz. However, the seeming flux-tuning of κ_{ext} is most likely an artifact of an imperfect fit and not an actual flux-dependence of the external decay rate. In Ref. [3] it was shown, that the smaller one of the two linewidths κ_{int} and κ_{ext}, which here is κ_{ext}, will have a considerable uncertainty in the case that $\kappa_{int}/\kappa_{ext}$ deviates significantly from 1. This would mean, that easily $10\% - 20\%$ of what we obtain as κ_{ext} could actually be a part of κ_{int}, which is misattributed to κ_{ext} in the fitting routine by the presence of e.g. a small leakage signal in the directional coupler. If this small part follows the same trend as κ_{int}, i.e. triples by flux, it can explain the seeming increase of κ_{ext} by 0.9 MHz with flux without the external decay rate actually being flux-dependent.

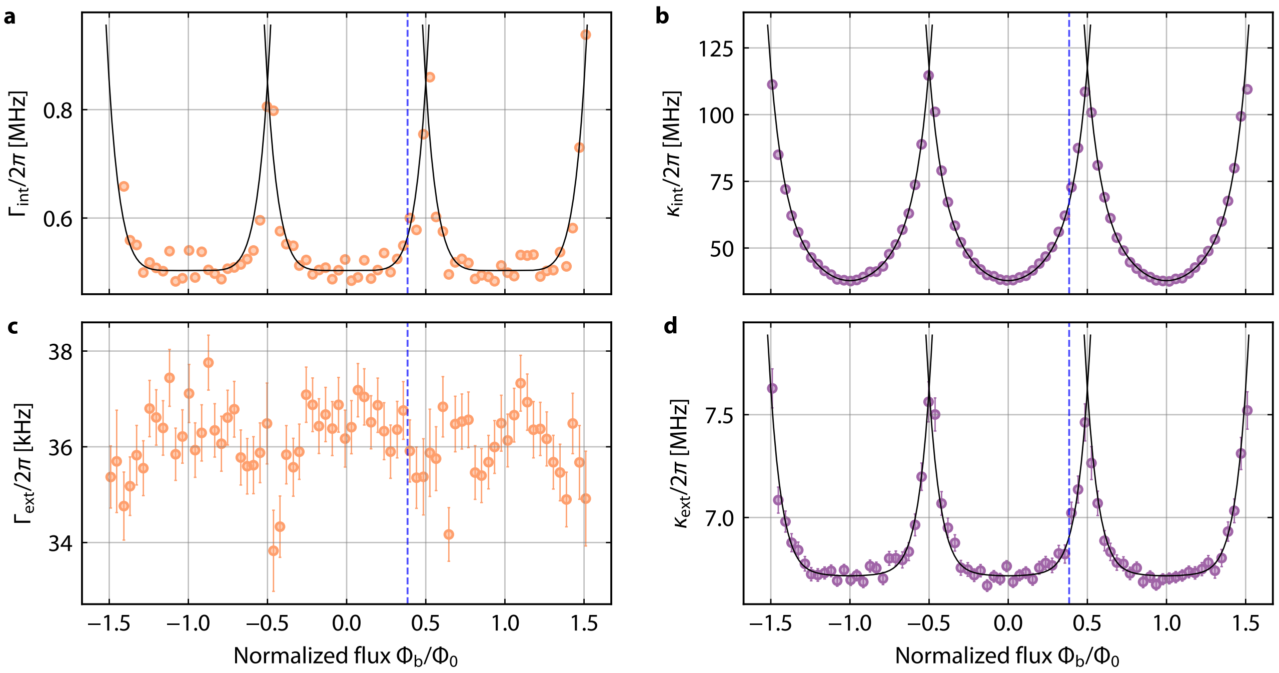

Supplementary Figure 6. **Change of linewidths with flux through the SQUID.** The internal and external linewidths of HF and LF circuit as a function of normalized external flux Φ_b/Φ_0 through the SQUID. **a** and **c** show internal and external linewidth of the LF circuit Γ_{int} and Γ_{ext}, respectively. **b** and **d** show the internal and external linewidths of the HF mode κ_{int} and κ_{ext}, respectively. Symbols are data extracted from fits of the corresponding resonances, lines are fits with Eqs. (S24, S25, S26). Error bars are the standard errors obtained from the fit. Blue dashed vertical line in each panel indicates the operation point $\Phi_b/\Phi_0 = 0.385$ for all photon-pressure experiments in this work.

Alternatively, the interference between the actual reflection and the leakage signal itself and the small fraction of $\kappa_{\rm int}$, that is falsely attributed to $\kappa_{\rm ext}$, can be frequency-dependent, leading to similar consequences. If $\kappa_{\rm ext}$ really was changing with flux in the SQUID, i.e., with the resonance frequency of the HF mode, we would expect to see a similar trend of increasing $\kappa_{\rm ext}$ with decreasing ω_0, when the HF mode is frequency-shifted by a pump tone instead of by flux. However, when we do so at the operation point during the PPIT (photon-pressure induced transparency) experiments, we find a nearly constant $\kappa_{\rm ext}$ with a gentle trend to even decrease instead of increase [9]. The fact that the fitting procedure for the PPIT data slightly deviates from the one used for the flux arc response data (e.g. by cutting out the PPIT window, cf. Supplementary Note 7) leads us to the conclusion that it is indeed an artifact, that depends on the exact fit protocol. Note, that just based on the theoretical expression for the external decay rate $\kappa_{\rm ext} = \omega_0^2 Z_0 C_c^2/C_{\rm tot}$, one would actually expect a decrease of $\kappa_{\rm ext}$ by ~1% over the flux range from 0 to $0.5\Phi_0$ instead of the seeming increase by ~12%, but such a small shift would be below our analysis accuracy.

Since in the LF circuit the cJJs are in parallel to a second junction-free inductance, the impact of their presence and their flux-tuning is smaller. The internal LF linewidth increases from $\Gamma_{\rm int}/2\pi = 503\,$kHz at the sweetspot to $\Gamma_{\rm int}/2\pi \approx 851\,$kHz at the anti-sweetspot. The external LF linewidth is almost constant and only slightly modulates around $\Gamma_{\rm ext}/2\pi \approx 36\,\text{kHz} \pm 2\,\text{kHz}$, which again is likely impacted by signal interferences and the fitting routine for the strongly undercoupled circuit with $\Gamma_{\rm ext}/\Gamma_{\rm int} \ll 1$.

We fit the internal linewidths and the external HF linewidth for a single flux arc with sixth-order even polynomials

$$\kappa_{\rm int}(\Phi_b) = \kappa_{\rm int,ss} + \kappa_{\rm int,2}\Phi_b^2 + \kappa_{\rm int,4}\Phi_b^4 + \kappa_{\rm int,6}\Phi_b^6 \tag{S24}$$

$$\kappa_{\rm ext}(\Phi_b) = \kappa_{\rm ext,ss} + \kappa_{\rm ext,2}\Phi_b^2 + \kappa_{\rm ext,4}\Phi_b^4 + \kappa_{\rm ext,6}\Phi_b^6 \tag{S25}$$

$$\Gamma_{\rm int}(\Phi_b) = \Gamma_{\rm int,ss} + \Gamma_{\rm int,2}\Phi_b^2 + \Gamma_{\rm int,4}\Phi_b^4 + \Gamma_{\rm int,6}\Phi_b^6 \tag{S26}$$

where $\kappa_{\rm int,ss}, \kappa_{\rm ext,ss}, \Gamma_{\rm int,ss}$ are the sweetspot values and all $\kappa_{{\rm int},j}, \kappa_{{\rm ext},j}, \Gamma_{{\rm int},j}$ for even $j > 1$ are fit parameters. The resulting fit lines (and their replicas at $\pm 1\Phi_0$) are shown in Supplementary Fig. 6. The level of agreement with the data, in particular with that of the HF mode, is satisfactory. For $\Gamma_{\rm ext}$ we did not add a fit curve, since there is too little actual modulation and since we nowhere need it. At the photon-pressure operation point of this work $\Phi_b/\Phi_0 \approx 0.385$, marked in Supplementary Fig. 6 with dashed vertical lines, we find for the four linewidths $\kappa_{\rm int}/2\pi = 67\,$MHz, $\kappa_{\rm ext}/2\pi = 7\,$MHz, $\Gamma_{\rm int}/2\pi = 565\,$kHz and $\Gamma_{\rm ext}/2\pi = 36\,$kHz.

E. Single-photon coupling rates

As a last step in the estimation of the device parameters, we calculate the two single-photon coupling rates

$$g_{0\omega} = -\frac{\partial\omega_0}{\partial\Phi_b}\Phi_{\rm zpf} \tag{S27}$$

$$g_{0\kappa} = -\frac{\partial\kappa_0}{\partial\Phi_b}\Phi_{\rm zpf} \tag{S28}$$

where $\Phi_{\rm zpf}$ is the zero-point flux coupled into the SQUID loop by the LF resonator. The two derivatives (responsivities) we obtain numerically from the fits of the $\omega_0(\Phi_b)$ and $\kappa_0(\Phi_b)$ flux arcs, cf. main paper Fig. 2. The zero-point flux on the other hand we calculate through the zero-point current of the LF mode

$$I_{\rm zpf} = \sqrt{\frac{\hbar\Omega_0}{2L_{\rm LF}}} \approx 23.9\,\text{nA}. \tag{S29}$$

Next, we use the flux equation for an asymmetric SQUID, biased with a current $I_{\rm zpf}$, which flows along the LF inductance,

$$\Phi = \Phi_b + \tilde{L}_{\rm loop}J - \alpha_L\tilde{L}_{\rm loop}\frac{I_{\rm zpf}}{2} \tag{S30}$$

where the inductive asymmetry parameter is

$$\alpha_L = \frac{2L_{\rm lin} + 2L_{\rm arm} - \left(L_{\rm loop} - 2L_{\rm arm}\right)}{\tilde{L}_{\rm loop}} \tag{S31}$$

$$= \frac{4(L_{\rm lin} + L_{\rm arm}) - \tilde{L}_{\rm loop}}{\tilde{L}_{\rm loop}}, \tag{S32}$$

i.e., in our case $\alpha_L \approx -0.058$. Note, that the bias current $I_{\rm zpf}$ flows unusually through the three-terminal SQUID considered here: one SQUID arm for this current contains no cJJ, while the second SQUID arm contains both cJJs.

Using the expression for the circulating current with a bias current $I_{\rm zpf}$

$$J = -I_0 \sin\left(\pi\frac{\Phi}{\Phi_0}\right) + \frac{I_{\rm zpf}}{2} \tag{S33}$$

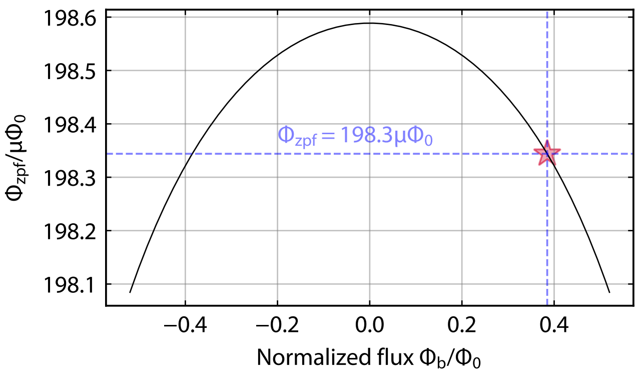

Supplementary Figure 7. **Zero-point-fluctuation amplitude of effective LF flux coupling into the SQUID.** Line shows the zero-point-flux fluctuation amplitude of the LF mode calculated via Eq. (S36). It is only weakly dependent on Φ_b and at the operation point of this work $\Phi_\mathrm{b}/\Phi_0 = 0.385$ (marked with dashed lines and a star symbol), it is $\Phi_\mathrm{zpf} \approx 198.3\,\mu\Phi_0$.

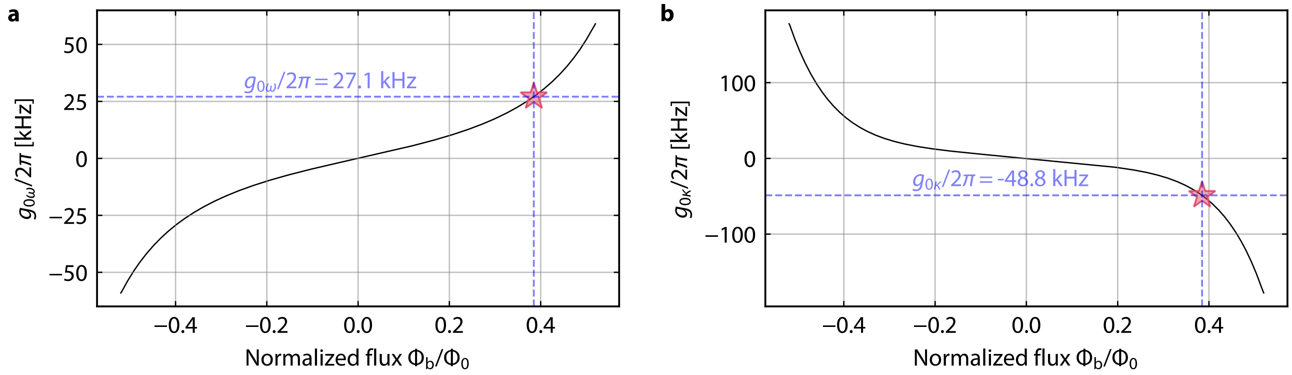

Supplementary Figure 8. **Dispersive and dissipative single-photon coupling rates.** From the flux-tuning fits of ω_0 and κ_0, we numerically calculate their derivatives and by multiplication with Φ_zpf we obtain the photon-pressure single-photon coupling rates $g_{0\omega}$ and $g_{0\kappa}$, respectively. The former is the dispersive coupling rate and shown in panel **a**, the latter is the dissipative coupling rate and displayed in panel **b**. At the operation point for this work $\Phi_\mathrm{b}/\Phi_0 = 0.385$ (marked by dashed lines and star symbols), the rates we obtain are $g_{0\omega}/2\pi = 27.1\,\mathrm{kHz}$ and $g_{0\kappa}/2\pi = -48.8\,\mathrm{kHz}$.

we get in total

$$\Phi = \Phi_\mathrm{b} + (1-\alpha_L)\,\tilde{L}_\mathrm{loop}\frac{I_\mathrm{zpf}}{2} - \tilde{L}_\mathrm{loop} I_0 \sin\left(\pi\frac{\Phi}{\Phi_0}\right). \tag{S34}$$

Hence, the zero-point current is equivalent to an additional external flux

$$\Phi_\mathrm{zpf} = \tilde{L}_\mathrm{loop}\,(1-\alpha_L)\,\frac{I_\mathrm{zpf}}{2} \tag{S35}$$

$$= L_\mathrm{l} I_\mathrm{zpf} \tag{S36}$$

$$\approx 198.3\,\mu\Phi_0. \tag{S37}$$

In general Φ_zpf is also flux-dependent due to I_zpf being dependent on Ω_0 and L_LF, cf. Supplementary Fig. 7, but in our case it is nearly constant since the LF mode is so weakly dependent on flux.

Finally, we plug everything into the equations for $g_{0\omega}$ and $g_{0\kappa}$ and show both parameters as a function of Φ_b in Supplementary Fig. 8. Again, the relevant operation point $\Phi_\mathrm{b}/\Phi_0 = 0.385$ is marked with vertical dashed lines and star symbols, and the corresponding single-photon coupling rates are $g_{0\omega} = 2\pi\times 27.1\,\mathrm{kHz}$ and $g_{0\kappa} = 2\pi\times -48.8\,\mathrm{kHz}$.

In general it is important to distinguish between internal-dissipative and external-dissipative coupling, since they can lead to qualitatively and quantitatively different effects. From the fits in Supplementary Fig. 6 one would expect now that $g_{0\kappa_\mathrm{ext}}/g_{0\kappa_\mathrm{int}} \approx 10^{-2}$ or $g_{0\kappa_\mathrm{ext}} \approx 2\pi\times -0.55\,\mathrm{kHz}$, which despite its smallness would not be negligible for large pump-cavity detunings,

cf. Ref. [10] and Supplementary Note 6 D. However, based on our above conclusion, that the seeming flux-tuning of κ_{ext} is an artifact, we estimate the actual external-dissipative coupling rate to be only $g_{\kappa_{\mathrm{ext}}} = 2\pi \times 35.3\,\mathrm{Hz}$, i.e., $g_{0\kappa_{\mathrm{ext}}}/g_{0\kappa_{\mathrm{int}}} = -7.2 \times 10^{-4}$, using the equation $\kappa_{\mathrm{ext}} = \omega_0^2 Z_0 C_c^2 / C_{\mathrm{tot}}$, from which we find

$$g_{0\kappa_{\mathrm{ext}}} = \frac{\partial \kappa_{\mathrm{ext}}}{\partial \omega_0} g_{0\omega} \tag{S38}$$

$$= \frac{2\omega_0 Z_0 C_c^2}{C_{\mathrm{tot}}} g_{0\omega}. \tag{S39}$$

$$= 1.3 \times 10^{-3} g_{0\omega}. \tag{S40}$$

Such a small $g_{0\kappa_{\mathrm{ext}}}$ will not have measurable consequences within our experimental accuracy and can be safely neglected. The approach to completely neglect $g_{0\kappa_{\mathrm{ext}}}$ is backed up by our results being in good agreement with $g_{0\kappa} = g_{0\kappa_{\mathrm{int}}}$ and $g_{0\kappa_{\mathrm{ext}}} = 0$, in particular for large detunings between pump and HF mode and low pump photon numbers, where the impact of an external-dissipative coupling would be largest. For an additional discussion on the potential impact of an external-dissipative contribution to the results of this manuscript, see Supplementary Note 6 D.

4. SUPPLEMENTARY NOTE 4: THEORETICAL RESPONSE OF THE INDIVIDUAL CIRCUITS

A. The low-frequency circuit – Complete equation of motion

We model the dynamics of the low-frequency resonator in the low-power regime with the equation of motion (EOM)

$$\dot{\hat{b}} = \mathrm{i}\left(\Omega_0 + \mathcal{K}_{\mathrm{LF}} \hat{b}^\dagger \hat{b}\right)\hat{b} - \frac{\Gamma_0}{2}\hat{b} + \mathrm{i}\sqrt{\Gamma_{\mathrm{ext}}}\hat{b}_{\mathrm{in}} \tag{S41}$$

where $\hat{b}$ is the annihilation operator for quanta in the LF mode, Ω_0 is the LF resonance frequency, $\mathcal{K}_{\mathrm{LF}}$ is the LF mode Kerr anharmonicity, Γ_0 is the total LF damping rate, Γ_{ext} is the external damping rate and $\hat{b}_{\mathrm{in}}$ is the input field. Note that in all equations of this work, which are relevant for the analysis and interpretation of the results, we drop the stochastic Langevin input term describing the coupling of the circuits to their thermal baths, since those are not relevant to first order for the experiments we perform.

B. The low-frequency circuit – Single-tone response in the linear regime

For the vast majority of cases in this work, except for a few data points in the dynamical backaction analysis (cf. Supplementary Notes 7 C and 8 B), the LF Kerr anharmonicity is negligible and we will usually work with $\mathcal{K}_{\mathrm{LF}} = 0$. We can solve the remaining EOM by the method of Fourier transform and obtain

$$\hat{b}(\Omega) = \frac{\mathrm{i}\sqrt{\Gamma_{\mathrm{ext}}}}{\frac{\Gamma_0}{2} + \mathrm{i}(\Omega - \Omega_0)}\hat{b}_{\mathrm{in}}(\Omega). \tag{S42}$$

We note that in most instances, we will omit the explicit frequency-dependence and use for simplicity the same operators/variables for frequency-domain and time-domain. Which one it is in each instance, should be clear from the context. For the output field we have the input-output relation

$$\hat{b}_{\mathrm{out}} = \hat{b}_{\mathrm{in}} + \mathrm{i}\sqrt{\Gamma_{\mathrm{ext}}}\hat{b} \tag{S43}$$

which leads to the reflection coefficient

$$S_{11} = \frac{\langle \hat{b}_{\mathrm{out}} \rangle}{\langle \hat{b}_{\mathrm{in}} \rangle} \tag{S44}$$

$$= 1 - \frac{\Gamma_{\mathrm{ext}}}{\frac{\Gamma_0}{2} + \mathrm{i}(\Omega - \Omega_0)}. \tag{S45}$$

C. The low-frequency circuit – Single-tone response in the nonlinear regime

As mentioned before, this case is only relevant for few LF S_{11} traces during the experiment on dynamical backaction and close to the instability, cf. Supplementary Note 8 B. Since $\mathcal{K}_{\mathrm{LF}}$ is very small, we can treat the situation completely classically and set $\hat{b}_{\mathrm{in}} = \beta_{\mathrm{in}} e^{\mathrm{i}\phi_\beta}$ and $\hat{b} = \beta$ to get to

$$\dot{\beta} = \mathrm{i}\left(\Omega_0 + \mathcal{K}_{\mathrm{LF}}|\beta|^2\right)\beta - \frac{\Gamma_0}{2}\beta + \mathrm{i}\sqrt{\Gamma_{\mathrm{ext}}}\beta_{\mathrm{in}} e^{\mathrm{i}\phi_\beta} \tag{S46}$$

which is normalized such that $|\beta|^2 = n_\beta$ is the probe photon number in the LF mode and $|\beta_{\rm in}|^2 = P_\beta/\hbar\Omega = n_{{\rm in},\beta}$ is the LF input photon flux with on-chip LF probe power P_β. Both β and $\beta_{\rm in}$ shall be real-valued and the phase lag between them is encoded in ϕ_β.

Using again Fourier transformation and the detuning between LF probe and LF resonance $\Delta_\beta = \Omega - \Omega_0$, we find

$$\mathrm{i}\left[\Delta_\beta - \mathcal{K}_{\rm LF} n_\beta\right]\beta + \frac{\Gamma_0}{2}\beta = \mathrm{i}\sqrt{\Gamma_{\rm ext}}\beta_{\rm in}e^{\mathrm{i}\phi_\beta} \tag{S47}$$

or magnitude-squared

$$\left|\Delta_\beta - \mathcal{K}_{\rm LF} n_\beta\right|^2 n_\beta + \frac{\Gamma_0^2}{4} n_\beta = \Gamma_{\rm ext} n_{{\rm in},\beta}. \tag{S48}$$

The latter is a third-order polynomial and can be solved numerically to find n_β or $\beta = \sqrt{n_\beta}$, and all we need in addition to find the reflection

$$S_{11} = 1 + \mathrm{i}\sqrt{\Gamma_{\rm ext}}\frac{\beta}{\beta_{\rm in}}\mathrm{e}^{-\mathrm{i}\phi_\beta} \tag{S49}$$

is the phase ϕ_β, which can be obtained via

$$\phi_\beta = \mathrm{atan2}\left(-\frac{\Gamma_0}{2}, \Delta_\beta - \mathcal{K}_{\rm LF} n_\beta\right). \tag{S50}$$

D. The high-frequency circuit – Complete equation of motion

The EOM for the high-frequency circuit is similar to the one of the LF circuit, but in addition to the HF Kerr nonlinearity $\mathcal{K}$ induced by the nano-constrictions we include a nonlinear damping up to third order using κ_1, κ_2 amd κ_3 and get [7, 11]

$$\dot{\hat{a}} = \mathrm{i}\left(\omega_0 + \mathcal{K}\hat{a}^\dagger\hat{a}\right)\hat{a} - \frac{\kappa_0 + \kappa_1\hat{a}^\dagger\hat{a} + \kappa_2\left(\hat{a}^\dagger\hat{a}\right)^2 + \kappa_3\left(\hat{a}^\dagger\hat{a}\right)^3}{2}\hat{a} + \mathrm{i}\sqrt{\kappa_{\rm ext}}\hat{a}_{\rm in} \tag{S51}$$

where ω_0 is the low-excitation resonance frequency, κ_0 is the total low-excitation damping rate, $\kappa_{\rm ext}$ is the external damping rate and $\hat{a}_{\rm in}$ is the input field.

E. The high-frequency circuit – Single-tone response in the linear regime

In the low-power single-tone regime, we set $\mathcal{K} = \kappa_1 = \kappa_2 = \kappa_3 = 0$ and get

$$\dot{\hat{a}} = \mathrm{i}\omega_0\hat{a} - \frac{\kappa_0}{2}\hat{a} + \mathrm{i}\sqrt{\kappa_{\rm ext}}\hat{a}_{\rm in} \tag{S52}$$

which – in analogy to the low-frequency circuit – can be solved by Fourier transform to get

$$\hat{a} = \frac{\mathrm{i}\sqrt{\kappa_{\rm ext}}}{\frac{\kappa_0}{2} + \mathrm{i}\left(\omega - \omega_0\right)}\hat{a}_{\rm in} \tag{S53}$$

and to finally obtain the reflection

$$S_{11} = 1 - \frac{\kappa_{\rm ext}}{\frac{\kappa_0}{2} + \mathrm{i}\left(\omega - \omega_0\right)}. \tag{S54}$$

F. The high-frequency circuit – The linearized two-tone response

In the PPIT experiments we apply a strong pump tone to the HF cavity and additionally a weak, near-resonant probe tone. For this case, we linearize the system by assuming input and intracavity field to consist of a classical, coherent amplitude and a fluctuation field (e.g. a weak probe tone), i.e.,

$$\hat{a}_{\rm in} = \left[\alpha_{\rm in}\mathrm{e}^{\mathrm{i}\phi_{\rm p}} + \hat{c}_{\rm in}\right]\mathrm{e}^{\mathrm{i}\omega_{\rm p}t} \tag{S55}$$

$$\hat{a} = \left[\alpha_0 + \hat{c}\right]\mathrm{e}^{\mathrm{i}\omega_{\rm p}t} \tag{S56}$$

with real-valued and time-independent $\alpha_{\rm in}$ and α_0, complex-valued and time-dependent $\hat{c}_{\rm in}$ and $\hat{c}$, and with the pump frequency $\omega_{\rm p}$. A possible (frequency-dependent) phase lag between $\alpha_{\rm in}$ and α_0 is included via $\phi_{\rm p}$. Plugging the ansatz into the EOM and omitting afterwards the $e^{i\omega_{\rm p}t}$ factor (rotating frame) as well as nonlinear terms in $\hat{c}$, we find

$$i\omega_{\rm p}\alpha_0 + i\omega_{\rm p}\hat{c} + \dot{\hat{c}} = i\left(\omega_0 + \mathcal{K}\alpha_0^2\right)\alpha_0 - \frac{\kappa_0 + \kappa_1\alpha_0^2 + \kappa_2\alpha_0^4 + \kappa_3\alpha_0^6}{2}\alpha_0 + i\sqrt{\kappa_{\rm ext}}\alpha_{\rm in}e^{i\phi_{\rm p}} \tag{S57}$$

$$+ i\left(\omega_0 + 2\mathcal{K}\alpha_0^2\right)\hat{c} - \frac{\kappa_0 + 2\kappa_1\alpha_0^2 + 3\kappa_3\alpha_0^4 + 4\kappa_2\alpha_0^6}{2}\hat{c} + i\sqrt{\kappa_{\rm ext}}\hat{c}_{\rm in} \tag{S58}$$

$$+ i\mathcal{K}\alpha_0^2\hat{c}^\dagger - \frac{\kappa_1\alpha_0^2 + 2\kappa_2\alpha_0^4 + 3\kappa_3\alpha_0^6}{2}\hat{c}^\dagger. \tag{S59}$$

The first line on the right hand side (RHS) describes the steady-state terms of the pumped circuit with respect to the pump, the second line are the dynamic terms for $\hat{c}$ and the third line corresponds to amplification, squeezing and four-wave-mixing. We split the equation into steady-state constant parts

$$i\left[\Delta_{\rm p} - \mathcal{K}n_{\rm c}\right]\alpha_0 + \frac{\kappa_{\rm eff}}{2}\alpha_0 = i\sqrt{\kappa_{\rm ext}}\alpha_{\rm in}e^{i\phi_{\rm p}} \tag{S60}$$

and dynamical parts

$$\dot{\hat{c}} = -i\left[\Delta_{\rm p} - 2\mathcal{K}n_{\rm c}\right]\hat{c} - \frac{\kappa_0'}{2}\hat{c} + \left[i\mathcal{K} - \frac{\kappa_{\rm nl}}{2}\right]n_{\rm c}\hat{c}^\dagger + i\sqrt{\kappa_{\rm ext}}\hat{c}_{\rm in} \tag{S61}$$

using the detuning between the unpumped HF cavity and the pump $\Delta_{\rm p} = \omega_{\rm p} - \omega_0$, the intracavity pump photon number $n_{\rm c} = \alpha_0^2$, and the linewidths

$$\kappa_0' = \kappa_0 + 2\kappa_1 n_{\rm c} + 3\kappa_2 n_{\rm c}^2 + 4\kappa_3 n_{\rm c}^3 \tag{S62}$$

$$\kappa_{\rm eff} = \kappa_0 + \kappa_1 n_{\rm c} + \kappa_2 n_{\rm c}^2 + \kappa_3 n_{\rm c}^3 \tag{S63}$$

$$\kappa_{\rm nl} = \kappa_1 + 2\kappa_2 n_{\rm c} + 3\kappa_3 n_{\rm c}^2. \tag{S64}$$

Although not relevant for the further theory, note that $\kappa_0' = \kappa_{\rm eff} + \kappa_{\rm nl}n_{\rm c}$. We multiply Eq. (S60) by its complex conjugate and find the characteristic polynomial of a strongly driven, weakly-nonlinear Kerr oscillator with up to third order nonlinear damping

$$\left[\left(\Delta_{\rm p} - \mathcal{K}n_{\rm c}\right)^2 + \frac{\kappa_{\rm eff}^2}{4}\right]n_{\rm c} - \kappa_{\rm ext}n_{\rm in} = 0 \tag{S65}$$

where $n_{\rm in} = \alpha_{\rm in}^2$ is the input flux of pump photons. This seventh-order polynomial, which in principle can be solved numerically to find the stable solution(s) for the photon number $n_{\rm c}$, is also given in its expanded form in the main manuscript Methods section as Eq. (27). To get the complete solution $\alpha_0 e^{i\phi_{\rm p}}$, we can also calculate the phase between pump and intracircuit field as

$$\phi_{\rm p} = \operatorname{atan2}\left(-\frac{\kappa_{\rm eff}}{2}, \Delta_{\rm p} - \mathcal{K}n_{\rm c}\right), \tag{S66}$$

although this phase is not important for the probe response as long as pump and probe are at considerably different frequencies.

To calculate the probe response, we additionally need the EOM for $\hat{c}^\dagger$, which is

$$\dot{\hat{c}}^\dagger = i\left[\Delta_{\rm p} - 2\mathcal{K}n_{\rm c}\right]\hat{c}^\dagger - \frac{\kappa_0'}{2}\hat{c}^\dagger - \left[i\mathcal{K} + \frac{\kappa_{\rm nl}}{2}\right]n_{\rm c}\hat{c} - i\sqrt{\kappa_{\rm ext}}\hat{c}_{\rm in}^\dagger. \tag{S67}$$

We Fourier transform both Eq. (S61) and Eq. (S67) and get

$$\frac{\hat{c}(\Omega)}{\chi_{\rm p}(\Omega)} = \left[i\mathcal{K} - \frac{\kappa_{\rm nl}}{2}\right]n_{\rm c}\hat{c}^\dagger(-\Omega) + i\sqrt{\kappa_{\rm ext}}\hat{c}_{\rm in}(\Omega) \tag{S68}$$

$$\frac{\hat{c}^\dagger(-\Omega)}{\chi_{\rm p}^*(-\Omega)} = -\left[i\mathcal{K} + \frac{\kappa_{\rm nl}}{2}\right]n_{\rm c}\hat{c}(\Omega) - i\sqrt{\kappa_{\rm ext}}\hat{c}_{\rm in}^\dagger(-\Omega) \tag{S69}$$

where

$$\chi_{\rm p}(\Omega) = \frac{1}{\frac{\kappa_0'}{2} + i\left(\Delta_{\rm p} - 2\mathcal{K}n_{\rm c} + \Omega\right)}, \tag{S70}$$

and $\Omega = \omega - \omega_\mathrm{p}$ the frequency relative to the pump. We drop the input term in the equation for $\hat{c}^\dagger$ at this point, since we only detect the Fourier component of $\hat{c}$ at Ω with the VNA. Next, we define $\overline{\chi}_\mathrm{p} = \chi_\mathrm{p}^*(-\Omega)$, we omit the explicit statement of $(\pm\Omega)$ and combine the two equations to

$$\frac{\hat{c}}{\chi_\mathrm{g}} = \mathrm{i}\sqrt{\kappa_\mathrm{ext}}\hat{c}_\mathrm{in} \tag{S71}$$

with

$$\chi_\mathrm{g} = \frac{\chi_\mathrm{p}}{1 - \left[\mathcal{K}^2 + \frac{\kappa_\mathrm{nl}^2}{4}\right] n_\mathrm{c}^2 \chi_\mathrm{p}\overline{\chi}_\mathrm{p}}. \tag{S72}$$

The probe reflection follows as

$$S_{11} = 1 - \kappa_\mathrm{ext}\chi_\mathrm{g}. \tag{S73}$$

G. The pumped Kerr eigenmodes

We can find the solutions to the pumped susceptibility χ_g by the condition $\chi_\mathrm{g}^{-1} = 0$, which is equivalent to

$$1 - \left[\mathcal{K}^2 + \frac{\kappa_\mathrm{nl}^2}{4}\right] n_\mathrm{c}^2 \chi_\mathrm{p}\overline{\chi}_\mathrm{p} = 0 \tag{S74}$$

and which is solved by the complex frequencies

$$\tilde{\omega}_{1/2} = \omega_\mathrm{p} + \mathrm{i}\frac{\kappa_0'}{2} \pm \sqrt{\left(\Delta_\mathrm{p} - \mathcal{K}n_\mathrm{c}\right)\left(\Delta_\mathrm{p} - 3\mathcal{K}n_\mathrm{c}\right) - \frac{\kappa_\mathrm{nl}^2 n_\mathrm{c}^2}{4}}. \tag{S75}$$

The real parts of this solution correspond to the resonance frequencies and the imaginary parts to half the linewidths. We always work in a regime where only one of the two modes plays a significant role (moderate pumping with a far-red-detuned pump tone), whose solution is

$$\tilde{\omega}_0 = \omega_\mathrm{p} + \mathrm{i}\frac{\kappa_0'}{2} + \sqrt{\left(\Delta_\mathrm{p} - \mathcal{K}n_\mathrm{c}\right)\left(\Delta_\mathrm{p} - 3\mathcal{K}n_\mathrm{c}\right) - \frac{\kappa_\mathrm{nl}^2 n_\mathrm{c}^2}{4}}. \tag{S76}$$

This means the new probe-mode is broadened to a linewidth $\kappa_0' = \kappa_0 + 2\kappa_1 n_\mathrm{c} + 3\kappa_2 n_\mathrm{c}^2 + 4\kappa_3 n_\mathrm{c}^3$ and ac-Stark-shifted by

$$\delta\omega_0 = \Delta_\mathrm{p} + \sqrt{\left(\Delta_\mathrm{p} - \mathcal{K}n_\mathrm{c}\right)\left(\Delta_\mathrm{p} - 3\mathcal{K}n_\mathrm{c}\right) - \frac{\kappa_\mathrm{nl}^2 n_\mathrm{c}^2}{4}}. \tag{S77}$$

We call the pumped resonance frequency $\omega_0' = \omega_0 + \delta\omega_0$ and get in good approximation for all our experiments

$$\chi_\mathrm{g} \approx \frac{1}{\frac{\kappa_0'}{2} + \mathrm{i}(\omega - \omega_0')}. \tag{S78}$$

Note that in general one would also have to consider the probe gain $\mathcal{G}$ due to parametric amplification [12, 13], but we always work in a regime where $\mathcal{G} \approx 1$.

To see the latter relation and $\mathcal{G} \approx 1$, we write χ_g as

$$\chi_\mathrm{g} = \frac{\chi_1\chi_2}{\overline{\chi}_\mathrm{p}} \tag{S79}$$

with

$$\chi_1 = \frac{1}{\frac{\kappa_0'}{2} + \mathrm{i}(\omega - \omega_0')}, \quad \chi_2 = \frac{1}{\frac{\kappa_0'}{2} + \mathrm{i}(\omega - \omega_1')} \tag{S80}$$

where

$$\omega_0' = \omega_\mathrm{p} + \sqrt{\left(\Delta_\mathrm{p} - \mathcal{K}n_\mathrm{c}\right)\left(\Delta_\mathrm{p} - 3\mathcal{K}n_\mathrm{c}\right) - \frac{\kappa_\mathrm{nl}^2 n_\mathrm{c}^2}{4}} \tag{S81}$$

$$\omega_1' = \omega_\mathrm{p} - \sqrt{\left(\Delta_\mathrm{p} - \mathcal{K}n_\mathrm{c}\right)\left(\Delta_\mathrm{p} - 3\mathcal{K}n_\mathrm{c}\right) - \frac{\kappa_\mathrm{nl}^2 n_\mathrm{c}^2}{4}}. \tag{S82}$$

It can also easily be confirmed, that

$$\chi_1\chi_2 = \frac{\chi_1 + \chi_2}{\kappa_0' + 2i\Omega}. \tag{S83}$$

With all this, we can write

$$\chi_g = \mathcal{G}(\chi_1 + \chi_2) \tag{S84}$$

with the frequency-dependent gain

$$\mathcal{G} = \frac{\frac{\kappa_0'}{2} - i\left(\Delta_p - 2\mathcal{K}n_c - \Omega\right)}{\kappa_0' + 2i\Omega}. \tag{S85}$$

For the parameter range in the experiment presented in this paper (i.e. a red-sideband pump with $\Delta_p \approx -\Omega_0 + 2\mathcal{K}n_c$ and probe frequencies around ω_0', equivalent to $\Omega \approx \Omega_0$), we find

$$\begin{aligned}\mathcal{G} &\approx \frac{\frac{\kappa_0'}{2} + 2i\Omega_0}{\kappa_0' + 2i\Omega_0} \\ &\approx 1 \end{aligned} \tag{S86}$$

$$\chi_2 \approx 0 \tag{S87}$$

and hence

$$\mathcal{G}(\chi_1 + \chi_2) \approx \chi_1. \tag{S88}$$

H. Intracircuit pump photon number

Due to the unknown and strongly frequency-dependent attenuation in our setup, originating from a pump-span covering more than 300 MHz, we extract n_c from the HF cavity frequency shift due to its Kerr anharmonicity in combination with the photon-pressure coupling strength. In the preceding Sec. 4 G, we derived the expression Eq. (S77) for the frequency shift

$$\delta\omega_0 = \Delta_p + \sqrt{\left(\Delta_p - \mathcal{K}n_c\right)\left(\Delta_p - 3\mathcal{K}n_c\right) - \frac{\kappa_{nl}^2 n_c^2}{4}}. \tag{S89}$$

However, in contrast to the case of only first order nonlinear damping [7, 8] the quantity $\kappa_{nl}n_c = \kappa_1 n_c + 2\kappa_2 n_c^2 + 3\kappa_3 n_c^3$ is not experimentally accessible with ease. What we do have access to though is

$$2\kappa_1 n_c + 3\kappa_2 n_c^2 + 4\kappa_3 n_c^3 = \kappa_0' - \kappa_0 \geq \kappa_{nl} n_c \quad \text{for} \quad \kappa_1, \kappa_2, \kappa_3 \geq 0 \tag{S90}$$

and so if we write the resonance frequency shift as

$$\delta\omega_0 = \Delta_p + |\Delta_p|\sqrt{1 - \frac{4\mathcal{K}n_c}{\Delta_p} + \frac{3\mathcal{K}^2 n_c^2}{\Delta_p^2} - \frac{\kappa_{nl}^2 n_c^2}{4\Delta_p^2}} \tag{S91}$$

and observe experimentally

$$\kappa_0' - \kappa_0 \ll 2|\Delta_p| \tag{S92}$$

we also know immediately that

$$\frac{\kappa_{nl}^2 n_c^2}{4\Delta_p^2} \leq \frac{\left(\kappa_0' - \kappa_0\right)^2}{4\Delta_p^2} \ll 1 \tag{S93}$$

and so we can omit it. Also the second-last term is small and we can write

$$\delta\omega_0 \approx \Delta_p + |\Delta_p|\sqrt{1 - \frac{4\mathcal{K}n_c}{\Delta_p}}, \tag{S94}$$

which for small shifts compared to the detuning $|\mathcal{K}n_c/\Delta_p| \ll 1$ and $\Delta_p < 0$ could be Taylor-approximated as

$$\delta\omega_0 \approx 2\mathcal{K}n_c, \tag{S95}$$

i.e., as the well-known relation. Nevertheless we work with Eq. (S94) here and rearrange it to

$$n_c = \frac{\delta\omega_0\left(2 - \delta\omega_0/\Delta_p\right)}{4\mathcal{K}}. \tag{S96}$$

There is another useful relation, which connects the on-chip pump power $P_p = n_{in}\hbar\omega_p$ to the intracircuit pump photon number n_c. To get there, we just rearrange the characteristic polynomial Eq. (S65) and find

$$n_c = \frac{4P_p}{\hbar\omega_p}\frac{\kappa_{ext}}{\kappa_{eff}^2 + 4\tilde{\Delta}_p^2}, \tag{S97}$$

where $\tilde{\Delta}_p = \Delta_p - \mathcal{K}n_c$.

5. SUPPLEMENTARY NOTE 5: THEORETICAL MODEL OF THE COUPLED CIRCUITS

A. Hamiltonian and equations of motion

We start our considerations with the Hamiltonian of the coupled circuits

$$\hat{H}/\hbar = \omega_0\hat{a}^\dagger\hat{a} + \Omega_0\hat{b}^\dagger\hat{b} - g_{0\omega}\hat{a}^\dagger\hat{a}\left(\hat{b} + \hat{b}^\dagger\right) + \frac{\mathcal{K}}{2}\left(\hat{a}^\dagger\hat{a}\right)^2 - \frac{g_{0\mathcal{K}}}{2}\left(\hat{a}^\dagger\hat{a}\right)^2\left(\hat{b} + \hat{b}^\dagger\right) + \mathcal{K}_c\hat{a}^\dagger\hat{a}\hat{b}^\dagger\hat{b} + \hat{H}_{bath}/\hbar \tag{S98}$$

which contains the self-Kerr term $\frac{\mathcal{K}}{2}\left(\hat{a}^\dagger\hat{a}\right)^2$ and a possible cross-Kerr term $\mathcal{K}_c\hat{a}^\dagger\hat{a}\hat{b}^\dagger\hat{b}$. The two single-photon coupling rates $g_{0\omega}$ and $g_{0\mathcal{K}}$ originate from the flux-dependence of ω_0 and $\mathcal{K}$ and are given by

$$g_{0\omega} = -\frac{\partial\omega_0}{\partial\Phi_b}\Phi_{zpf} \tag{S99}$$

$$g_{0\mathcal{K}} = -\frac{\partial\mathcal{K}}{\partial\Phi_b}\Phi_{zpf}. \tag{S100}$$

Furthermore, we will assume for the bath Hamiltonian that the internal HF linewidth is given by

$$\kappa_0 - g_{0\kappa}\left(\hat{b} + \hat{b}^\dagger\right) \quad \text{with} \quad g_{0\kappa} = -\frac{\partial\kappa_0}{\partial\Phi_b}\Phi_{zpf} \tag{S101}$$

the dissipative coupling rate, while $\kappa_{ext}, \Gamma_{ext}, \Gamma_{int}$ are constant. Later, we will introduce a phenomenological pump-power dependence of both κ_{int} and Γ_{int}, and for the former also the corresponding PP coupling terms. The bath Hamiltonian in RWA according to Ref. [14–17] is given by (we choose the sum notation for the bath modes here, but it is equivalent to the integral notation)

$$\hat{H}_{bath}/\hbar = \sum_n \omega_n\hat{e}_n^\dagger\hat{e}_n + \sum_m \omega_m\hat{i}_m^\dagger\hat{i}_m - i\sqrt{\frac{\kappa_{ext}}{2\pi\rho_{ext}}}\sum_n\left(\hat{a}^\dagger\hat{e}_n - \hat{e}_n^\dagger\hat{a}\right) - i\sqrt{\frac{\kappa_{int}}{2\pi\rho_{int}}}\sum_m\left(\hat{a}^\dagger\hat{i}_m - \hat{i}_m^\dagger\hat{a}\right) + \hat{H}_\Gamma/\hbar \tag{S102}$$

where the $\hat{e}$ and $\hat{i}$ operators are describing the external and internal HF cavity bath modes, ρ_{int} and ρ_{ext} are the frequency-independent bath density of states, and

$$\hat{H}_\Gamma/\hbar = \sum_l \Omega_l\hat{E}_l^\dagger\hat{E}_l + \sum_j \Omega_j\hat{I}_j^\dagger\hat{I}_j - i\sqrt{\frac{\Gamma_{ext}}{2\pi\tilde{\rho}_{ext}}}\sum_l\left(\hat{b}^\dagger\hat{E}_l - \hat{E}_l^\dagger\hat{b}\right) - i\sqrt{\frac{\Gamma_{int}}{2\pi\tilde{\rho}_{int}}}\sum_j\left(\hat{b}^\dagger\hat{I}_j - \hat{I}_j^\dagger\hat{b}\right) \tag{S103}$$

takes into account the bath and dissipation of the low-frequency circuit. To get a useful expression with the LF-flux-dependent

κ-terms, we calculate

$$\hat{H}_{\text{bath}} \approx \hat{H}_{\text{bath},0} + \frac{\partial \hat{H}_{\text{bath}}}{\partial\left(\hat{b} + \hat{b}^\dagger\right)}\left(\hat{b} + \hat{b}^\dagger\right) \tag{S104}$$

$$= \hat{H}_{\text{bath},0} + \frac{\partial \hat{H}_{\text{bath}}}{\partial \kappa_{\text{int}}}\frac{\partial \kappa_{\text{int}}}{\partial\left(\hat{b} + \hat{b}^\dagger\right)}\left(\hat{b} + \hat{b}^\dagger\right) \tag{S105}$$

$$= \hat{H}_{\text{bath},0} - \frac{\partial \hat{H}_{\text{bath}}}{\partial \kappa_{\text{int}}} g_{0\kappa}\left(\hat{b} + \hat{b}^\dagger\right) \tag{S106}$$

$$= \hat{H}_{\text{bath},0} + \mathrm{i}\frac{\hbar g_{0\kappa}}{2}\sqrt{\frac{1}{2\pi\rho_{\text{int}}\kappa_{\text{int}}}}\left(\hat{b} + \hat{b}^\dagger\right)\sum_m\left(\hat{a}^\dagger\hat{i}_m - \hat{i}_m^\dagger\hat{a}\right) \tag{S107}$$

since for our device $\partial\kappa_0/\partial\Phi_{\text{b}} \approx \partial\kappa_{\text{int}}/\partial\Phi_{\text{b}}$.

From here, we get the coupled EOMs as

$$\dot{\hat{e}}_n = \mathrm{i}\omega_n\hat{e}_n - \sqrt{\frac{\kappa_{\text{ext}}}{2\pi\rho_{\text{ext}}}}\hat{a} \tag{S108}$$

$$\dot{\hat{i}}_m = \mathrm{i}\omega_m\hat{i}_m - \sqrt{\frac{\kappa_{\text{int}}}{2\pi\rho_{\text{int}}}}\hat{a} + \frac{g_{0\kappa}}{2}\sqrt{\frac{1}{2\pi\rho_{\text{int}}\kappa_{\text{int}}}}\left(\hat{b} + \hat{b}^\dagger\right)\hat{a} \tag{S109}$$

$$\dot{\hat{E}}_l = \mathrm{i}\Omega_l\hat{E}_l - \sqrt{\frac{\Gamma_{\text{ext}}}{2\pi\tilde{\rho}_{\text{ext}}}}\hat{b} \tag{S110}$$

$$\dot{\hat{I}}_j = \mathrm{i}\Omega_j\hat{I}_j - \sqrt{\frac{\Gamma_{\text{int}}}{2\pi\tilde{\rho}_{\text{int}}}}\hat{b} \tag{S111}$$

$$\begin{aligned}\dot{\hat{a}} &= \mathrm{i}\left[\omega_0 - g_{0\omega}\left(\hat{b} + \hat{b}^\dagger\right) + \mathcal{K}\left(\hat{a}^\dagger\hat{a} + \frac{1}{2}\right) - g_{0\mathcal{K}}\left(\hat{a}^\dagger\hat{a} + \frac{1}{2}\right)\left(\hat{b} + \hat{b}^\dagger\right) + \mathcal{K}_{\text{c}}\hat{b}^\dagger\hat{b}\right]\hat{a}\\ &\quad + \sqrt{\frac{\kappa_{\text{ext}}}{2\pi\rho_{\text{ext}}}}\sum_n\hat{e}_n + \sqrt{\frac{\kappa_{\text{int}}}{2\pi\rho_{\text{int}}}}\sum_m\hat{i}_m - \frac{g_{0\kappa}}{2}\sqrt{\frac{1}{2\pi\rho_{\text{int}}\kappa_{\text{int}}}}\left(\hat{b} + \hat{b}^\dagger\right)\sum_m\hat{i}_m\end{aligned} \tag{S112}$$

$$\dot{\hat{b}} = \mathrm{i}\left[\Omega_0 + \mathcal{K}_{\text{c}}\hat{a}^\dagger\hat{a}\right]\hat{b} - \mathrm{i}g_{0\omega}\hat{a}^\dagger\hat{a} - \mathrm{i}\frac{g_{0\mathcal{K}}}{2}\left(\hat{a}^\dagger\hat{a}\right)^2 + \sqrt{\frac{\Gamma_{\text{ext}}}{2\pi\tilde{\rho}_{\text{ext}}}}\sum_l\hat{E}_l + \sqrt{\frac{\Gamma_{\text{int}}}{2\pi\tilde{\rho}_{\text{int}}}}\sum_j\hat{I}_j - \frac{g_{0\kappa}}{2}\sqrt{\frac{1}{2\pi\rho_{\text{int}}\kappa_{\text{int}}}}\sum_m\left(\hat{a}^\dagger\hat{i}_m - \hat{i}_m^\dagger\hat{a}\right) \tag{S113}$$

and we solve the equations for the baths as

$$\hat{E}_l = \hat{E}_{l,0}\mathrm{e}^{\mathrm{i}\Omega_l(t-t_0)} - \sqrt{\frac{\Gamma_{\text{ext}}}{2\pi\tilde{\rho}_{\text{ext}}}}\int_{t_0}^{t}\mathrm{e}^{\mathrm{i}\Omega_l(t-t')}\hat{b}(t')dt' \tag{S114}$$

$$\hat{I}_j = \hat{I}_{j,0}\mathrm{e}^{\mathrm{i}\Omega_j(t-t_0)} - \sqrt{\frac{\Gamma_{\text{int}}}{2\pi\tilde{\rho}_{\text{int}}}}\int_{t_0}^{t}\mathrm{e}^{\mathrm{i}\Omega_j(t-t')}\hat{b}(t')dt' \tag{S115}$$

$$\hat{e}_n = \hat{e}_{n,0}\mathrm{e}^{\mathrm{i}\omega_n(t-t_0)} - \sqrt{\frac{\kappa_{\text{ext}}}{2\pi\rho_{\text{ext}}}}\int_{t_0}^{t}\mathrm{e}^{\mathrm{i}\omega_n(t-t')}\hat{a}(t')dt' \tag{S116}$$

$$\hat{i}_m = \hat{i}_{m,0}\mathrm{e}^{\mathrm{i}\omega_m(t-t_0)} - \sqrt{\frac{\kappa_{\text{int}}}{2\pi\rho_{\text{int}}}}\int_{t_0}^{t}\mathrm{e}^{\mathrm{i}\omega_m(t-t')}\hat{a}(t')\left[1 - \frac{g_{0\kappa}}{2\kappa_{\text{int}}}\left(\hat{b}(t') + \hat{b}^\dagger(t')\right)\right]dt' \tag{S117}$$

$$\hat{i}_m^\dagger = \hat{i}_{m,0}^\dagger\mathrm{e}^{-\mathrm{i}\omega_m(t-t_0)} - \sqrt{\frac{\kappa_{\text{int}}}{2\pi\rho_{\text{int}}}}\int_{t_0}^{t}\mathrm{e}^{-\mathrm{i}\omega_m(t-t')}\hat{a}^\dagger(t')\left[1 - \frac{g_{0\kappa}}{2\kappa_{\text{int}}}\left(\hat{b}(t') + \hat{b}^\dagger(t')\right)\right]dt'. \tag{S118}$$

Then we can write the input terms in the EOMs for $\hat{a}$ as

$$\sqrt{\frac{\kappa_{\text{ext}}}{2\pi\rho_{\text{ext}}}}\sum_n\hat{e}_n = \sqrt{\frac{\kappa_{\text{ext}}}{2\pi\rho_{\text{ext}}}}\sum_n\left[\hat{e}_{n,0}\mathrm{e}^{\mathrm{i}\omega_n(t-t_0)} - \sqrt{\frac{\kappa_{\text{ext}}}{2\pi\rho_{\text{ext}}}}\int_{t_0}^{t}\mathrm{e}^{\mathrm{i}\omega_n(t-t')}\hat{a}(t')dt'\right] \tag{S119}$$

$$= \sqrt{\frac{\kappa_{\text{ext}}}{2\pi\rho_{\text{ext}}}}\sum_n\hat{e}_{n,0}\mathrm{e}^{\mathrm{i}\omega_n(t-t_0)} - \frac{\kappa_{\text{ext}}}{2\pi\rho_{\text{ext}}}\sum_n\int_{t_0}^{t}\mathrm{e}^{\mathrm{i}\omega_n(t-t')}\hat{a}(t')dt' \tag{S120}$$

$$= \sqrt{\kappa_{\text{ext}}}\hat{a}_{\text{ext,in}} - \frac{\kappa_{\text{ext}}}{2}\hat{a} \tag{S121}$$

$$\sqrt{\frac{\kappa_{\rm int}}{2\pi\rho_{\rm int}}}\sum_m \hat{i}_m = \sqrt{\kappa_{\rm int}}\hat{a}_{\rm int,in} - \frac{\kappa_{\rm int}}{2}\hat{a} + \frac{g_{0\kappa}}{4}\hat{a}\left(\hat{b}+\hat{b}^\dagger\right) \tag{S122}$$

$$\frac{g_{0\kappa}}{2}\sqrt{\frac{1}{2\pi\rho_{\rm int}\kappa_{\rm int}}}\left(\hat{b}+\hat{b}^\dagger\right)\sum_m \hat{i}_m = \frac{g_{0\kappa}}{2\sqrt{\kappa_{\rm int}}}\hat{a}_{\rm int,in}\left(\hat{b}+\hat{b}^\dagger\right) - \frac{g_{0\kappa}}{4}\hat{a}\left(\hat{b}+\hat{b}^\dagger\right) + \frac{g_{0\kappa}^2}{8\kappa_{\rm int}}\hat{a}\left(\hat{b}+\hat{b}^\dagger\right)^2. \tag{S123}$$

On the other hand we get for the input terms of the LF resonator

$$\sqrt{\frac{\Gamma_{\rm ext}}{2\pi\tilde{\rho}_{\rm ext}}}\sum_l \hat{E}_l = \sqrt{\Gamma_{\rm ext}}\hat{b}_{\rm ext,in} - \frac{\Gamma_{\rm ext}}{2}\hat{b} \tag{S124}$$

$$\sqrt{\frac{\Gamma_{\rm int}}{2\pi\tilde{\rho}_{\rm int}}}\sum_j \hat{I}_j = \sqrt{\Gamma_{\rm int}}\hat{b}_{\rm int,in} - \frac{\Gamma_{\rm int}}{2}\hat{b} \tag{S125}$$

$$\begin{aligned}
\frac{g_{0\kappa}}{2}\sqrt{\frac{1}{2\pi\rho_{\rm int}\kappa_{\rm int}}}\sum_m\left(\hat{a}^\dagger\hat{i}_m - \hat{i}_m^\dagger\hat{a}\right) &= \frac{g_{0\kappa}}{2\kappa_{\rm int}}\hat{a}^\dagger\sqrt{\frac{\kappa_{\rm int}}{2\pi\rho_{\rm int}}}\sum_m\hat{i}_m - \frac{g_{0\kappa}}{2\kappa_{\rm int}}\hat{a}\sqrt{\frac{\kappa_{\rm int}}{2\pi\rho_{\rm int}}}\sum_m\hat{i}_m^\dagger && \text{(S126)}\\
&= \frac{g_{0\kappa}}{2\kappa_{\rm int}}\hat{a}^\dagger\left[\sqrt{\kappa_{\rm int}}\hat{a}_{\rm int,in} - \frac{\kappa_{\rm int}}{2}\hat{a} + \frac{g_{0\kappa}}{4}\hat{a}\left(\hat{b}+\hat{b}^\dagger\right)\right] && \text{(S127)}\\
&\quad -\frac{g_{0\kappa}}{2\kappa_{\rm int}}\hat{a}\left[\sqrt{\kappa_{\rm int}}\hat{a}_{\rm int,in}^\dagger - \frac{\kappa_{\rm int}}{2}\hat{a}^\dagger + \frac{g_{0\kappa}}{4}\hat{a}^\dagger\left(\hat{b}+\hat{b}^\dagger\right)\right] && \text{(S128)}\\
&= \frac{g_{0\kappa}}{2\sqrt{\kappa_{\rm int}}}\left(\hat{a}^\dagger\hat{a}_{\rm int,in} - \hat{a}\hat{a}_{\rm int,in}^\dagger\right) + \frac{g_{0\kappa}}{4} - \frac{g_{0\kappa}^2}{8\kappa_{\rm int}}\left(\hat{b}+\hat{b}^\dagger\right). && \text{(S129)}
\end{aligned}$$

Combining all results leads to the set of EOMs

$$\begin{aligned}
\dot{\hat{a}} &= \mathrm{i}\left[\omega_0 - g_{0\omega}\left(\hat{b}+\hat{b}^\dagger\right) + \mathcal{K}\left(\hat{a}^\dagger\hat{a}+\frac{1}{2}\right) - g_{0\mathcal{K}}\left(\hat{a}^\dagger\hat{a}+\frac{1}{2}\right)\left(\hat{b}+\hat{b}^\dagger\right) + \mathcal{K}_{\rm c}\hat{b}^\dagger\hat{b}\right]\hat{a}\\
&\quad -\frac{\kappa_0}{2}\hat{a} + \frac{g_{0\kappa}}{2}\hat{a}\left(\hat{b}+\hat{b}^\dagger\right) - \frac{g_{0\kappa}^2}{8\kappa_{\rm int}}\hat{a}\left(\hat{b}+\hat{b}^\dagger\right)^2\\
&\quad +\sqrt{\kappa_{\rm ext}}\hat{a}_{\rm ext,in} + \sqrt{\kappa_{\rm int}}\hat{a}_{\rm int,in} - \frac{g_{0\kappa}}{2\sqrt{\kappa_{\rm int}}}\hat{a}_{\rm int,in}\left(\hat{b}+\hat{b}^\dagger\right) && \text{(S130)}\\
\dot{\hat{b}} &= \mathrm{i}\left[\Omega_0 + \mathcal{K}_{\rm c}\hat{a}^\dagger\hat{a}\right]\hat{b} - \mathrm{i}g_{0\omega}\hat{a}^\dagger\hat{a} - \mathrm{i}\frac{g_{0\mathcal{K}}}{2}\left(\hat{a}^\dagger\hat{a}\right)^2 - \frac{\Gamma_0}{2}\hat{b} - \frac{g_{0\kappa}}{4} + \frac{g_{0\kappa}^2}{8\kappa_{\rm int}}\left(\hat{b}+\hat{b}^\dagger\right)\\
&\quad +\sqrt{\Gamma_{\rm ext}}\hat{b}_{\rm ext,in} + \sqrt{\Gamma_{\rm int}}\hat{b}_{\rm int,in} - \frac{g_{0\kappa}}{2\sqrt{\kappa_{\rm int}}}\left(\hat{a}^\dagger\hat{a}_{\rm int,in} - \hat{a}\hat{a}_{\rm int,in}^\dagger\right). && \text{(S131)}
\end{aligned}$$

B. Dropping constants, small shifts, the quadratic term and input noise

For the coherent pump-probe experiments we are performing here, we will drop all noise input terms first since they are not relevant to first order, so the only input terms we keep are $\hat{a}_{\rm ext,in} = \mathrm{i}\hat{a}_{\rm in}$ and $\hat{b}_{\rm ext,in} = \mathrm{i}\hat{b}_{\rm in}$, which represent pump and probe signals. At the same time, we drop HF cavity quantum noise contributions (the $+1/2$ contributions in the parenthesis expressions, which are negligibly small in the thermal regime), higher order contributions in $\hat{b}$, $\hat{b}^\dagger$, the cross-Kerr term from the LF to the HF mode (the resulting frequency shift is negligible for the high-frequency, large-linewidth HF circuit of this work), and the small term $\propto g_{0\kappa}^2/8\kappa_{\rm int}$ in Eq. (S131). After all this we get

$$\dot{\hat{a}} = \mathrm{i}\left[\omega_0 - g_{0\omega}\left(\hat{b}+\hat{b}^\dagger\right) + \mathcal{K}\hat{a}^\dagger\hat{a} - g_{0\mathcal{K}}\hat{a}^\dagger\hat{a}\left(\hat{b}+\hat{b}^\dagger\right)\right]\hat{a} - \frac{\kappa_0}{2}\hat{a} + \frac{g_{0\kappa}}{2}\hat{a}\left(\hat{b}+\hat{b}^\dagger\right) + \mathrm{i}\sqrt{\kappa_{\rm ext}}\hat{a}_{\rm in} \tag{S132}$$

$$\dot{\hat{b}} = \mathrm{i}\left[\Omega_0 + \mathcal{K}_{\rm c}\hat{a}^\dagger\hat{a}\right]\hat{b} - \mathrm{i}g_{0\omega}\hat{a}^\dagger\hat{a} - \mathrm{i}\frac{g_{0\mathcal{K}}}{2}\left(\hat{a}^\dagger\hat{a}\right)^2 - \frac{\Gamma_0}{2}\hat{b} + \mathrm{i}\sqrt{\Gamma_{\rm ext}}\hat{b}_{\rm in}. \tag{S133}$$

C. Adding nonlinear damping

Finally we add up to third order nonlinear damping and cross-damping phenomenologically, cf. also Sec. 4, and get

$$\dot{\hat{a}} = \mathrm{i}\left[\omega_0 - g_{0\omega}\left(\hat{b}+\hat{b}^\dagger\right) + \mathcal{K}\hat{a}^\dagger\hat{a} - g_{0\mathcal{K}}\hat{a}^\dagger\hat{a}\left(\hat{b}+\hat{b}^\dagger\right)\right]\hat{a} + \mathrm{i}\sqrt{\kappa_{\mathrm{ext}}}\hat{a}_{\mathrm{in}} \tag{S134}$$

$$-\frac{1}{2}\left[\kappa_0 - g_{0\kappa}\left(\hat{b}+\hat{b}^\dagger\right) + \kappa_1\hat{a}^\dagger\hat{a} - g_{\mathrm{nl1}}\hat{a}^\dagger\hat{a}\left(\hat{b}+\hat{b}^\dagger\right) + \kappa_2\left(\hat{a}^\dagger\hat{a}\right)^2 - g_{\mathrm{nl2}}\left(\hat{a}^\dagger\hat{a}\right)^2\left(\hat{b}+\hat{b}^\dagger\right) + \kappa_3\left(\hat{a}^\dagger\hat{a}\right)^3 - g_{\mathrm{nl3}}\left(\hat{a}^\dagger\hat{a}\right)^3\left(\hat{b}+\hat{b}^\dagger\right)\right]\hat{a}$$

$$\dot{\hat{b}} = \mathrm{i}\left[\Omega_0 + \mathcal{K}_{\mathrm{c}}\hat{a}^\dagger\hat{a}\right]\hat{b} - \mathrm{i}g_{0\omega}\hat{a}^\dagger\hat{a} - \mathrm{i}\frac{g_{0\mathcal{K}}}{2}\left(\hat{a}^\dagger\hat{a}\right)^2 - \frac{\Gamma_0 + \kappa_{\mathrm{c}}\hat{a}^\dagger\hat{a}}{2}\hat{b} + \mathrm{i}\sqrt{\Gamma_{\mathrm{ext}}}\hat{b}_{\mathrm{in}}. \tag{S135}$$

As a reminder, the photon-pressure single-photon coupling rates here are defined via

$$g_{0\omega} = -\frac{\partial\omega_0}{\partial\Phi_{\mathrm{b}}}\Phi_{\mathrm{zpf}} \tag{S136}$$

$$g_{0\mathcal{K}} = -\frac{\partial\mathcal{K}}{\partial\Phi_{\mathrm{b}}}\Phi_{\mathrm{zpf}} \tag{S137}$$

$$g_{0\kappa} = -\frac{\partial\kappa_0}{\partial\Phi_{\mathrm{b}}}\Phi_{\mathrm{zpf}} \tag{S138}$$

$$g_{\mathrm{nl1}} = -\frac{\partial\kappa_1}{\partial\Phi_{\mathrm{b}}}\Phi_{\mathrm{zpf}} \tag{S139}$$

$$g_{\mathrm{nl2}} = -\frac{\partial\kappa_2}{\partial\Phi_{\mathrm{b}}}\Phi_{\mathrm{zpf}} \tag{S140}$$

$$g_{\mathrm{nl3}} = -\frac{\partial\kappa_3}{\partial\Phi_{\mathrm{b}}}\Phi_{\mathrm{zpf}} \tag{S141}$$

with the SQUID bias flux Φ_{b} and the zero-point-fluctuation flux of the LF mode coupling into the SQUID Φ_{zpf}. In principle also the cross-Kerr and cross-damping constants will depend on magnetic flux and lead to a coupling term, but since the terms themselves are very small here, we neglect their coupling contributions.

D. Low-pump-power regime – Equations of motion

For low pump powers, we can set all nonlinearities to zero, i.e., $\mathcal{K}, \mathcal{K}_{\mathrm{c}}, \kappa_1, \kappa_2, \kappa_3, \kappa_{\mathrm{c}} = 0$; we also drop the corresponding coupling terms. Then we get

$$\dot{\hat{a}} = \left[\mathrm{i}\omega_0 - \frac{\kappa_0}{2}\right]\hat{a} - \mathrm{i}\left[g_{0\omega} + \mathrm{i}\frac{g_{0\kappa}}{2}\right]\left(\hat{b}+\hat{b}^\dagger\right)\hat{a} + \mathrm{i}\sqrt{\kappa_{\mathrm{ext}}}\hat{a}_{\mathrm{in}} \tag{S142}$$

$$\dot{\hat{b}} = \left[\mathrm{i}\Omega_0 - \frac{\Gamma_0}{2}\right]\hat{b} - \mathrm{i}g_{0\omega}\hat{a}^\dagger\hat{a} + \mathrm{i}\sqrt{\Gamma_{\mathrm{ext}}}\hat{b}_{\mathrm{in}}. \tag{S143}$$

Now, we simplify and linearize the system by setting

$$\hat{a} = \left[\alpha_0 + \hat{c}\right]e^{\mathrm{i}\omega_{\mathrm{p}}t} \tag{S144}$$

$$\hat{b} = \beta_0 + \hat{d} \tag{S145}$$

$$\hat{a}_{\mathrm{in}} = \left[\alpha_{\mathrm{in}}e^{\mathrm{i}\phi_{\mathrm{p}}} + \hat{c}_{\mathrm{in}}\right]e^{\mathrm{i}\omega_{\mathrm{p}}t} \tag{S146}$$

$$\hat{b}_{\mathrm{in}} = \hat{d}_{\mathrm{in}} \tag{S147}$$

with the pump frequency ω_{p}. After dropping all terms not linear in $\hat{c}$ and $\hat{d}$ and neglecting the small contributions due to the terms containing the LF steady-state term $\left(\beta_0 + \beta_0^*\right) \approx 2g_{0\omega}n_{\mathrm{c}}/\Omega_0$, we find for the dynamical quantities

$$\dot{\hat{c}} = \left[-\mathrm{i}\Delta_{\mathrm{p}} - \frac{\kappa_0}{2}\right]\hat{c} - \mathrm{i}\left[g_\omega + \mathrm{i}\frac{g_\kappa}{2}\right]\left(\hat{d}+\hat{d}^\dagger\right) + \mathrm{i}\sqrt{\kappa_{\mathrm{ext}}}\hat{c}_{\mathrm{in}} \tag{S148}$$

$$\dot{\hat{d}} = \left[\mathrm{i}\Omega_0 - \frac{\Gamma_0}{2}\right]\hat{d} - \mathrm{i}g_\omega\left(\hat{c}+\hat{c}^\dagger\right) + \mathrm{i}\sqrt{\Gamma_{\mathrm{ext}}}\hat{d}_{\mathrm{in}} \tag{S149}$$

with $\Delta_{\mathrm{p}} = \omega_{\mathrm{p}} - \omega_0$, $g_\omega = \alpha_0 g_{0\omega}$ and $g_\kappa = \alpha_0 g_{0\kappa}$. Note that $\alpha_0 = \sqrt{n_{\mathrm{c}}}$ with the pump-induced intracavity photon number n_{c}. We solve these by Fourier transform and get

$$\hat{c} = -\mathrm{i}\chi_{\mathrm{c}}\left[g_\omega + \mathrm{i}\frac{g_\kappa}{2}\right]\left(\hat{d}+\hat{d}^\dagger\right) + \mathrm{i}\chi_{\mathrm{c}}\sqrt{\kappa_{\mathrm{ext}}}\hat{c}_{\mathrm{in}} \tag{S150}$$

$$\hat{d} = -\mathrm{i}\chi_0 g_\omega\left(\hat{c}+\hat{c}^\dagger\right) + \mathrm{i}\chi_0\sqrt{\Gamma_{\mathrm{ext}}}\hat{d}_{\mathrm{in}} \tag{S151}$$

with the susceptibilities

$$\chi_c = \frac{1}{\frac{\kappa_0}{2} + \mathrm{i}\left(\Delta_p + \Omega\right)}, \quad \chi_0 = \frac{1}{\frac{\Gamma_0}{2} + \mathrm{i}\left(\Omega - \Omega_0\right)} \tag{S152}$$

and the frequency with respect to the pump tone $\Omega = \omega - \omega_p$. Note again, that we do not discriminate by notation between time-domain quantities $\hat{c}(t), \hat{d}(t)$ and their Fourier transforms $\hat{c}(\Omega), \hat{d}(\Omega)$ for simplicity of the equations. In all equations discussed here, this implies that $\hat{c}^\dagger, \hat{d}^\dagger$ in the frequency domain have an inverted frequency argument, i.e., that the Fourier transforms $\hat{c}^\dagger, \hat{d}^\dagger$ shall be read as $\hat{c}^\dagger(-\Omega)$ and $\hat{d}^\dagger(-\Omega)$, respectively.

E. Dynamical backaction in the low-pump-power regime

To calculate the dynamical backaction exerted from the HF intracavity pump fields to the LF mode, we drop in Eq. (S150) the HF input term and write $g = g_\omega + \mathrm{i}g_\kappa/2$. We get

$$\hat{c} = -\mathrm{i}g\chi_c\left(\hat{d} + \hat{d}^\dagger\right) \tag{S153}$$

$$\hat{c}^\dagger = \mathrm{i}g^*\overline{\chi}_c\left(\hat{d} + \hat{d}^\dagger\right), \tag{S154}$$

or combined

$$\hat{c} + \hat{c}^\dagger = -\mathrm{i}\left[g\chi_c - g^*\overline{\chi}_c\right]\left(\hat{d} + \hat{d}^\dagger\right). \tag{S155}$$

Hence, we find for the LF mode from Eq. (S151)

$$\hat{d} = -g_\omega\chi_0\left[g\chi_c - g^*\overline{\chi}_c\right]\left(\hat{d} + \hat{d}^\dagger\right) + \mathrm{i}\chi_0\sqrt{\Gamma_{\mathrm{ext}}}\hat{d}_{\mathrm{in}} \tag{S156}$$

$$\hat{d}^\dagger = g_\omega\overline{\chi}_0\left[g\chi_c - g^*\overline{\chi}_c\right]\left(\hat{d} + \hat{d}^\dagger\right) - \mathrm{i}\overline{\chi}_0\sqrt{\Gamma_{\mathrm{ext}}}\hat{d}_{\mathrm{in}}^\dagger, \tag{S157}$$

or again combined

$$\hat{d} + \hat{d}^\dagger = -g_\omega\left[\chi_0 - \overline{\chi}_0\right]\left[g\chi_c - g^*\overline{\chi}_c\right]\left(\hat{d} + \hat{d}^\dagger\right) + \mathrm{i}\sqrt{\Gamma_{\mathrm{ext}}}\left(\chi_0\hat{d}_{\mathrm{in}} - \overline{\chi}_0\hat{d}_{\mathrm{in}}^\dagger\right). \tag{S158}$$

The latter can also be written as

$$\frac{\hat{d} + \hat{d}^\dagger}{\chi_0 - \overline{\chi}_0} = -g_\omega\left[g\chi_c - g^*\overline{\chi}_c\right]\left(\hat{d} + \hat{d}^\dagger\right) + \mathrm{i}\sqrt{\Gamma_{\mathrm{ext}}}\frac{\chi_0\hat{d}_{\mathrm{in}} - \overline{\chi}_0\hat{d}_{\mathrm{in}}^\dagger}{\chi_0 - \overline{\chi}_0}. \tag{S159}$$

Using the high-Q results

$$\chi_\Phi = \frac{1}{\Omega_0^2 - \Omega^2 + \mathrm{i}\Omega\Gamma_0} \tag{S160}$$

$$\approx 2i\Omega_0\left(\chi_0 - \overline{\chi}_0\right) \tag{S161}$$

$$\frac{\chi_0}{\chi_0 - \overline{\chi}_0} \approx 1 \quad \text{for} \quad \Omega \approx +\Omega_0 \tag{S162}$$

$$-\frac{\overline{\chi}_0}{\chi_0 - \overline{\chi}_0} \approx 1 \quad \text{for} \quad \Omega \approx -\Omega_0 \tag{S163}$$

as well as the flux $\hat{\Phi} = \Phi_{\mathrm{zpf}}\left(\hat{d} + \hat{d}^\dagger\right)$, we arrive at

$$\frac{\hat{\Phi}}{\chi_\Phi} = -2\mathrm{i}\Omega_0 g_\omega\left[g\chi_c - g^*\overline{\chi}_c\right]\hat{\Phi} - 2\Omega_0\sqrt{\Gamma_{\mathrm{ext}}}\hat{\Phi}_{\mathrm{in}} \tag{S164}$$

with $\hat{\Phi}_{\mathrm{in}} = \Phi_{\mathrm{zpf}}\left(\hat{d}_{\mathrm{in}} + \hat{d}_{\mathrm{in}}^\dagger\right)$. Finally, we combine all terms and get

$$\hat{\Phi} = -2\Omega_0\chi_\Phi^{\mathrm{eff}}\sqrt{\Gamma_{\mathrm{ext}}}\hat{\Phi}_{\mathrm{in}} \tag{S165}$$

with the effective LF susceptibility

$$\chi_\Phi^{\text{eff}} = \frac{1}{\Omega_0^2 - \Omega^2 + i\Omega\Gamma_0 + 2i\Omega_0 g_\omega \left[g\chi_c - g^*\overline{\chi}_c\right]}. \tag{S166}$$

The dynamical backaction is easier to identify in high-Q approximation for $\Omega \approx \Omega_0$ though, for which the susceptibility becomes

$$\chi_\Phi^{\text{eff}} \approx \frac{1}{2i\Omega_0}\chi_0^{\text{eff}} \tag{S167}$$

$$= \frac{1}{2i\Omega_0}\frac{1}{\frac{\Gamma_0}{2} + i(\Omega - \Omega_0) + g_\omega \left[g\chi_{c0} - g^*\overline{\chi}_{c0}\right]}, \tag{S168}$$

from where we can see that photon-pressure damping Γ_{pp} and photon-pressure frequency shift $\delta\Omega_{\text{pp}}$ are given by

$$\Gamma_{\text{pp}} = 2\text{Re}\left[g_\omega\left(g\chi_{c0} - g^*\overline{\chi}_{c0}\right)\right] \tag{S169}$$

$$\delta\Omega_{\text{pp}} = -\text{Im}\left[g_\omega\left(g\chi_{c0} - g^*\overline{\chi}_{c0}\right)\right] \tag{S170}$$

and where χ_{c0} and $\overline{\chi}_{c0}$ are the HF susceptibilities evaluated at $\Omega = \Omega_0$, i.e.,

$$\chi_{c0} = \frac{1}{\frac{\kappa_0}{2} + i\left(\Delta_p + \Omega_0\right)}, \quad \overline{\chi}_{c0} = \frac{1}{\frac{\kappa_0}{2} - i\left(\Delta_p - \Omega_0\right)}. \tag{S171}$$

Note that these relations do not contain any sideband-resolution approximation, they are fully valid for arbitrary pump detunings and contain the information from both Stokes and anti-Stokes scattering (cooling and amplification). In the sideband-resolved limit $\Omega_0^2 \gg \kappa_0^2$, which here for $\Delta_p \approx -\Omega_0$ is equivalent to $|\chi_{c0}| \gg |\overline{\chi}_{c0}|$, we would find for a red-sideband pump

$$\Gamma_{\text{pp}}^{\text{sbr}} = 2\text{Re}\left[g_\omega g\chi_{c0}\right] \tag{S172}$$

$$\delta\Omega_{\text{pp}}^{\text{sbr}} = -\text{Im}\left[g_\omega g\chi_{c0}\right]. \tag{S173}$$

F. Photon-pressure induced transparency in the low-pump-power regime

To describe the transparency experiment, we need to calculate the reflection of the HF cavity in the presence of a sideband pump. For this, we go back to Eq. (S150)

$$\frac{\hat{c}}{\chi_c} = -ig\left(\hat{d} + \hat{d}^\dagger\right) + i\sqrt{\kappa_{\text{ext}}}\hat{c}_{\text{in}} \tag{S174}$$

and combine it with the input-less relation

$$\hat{d} + \hat{d}^\dagger = -ig_\omega\left[\chi_0 - \overline{\chi}_0\right]\left(\hat{c} + \hat{c}^\dagger\right) \tag{S175}$$

and with

$$\hat{c} + \hat{c}^\dagger = -i\left[g\chi_c - g^*\overline{\chi}_c\right]\left(\hat{d} + \hat{d}^\dagger\right) + i\sqrt{\kappa_{\text{ext}}}\left(\chi_c\hat{c}_{\text{in}} - \overline{\chi}_c\hat{c}_{\text{in}}^\dagger\right). \tag{S176}$$

This leads to

$$\hat{d} + \hat{d}^\dagger = \frac{g_\omega\left[\chi_0 - \overline{\chi}_0\right]}{1 + g_\omega\left[\chi_0 - \overline{\chi}_0\right]\left[g\chi_c - g^*\overline{\chi}_c\right]}\sqrt{\kappa_{\text{ext}}}\left(\chi_c\hat{c}_{\text{in}} - \overline{\chi}_c\hat{c}_{\text{in}}^\dagger\right) \tag{S177}$$

which we use to finally find for the relevant Fourier component

$$\frac{\hat{c}}{\chi_c} = i\sqrt{\kappa_{\text{ext}}}\left[1 - \frac{g_\omega g\left(\chi_0 - \overline{\chi}_0\right)\chi_c}{1 + g_\omega\left(\chi_0 - \overline{\chi}_0\right)\left(g\chi_c - g^*\overline{\chi}_c\right)}\right]\hat{c}_{\text{in}}. \tag{S178}$$

Now we use again the high-Q approximation $2i\Omega_0\chi_\Phi = \chi_0 - \overline{\chi}_0$ to get

$$\hat{c} = i\sqrt{\kappa_{\text{ext}}}\chi_c\left[1 - \frac{2i\Omega_0 g_\omega g\chi_\Phi\chi_c}{1 + 2i\Omega_0 g_\omega\chi_\Phi\left(g\chi_c - g^*\overline{\chi}_c\right)}\right]\hat{c}_{\text{in}} \tag{S179}$$

$$= i\sqrt{\kappa_{\text{ext}}}\chi_c\left[1 - 2i\Omega_0 g_\omega g\chi_c\chi_\Phi^{\text{eff}}\right]\hat{c}_{\text{in}} \tag{S180}$$

and ultimately

$$S_{11} = 1 - \kappa_{\rm ext}\chi_{\rm c}\left[1 - 2{\rm i}\Omega_0 g_\omega g \chi_{\rm c}\chi_\Phi^{\rm eff}\right]. \tag{S181}$$

Using again $2{\rm i}\Omega_0\chi_\Phi^{\rm eff} = \chi_0^{\rm eff}$ for $\Omega \sim \Omega_0$, we find

$$S_{11} = 1 - \kappa_{\rm ext}\chi_{\rm c}\left[1 - g_\omega g \chi_{\rm c}\chi_0^{\rm eff}\right]. \tag{S182}$$

Note that we can write the effective LF suceptibility also just as

$$\chi_0^{\rm eff} = \frac{1}{\frac{\Gamma_{\rm eff}}{2} + {\rm i}\left(\Omega - \Omega_{\rm eff}\right)} \tag{S183}$$

where $\Gamma_{\rm eff}$ and $\Omega_{\rm eff}$ contain the dynamical backaction already.

G. High-pump-power regime – Equations of motion

With higher pump powers, we still use the same ansatz for solving the EOMs and arrive (after dropping constants, negligibly small terms and higher order terms) at the linearized version

$$\begin{aligned}
\dot{\hat{c}} &= -{\rm i}\left[\Delta_{\rm p} - 2\mathcal{K}n_{\rm c}\right]\hat{c} - \frac{\kappa_0 + 2\kappa_1 n_{\rm c} + 3\kappa_2 n_{\rm c}^2 + 4\kappa_3 n_{\rm c}^3}{2}\hat{c} - {\rm i}\sqrt{n_{\rm c}}\left[g_{0\omega} + g_{0\mathcal{K}}n_{\rm c} + {\rm i}\frac{g_{0\kappa} + g_{\rm nl1}n_{\rm c} + g_{\rm nl2}n_{\rm c}^2 + g_{\rm nl3}n_{\rm c}^3}{2}\right]\left(\hat{d} + \hat{d}^\dagger\right) \\
&\quad + \left[{\rm i}\mathcal{K} - \frac{\kappa_1 + 2\kappa_2 n_{\rm c} + 3\kappa_3 n_{\rm c}^2}{2}\right] n_{\rm c}\hat{c}^\dagger + {\rm i}\sqrt{\kappa_{\rm ext}}\hat{c}_{\rm in} \qquad \text{(S184)} \\
\dot{\hat{d}} &= {\rm i}\left[\Omega_0 + \mathcal{K}_{\rm c}n_{\rm c}\right]\hat{d} - \frac{\Gamma_0 + \kappa_{\rm c}n_{\rm c}}{2}\hat{d} - {\rm i}\sqrt{n_{\rm c}}\left[g_{0\omega} + g_{0\mathcal{K}}n_{\rm c}\right]\left(\hat{c} + \hat{c}^\dagger\right) + {\rm i}\sqrt{\Gamma_{\rm ext}}\hat{d}_{\rm in}. \qquad \text{(S185)}
\end{aligned}$$

Now, we can define the effective multi-photon coupling rates

$$\begin{aligned}
g_\omega' &= g_{0\omega}\sqrt{n_{\rm c}} + g_{0\mathcal{K}}\sqrt{n_{\rm c}^3} \qquad \text{(S186)} \\
g_\kappa' &= g_{0\kappa}\sqrt{n_{\rm c}} + g_{\rm nl1}\sqrt{n_{\rm c}^3} + g_{\rm nl2}\sqrt{n_{\rm c}^5} + g_{\rm nl3}\sqrt{n_{\rm c}^7} \qquad \text{(S187)}
\end{aligned}$$

which for large photon numbers scale much stronger with power than in usual optomechanics or photon-pressure.

Since with our pump tone, we work in a regime of very small intracavity gain and very small four-wave mixing due to the large detuning and the high nonlinear damping (for a more strict treatment see Sec. 4 and Refs. [12, 13, 18]), the Kerr frequency shifts and nonlinear dampings can just be included by using modified frequencies and linewidths, labeled as ω_0', κ_0', Ω_0' and Γ_0', just as described above in Sec. 4 and we get

$$\begin{aligned}
\dot{\hat{c}} &= -\left[{\rm i}\Delta_{\rm p}' + \frac{\kappa_0'}{2}\right]\hat{c} - {\rm i}\left[g_\omega' + {\rm i}\frac{g_\kappa'}{2}\right]\left(\hat{d} + \hat{d}^\dagger\right) + {\rm i}\sqrt{\kappa_{\rm ext}}\hat{c}_{\rm in} \qquad \text{(S188)} \\
\dot{\hat{d}} &= \left[{\rm i}\Omega_0' - \frac{\Gamma_0'}{2}\right]\hat{d} - {\rm i}g_\omega'\left(\hat{c} + \hat{c}^\dagger\right) + {\rm i}\sqrt{\Gamma_{\rm ext}}\hat{d}_{\rm in} \qquad \text{(S189)}
\end{aligned}$$

where for our experimental conditions (red-sideband pumping)

$$\begin{aligned}
\omega_0' &= \omega_0 + \Delta_{\rm p} + \sqrt{\left(\Delta_{\rm p} - \mathcal{K}n_{\rm c}\right)\left(\Delta_{\rm p} - 3\mathcal{K}n_{\rm c}\right) - \frac{\tilde{\kappa}_{\rm nl}^2 n_{\rm c}^2}{4}} \qquad \text{(S190)} \\
\kappa_0' &= \kappa_0 + 2\kappa_1 n_{\rm c} + 3\kappa_2 n_{\rm c}^2 + 4\kappa_3 n_{\rm c}^3 \qquad \text{(S191)} \\
\Omega_0' &= \Omega_0 + \mathcal{K}_{\rm c}n_{\rm c} \qquad \text{(S192)} \\
\Gamma_0' &= \Gamma_0 + \kappa_{\rm c}n_{\rm c}. \qquad \text{(S193)}
\end{aligned}$$

Beautifully, however, Eqs. (S188, S189) are completely identical to their corresponding equations in the low-power regime, just with primed (i.e. pump-shifted but constant during each VNA scan) quantities. Therefore, we can just apply all our former analyses and results.

H. Dynamical backaction in the high-pump-power regime

We find, analogous to Eqs. (S169, S170),

$$\Gamma_{\rm pp} = 2{\rm Re}\left[g'_\omega\left(g'\chi'_{\rm c0} - g'^*\overline{\chi}'_{\rm c0}\right)\right] \tag{S194}$$

$$\tag{S195}$$

$$\delta\Omega_{\rm pp} = -{\rm Im}\left[g'_\omega\left(g'\chi'_{\rm c0} - g'^*\overline{\chi}'_{\rm c0}\right)\right] \tag{S196}$$

where $\chi_{\rm c0}$ and $\overline{\chi}_{\rm c0}$ are the HF susceptibilities evaluated at $\Omega = \Omega'_0$, cf. Eq. (S171), i.e.,

$$\chi'_{\rm c0} = \frac{1}{\frac{\kappa'_0}{2} + {\rm i}\left(\Delta'_{\rm p} + \Omega'_0\right)}, \qquad \overline{\chi}'_{\rm c0} = \frac{1}{\frac{\kappa'_0}{2} - {\rm i}\left(\Delta'_{\rm p} - \Omega'_0\right)}. \tag{S197}$$

I. Photon-pressure induced transparency in the high-pump-power regime

For the reflection we obtain (high-Q limit, cf. Eq. (S182))

$$S_{11} = 1 - \kappa_{\rm ext}\chi'_{\rm c}\left[1 - g'_\omega g'\chi'_{\rm c}\chi_0^{\rm eff}\right]. \tag{S198}$$

where

$$\chi'_{\rm c} = \frac{1}{\frac{\kappa'_0}{2} + {\rm i}\left(\Delta'_{\rm p} + \Omega\right)} \tag{S199}$$

$$\overline{\chi}'_{\rm c} = \frac{1}{\frac{\kappa'_0}{2} - {\rm i}\left(\Delta'_{\rm p} - \Omega\right)} \tag{S200}$$

$$\chi_0^{\rm eff} = \frac{1}{\frac{\Gamma'_0}{2} + {\rm i}\left(\Omega - \Omega'_0\right) + g'_\omega\left(g'\chi'_{\rm c} - g'^*\overline{\chi}'_{\rm c}\right)}. \tag{S201}$$

It seems we somewhat inconsistently did not prime $\chi_0^{\rm eff}$, but it is on purpose to illustrate that we still write it as

$$\chi_0^{\rm eff} = \frac{1}{\frac{\Gamma_{\rm eff}}{2} + {\rm i}\left(\Omega - \Omega_{\rm eff}\right)} \tag{S202}$$

$$\Gamma_{\rm eff} = \Gamma_0 + \kappa_{\rm c}n_{\rm c} + \Gamma_{\rm pp} \tag{S203}$$

$$\Omega_{\rm eff} = \Omega_0 + \mathcal{K}_{\rm c}n_{\rm c} + \delta\Omega_{\rm pp}, \tag{S204}$$

since effective quantities stay effective quantities, although in detail they have one more term now.

6. SUPPLEMENTARY NOTE 6: EFFECTS OF DISSIPATIVE COUPLING

This section is meant to visualize the qualitative and quantitative changes for both PPIT and dynamical backaction, when a dissipative contribution is added to the dispersive photon-pressure interaction, and to introduce in this context the cross-cooperativity discussed in the main manuscript. Additionally, we show how the normal-mode-splitting in the strong-coupling regime would reveal itself in the presence of $g_\kappa \neq 0$, with an exciting and surprising prospect.

A. Dynamical backaction and cross-cooperativity

In Supplementary Fig. 9 we plot the two components of dynamical backaction (neglecting any nonlinearities, so no primed quantities here)

$$\Gamma_{\rm pp} = 2{\rm Re}\left[g_\omega\left(g\chi_{\rm c0} - g^*\overline{\chi}_{\rm c0}\right)\right] \tag{S205}$$

$$\delta\Omega_{\rm pp} = -{\rm Im}\left[g_\omega\left(g\chi_{\rm c0} - g^*\overline{\chi}_{\rm c0}\right)\right] \tag{S206}$$

for four different ratios of $g_\kappa/g_\omega = (0, -2, -4, -6)$ and for parameters close to the ones of our experiment. In detail, we chose $\omega_0 = 2\pi \times 8.5\,$GHz, $\kappa_0 = 2\pi \times 100\,$MHz, $\Omega_0 = 2\pi \times 450\,$MHz, $\Gamma_0 = 2\pi \times 500\,$kHz, $g_{0\omega} = 2\pi \times 30\,$kHz, and calculate the detuning-dependent pump photon number for $g_\omega = \sqrt{n_{\rm c}}g_{0\omega}$ via the linear-cavity relation

$$n_{\rm c} = \frac{4P_{\rm p}}{\hbar\omega_{\rm p}}\frac{\kappa_{\rm ext}}{\kappa_0^2 + 4\Delta_{\rm p}^2} \tag{S207}$$

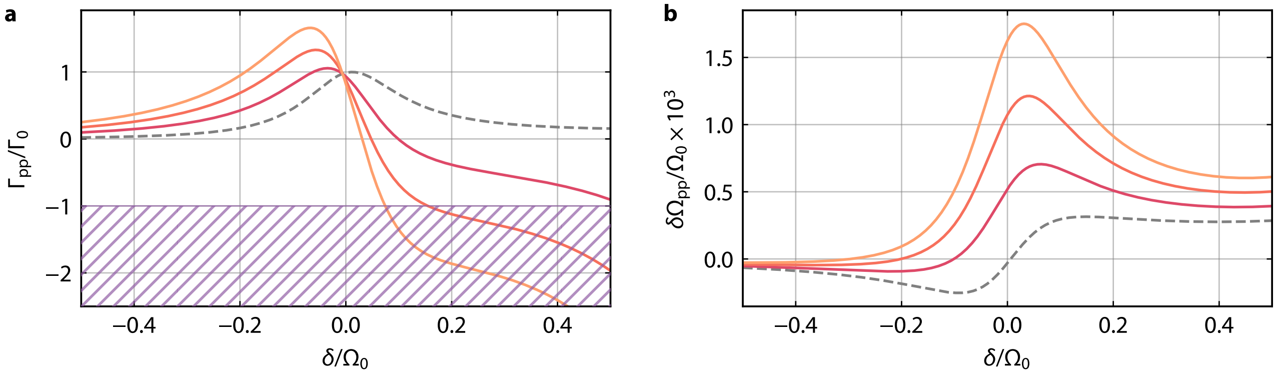

Supplementary Figure 9. **Red-sideband dynamical backaction for increasing contribution of internal-dissipative photon-pressure.** **a** Calculated photon-pressure damping Γ_{pp} and **b** calculated photon-pressure frequency shift $\delta\Omega_{pp}$ vs. pump detuning $\delta = \omega_p - (\omega_0 - \Omega_0)$ from the red sideband for four different ratios of $g_\kappa/g_\omega = (0, -2, -4, -6)$ and $g_\omega(\delta = 0) = 2\pi \times 3.52$ MHz. Dashed gray line is the purely dispersive case $g_\kappa = 0$, and lines in red, dark orange, and bright orange correspond to $g_\kappa/g_\omega = -2$, $g_\kappa/g_\omega = -4$, and $g_\kappa/g_\omega = -6$, respectively. Quantities are normalized to the bare LF mode characteristics Γ_0 and Ω_0, parameters of the calculation are $\omega_0 = 2\pi \times 8.5$ GHz, $\kappa_0 = 2\pi \times 100$ MHz, $\Omega_0 = 2\pi \times 450$ MHz, $\Gamma_0 = 2\pi \times 500$ kHz, $g_{0\omega} = 2\pi \times 30$ kHz. Pump power $P_p = -48.7$ dBm is constant, which means n_c increases with the pump moving to higher frequencies, cf. Eq. (S207), which leads to e.g. the curves for $g_\kappa = 0$ being clearly asymmetric with respect to $\delta = 0$. With increasing $|g_\kappa/g_\omega|$, both the absolute values of the dynamical backaction and the Fano-like distortion grow. Note that Γ_{pp} is enhanced for $\delta \lesssim 0$ by g_κ and pushed to smaller and eventually even negative values for $\delta \gtrsim 0$. For $\Gamma_{pp}/\Gamma_0 \leq -1$, the system will experience parametric instability (indicated by the hatched area in **a**).

where $\kappa_{ext} = 2\pi \times 7$ MHz and $\Delta_p = \omega_p - \omega_0$. The theoretical lines are shown vs. pump detuning from the red sideband $\delta = \omega_p - (\omega_0 - \Omega_0)$ and we limit our considerations to detunings between $\delta_{min} = 2\pi \times -225$ MHz and $\delta_{max} = 2\pi \times 225$ MHz (equivalent to $-\Omega_0/2$ and $+\Omega_0/2$), since this is the most relevant for our experiment. For the on-chip pump power, we use $P_p = 13.5$ nW or -48.7 dBm, which was chosen such that in the dispersive-only case and with the pump exactly on the red sideband $\Gamma_{pp}(\delta = 0) \approx \Gamma_0$, which is equivalent to $n_c(\delta = 0) \approx 13750$ and $g_\omega(\delta = 0) = 2\pi \times 3.52$ MHz.

The plots in Supplementary Fig. 9 reveal, that adding the dissipative interaction has considerable effects on the dynamical backaction. Without the dissipative part (gray dashed lines), the backaction terms describe essentially a complex Lorentzian with a detuning-dependent scaling factor $\propto n_c(\delta)/n_c(0)$, which causes the asymmetry between positive and negative δ. With $g_\kappa \neq 0$ some characteristic features of coupling interference can be identified. Most obviously there is a Fano-like distortion of the complex Lorentzian, which grows with increasing $|g_\kappa/g_\omega|$. In other words, the presence of g_κ changes the backaction magnitude as a function of frequency; for $\delta \lesssim 0$ the damping increases, while for $\delta \gtrsim 0$ the dissipative contribution counteracts the dispersive one. This even implies that in the regime $\delta > 0$, but still around the red HF cavity sideband, there will be a parametric instability $\Gamma_{eff} = \Gamma_0 + \Gamma_{pp} < 0$, which we also found in experiments, cf. main paper Fig. 5. Another consequence of this frequency-dependent sign change is that the maximum of Γ_{pp} is both considerably red-detuned from $\omega_0 - \Omega_0$ and larger than the maximum for $g_\kappa = 0$. For the PP frequency-shift $\delta\Omega_{pp}$, the effect of g_κ seems somewhat less complex, it mainly moves $\delta\Omega_{pp}$ towards larger values and makes the overall shape of the frequency-dependence more peak-like. The position of the maximum, however, also shifts as a function of g_κ/g_ω. The latter is no coincidence – for very large ratios $|g_\kappa/g_\omega| \gtrsim 100$, the shapes of Γ_{pp} and $\delta\Omega_{pp}$ would be identical to the dispersive-only case, but swapped (the relative scaling would not be swapped though).

It is useful to look at this swap on a more formal level, which will illustrate at the same time the meaning of the two cooperativities discussed in the main paper. For simplicity, we do this in the sideband-resolved approximation, which describes our device sufficiently well. In this limit, the backaction terms are given by

$$\Gamma_{pp} = 2\text{Re}\left[g_\omega g \chi_{c0}\right] \tag{S208}$$
$$\delta\Omega_{pp} = -\text{Im}\left[g_\omega g \chi_{c0}\right] \tag{S209}$$

which can be explicitly written as

$$\Gamma_{pp} = g_\omega^2 \kappa_0 |\chi_{c0}|^2 + g_\omega g_\kappa \delta |\chi_{c0}|^2 \tag{S210}$$
$$\delta\Omega_{pp} = g_\omega^2 \delta |\chi_{c0}|^2 - \frac{g_\omega g_\kappa}{4} \kappa_0 |\chi_{c0}|^2 \tag{S211}$$

using

$$\chi_{c0} = \frac{\frac{\kappa_0}{2}}{\frac{\kappa_0^2}{4} + \delta^2} - i\frac{\delta}{\frac{\kappa_0^2}{4} + \delta^2} \tag{S212}$$

$$= \frac{\kappa_0}{2}|\chi_{c0}|^2 - i\delta|\chi_{c0}|^2. \tag{S213}$$

In the dispersive-only case, the relations would read

$$\Gamma_{pp}^{\omega} = g_\omega^2 \kappa_0 |\chi_{c0}|^2 \tag{S214}$$

$$\delta\Omega_{pp}^{\omega} = g_\omega^2 \delta |\chi_{c0}|^2, \tag{S215}$$

where Γ_{pp}^{ω} has its maximum for frequency-independent g_ω, g_κ at the maximum of $|\chi_{c0}|^2$, i.e., at $\delta = 0$, and $\delta\Omega_{pp}^{\omega}$ has its maximum at the maximum of $\delta|\chi_{c0}|^2$, which is at $\delta = \kappa_0/2$. So we can express

$$\Gamma_{pp}^{\omega,\max} = \frac{4g_\omega^2}{\kappa_0} \tag{S216}$$

$$= \mathcal{C}_\omega \Gamma_0 \tag{S217}$$

$$\delta\Omega_{pp}^{\omega,\max} = \frac{1}{4}\mathcal{C}_\omega \Gamma_0 \tag{S218}$$

with the dispersive cooperativity

$$\mathcal{C}_\omega = \frac{4g_\omega^2}{\kappa_0 \Gamma_0} \tag{S219}$$

as a measure for the maximal dynamical backaction.

The new terms in the backaction arising from $g_\kappa \neq 0$ have the frequency-dependence and the position of the maxima swapped. The second term in the photon-pressure damping is

$$\Gamma_{pp}^{\kappa} = g_\omega g_\kappa \delta |\chi_{c0}|^2 \tag{S220}$$

and so for frequency-independent g_ω, g_κ it has the maximum at $\delta = -\kappa_0/2$ for $g_\kappa < 0$. Note that this new damping term is equivalent to the dispersive-only frequency-shift term, except for one g_ω being replaced by g_κ (and the corresponding sign change, which would not be there for $g_\kappa > 0$). Hence, we can write

$$\Gamma_{pp}^{\kappa,\max} = -\frac{1}{2}C_{\omega\kappa}\Gamma_0 \tag{S221}$$

with the cross-cooperativity

$$\mathcal{C}_{\omega\kappa} = \frac{2g_\omega g_\kappa}{\kappa_0 \Gamma_0}. \tag{S222}$$

Similarly, we can write the cross-cooperativity induced frequency-shift

$$\delta\Omega_{pp}^{\kappa} = -\frac{g_\omega g_\kappa}{4}\kappa_0 |\chi_{c0}|^2 \tag{S223}$$

at its maximum at $\delta = 0$ as

$$\delta\Omega_{pp}^{\kappa,\max} = -\frac{1}{2}C_{\omega\kappa}\Gamma_0. \tag{S224}$$

Note that choosing the prefactor 2 instead of 4 in the cross-cooperativity is somewhat arbitrary, but doing so reflects the factor $1/2$ entering the total complex coupling

$$g = g_\omega + i\frac{g_\kappa}{2}. \tag{S225}$$

It furthermore leads to the symmetric situation, that for $\mathcal{C}_\omega = \mathcal{C}_{\omega\kappa}$ the term $\propto\delta$ in each of the two dynamical backaction terms is half as large at $\delta = \kappa_0/2$ as the one $\propto\kappa_0$ at $\delta = 0$. Note the different scalings though; while the cross-terms lead to identical maximum values of Γ_{pp}^{κ} and $\delta\Omega_{pp}^{\kappa}$, the dispersive-only terms differ by a factor 4 at their respective maxima.

Although we did not measure the dynamical backaction with a blue-detuned pump tone, it is useful to look at the corresponding theoretical result using Eqs. (S205, S206). In Supplementary Fig. 10 this is shown as a function of pump detuning from the

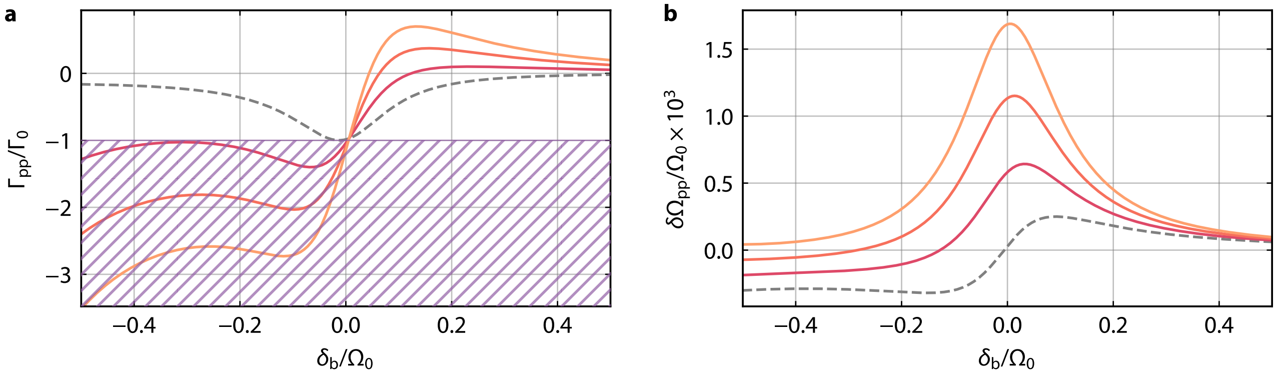

Supplementary Figure 10. **Blue-sideband dynamical backaction for increasing contribution of internal-dissipative photon-pressure.** **a** Calculated photon-pressure damping Γ_{pp} and **b** calculated photon-pressure frequency shift $\delta\Omega_{pp}$ vs. pump detuning $\delta_b = \omega_p - (\omega_0 + \Omega_0)$ from the blue sideband for four different ratios of $g_\kappa/g_\omega = (0, -2, -4, -6)$ and $g_\omega(\delta_b = 0) = 2\pi \times 3.52\,$MHz. Dashed gray line is the purely dispersive case $g_\kappa = 0$, and lines in red, dark orange, and bright orange correspond to $g_\kappa/g_\omega = -2$, $g_\kappa/g_\omega = -4$, and $g_\kappa/g_\omega = -6$, respectively. Quantities are normalized to the bare LF mode characteristics Γ_0 and Ω_0, parameters of the calculation are $\omega_0 = 2\pi \times 8.5\,$GHz, $\kappa_0 = 2\pi \times 100\,$MHz, $\Omega_0 = 2\pi \times 450\,$MHz, $\Gamma_0 = 2\pi \times 500\,$kHz, $g_{0\omega} = 2\pi \times 30\,$kHz. Pump power $P_p = 15\,$nW is constant, which means n_c decreases with the pump moving to higher frequencies, cf. Eq. (S207), which leads to e.g. the curves for $g_\kappa = 0$ being clearly asymmetric with respect to $\delta_b = 0$. With increasing $|g_\kappa/g_\omega|$, both the absolute values of the dynamical backaction and the Fano-like distortion grow. Note that Γ_{pp} is enhanced for $\delta_b \lesssim 0$ by g_κ and pushed to positive values for $\delta_b \gtrsim 0$, which corresponds to blue-sideband cooling. For $\Gamma_{pp}/\Gamma_0 \leq -1$, the system will experience parametric instability (indicated by the hatched area in **a**).

blue sideband $\delta_b = \omega_p - (\omega_0 + \Omega_0)$, and in complete correspondence to Supplementary Fig. 9. Again, for $g_\kappa = 0$ (gray dashed line) we find the familiar complex Lorentzian with negative photon-pressure damping (i.e. amplification) and a photon-pressure frequency-shift that resembles the one on the red sideband. All parameters are identical to the red-sideband case in Supplementary Fig. 9, except for the pump power $P_p = 15\,$nW, which we slightly increased to again have $n_c(\delta_b = 0) \approx 13750$, $g_\omega(\delta_b = 0) = 2\pi \times 3.52\,$MHz and $\Gamma_{pp}(\delta_b = 0) = -\Gamma_0$. Since we still calculate the pump photon number n_c via Eq. (S207), now the photon number increases with negative detunings, which causes the asymmetry between positive and negative δ_b to show the opposite trend of the red-sideband case. Aside from this, the frequency shift $\delta\Omega_{pp}$ is modified by $g_\kappa \neq 0$ essentially the same way as on the red sideband; with increasing $|g_\kappa/g_\omega|$ it turns more and more into a Lorentzian-like peak and the maximum is approaching $\delta_b \approx 0$. The photon-pressure damping on the other hand looks not mirrored at all when comparing it to the red-sideband case, but there is nevertheless a certain symmetry. For $\delta_b > 0$, the dispersive backaction damping $\Gamma_{pp} < 0$ is overcompensated by the dissipative contribution to $\Gamma_{pp} > 0$, which in fact corresponds to (counterintuitive) cooling on the blue sideband. For $\delta_b < 0$ the negative backaction damping is strongly enhanced by the dissipative contribution, leading to parametric instability over the complete range of detunings for the parameters used here.

To conclude this section, we show in Supplementary Fig. 11 the dynamical backaction over the complete pump frequency range from $\omega_p = \omega_0 - 1.5\Omega_0$ to $\omega_p = \omega_0 + 1.5\Omega_0$. In contrast to Supplementary Figs. 9 and 10 we do it this time for constant pump photon number $n_c = 13750$ as a function of pump detuning instead of constant pump power for two reasons. First, for $g_\kappa = 0$, it reveals both the point-symmetry around $\Delta_p = 0$ and the (nearly) symmetric Lorentzian around the two sidebands, which was masked above by the detuning-dependent g_ω. It also reveals the lack of that symmetry for $g_\kappa \neq 0$. The remaining asymmetries in $\delta\Omega_{pp}$ around the two sidebands originate from imperfect sideband-resolution. And secondly, since otherwise the backaction on resonance $\Delta_p = 0$ for $g_\kappa \neq 0$ is so large that the shape of the contributions on the sidebands are not easily recognizable anymore. Most of the relevant features, that can be identified in the dynamical backaction, are already known at this point and discussed in the context of Supplementary Figs. 9 and 10. What is new here is the backaction on and around resonance, i.e., for $\Delta_p = 0$. While for $g_\kappa = 0$ the dynamical backaction identically vanishes for $\omega_p = \omega_0$, it is finite for $g_\kappa \neq 0$. Due to the opposite signs of g_ω and g_κ it leads to negative backaction damping and hence amplification on resonance, while for identical signs it would lead to cooling on resonance. Such identical signs could be achieved in the future by e.g. a properly designed external-dissipative coupling $g_{\kappa_{ext}}$ or by a modified circuit layout that contains a flux-dependent path through an additional on-chip resistor in series with the SQUID.

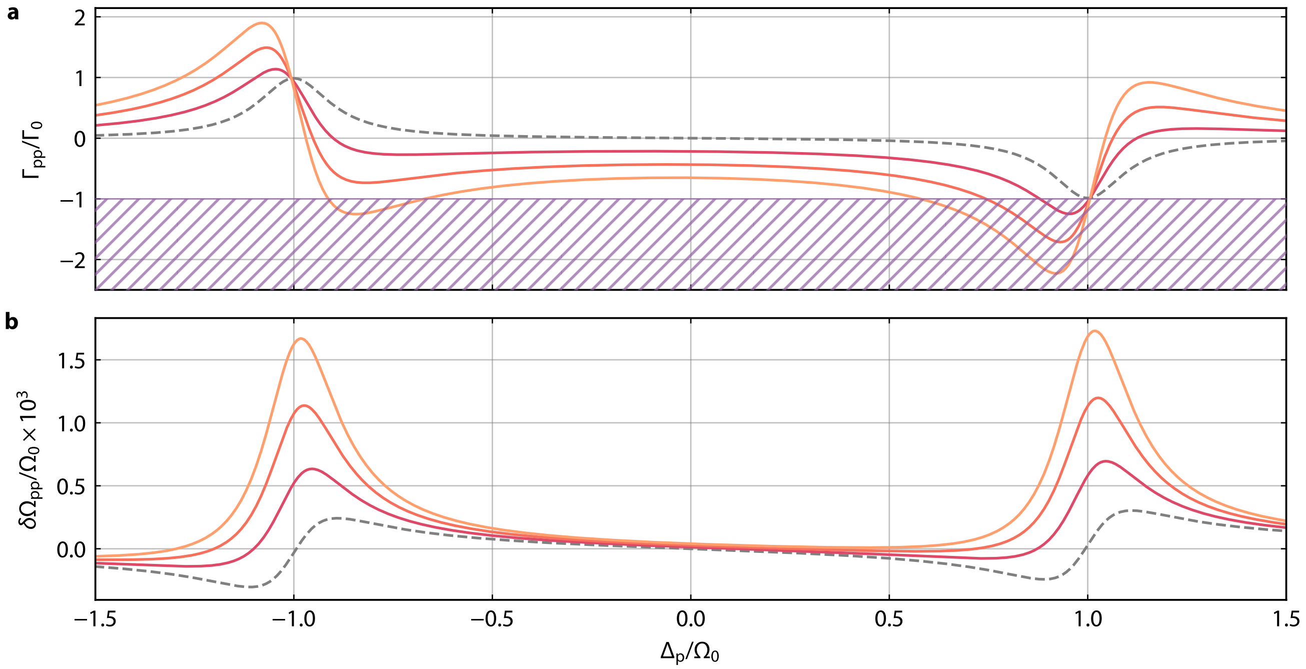

Supplementary Figure 11. **Broadband dynamical backaction for increasing contribution of internal-dissipative photon-pressure.** **a** Calculated photon-pressure damping Γ_{pp} and **b** calculated photon-pressure frequency shift $\delta\Omega_{pp}$ vs. pump detuning from the cavity resonance $\Delta_p = \omega_p - \omega_0$ for four different ratios of $g_\kappa/g_\omega = (0, -2, -4, -6)$ and $g_\omega = 2\pi \times 3.52\,\text{MHz}$ for all pump frequencies. Dashed gray line is the purely dispersive case $g_\kappa = 0$, and lines in red, dark orange, and bright orange correspond to $g_\kappa/g_\omega = -2$, $g_\kappa/g_\omega = -4$, and $g_\kappa/g_\omega = -6$, respectively. Quantities are normalized to the bare LF mode characteristics Γ_0 and Ω_0, parameters of the calculation are $\omega_0 = 2\pi \times 8.5\,\text{GHz}$, $\kappa_0 = 2\pi \times 100\,\text{MHz}$, $\Omega_0 = 2\pi \times 450\,\text{MHz}$, $\Gamma_0 = 2\pi \times 500\,\text{kHz}$, $g_{0\omega} = 2\pi \times 30\,\text{kHz}$, and intracavity pump photon number $n_c = 13750$ independent of Δ_p. With increasing $|g_\kappa/g_\omega|$, both the absolute values of the dynamical backaction and the Fano-like distortion grow. Interestingly, the dynamical backaction does not vanish on resonance, when $g_\kappa \neq 0$, which here and due to $g_\kappa < 0$ leads to LF amplification on resonance, and for $g_\kappa > 0$ would lead to cooling on resonance. For $\Gamma_{pp}/\Gamma_0 \leq -1$, the system will experience parametric instability (indicated by the hatched area in **a**).

B. Photon-pressure induced transparency

Next, we plot the calculated HF reflection for the same parameters as the dynamical backaction in the previous section, but for a single pump frequency $\omega_p = \omega_0 - \Omega_{\text{eff}}$ in all four cases. So again, the coupling ratios are $g_\kappa/g_\omega = (0, -2, -4, -6)$ and we plug everything into Eq. (S182)

$$S_{11} = 1 - \kappa_{\text{ext}}\chi_c\left(1 - g_\omega g \chi_c \chi_0^{\text{eff}}\right). \tag{S226}$$

The result is shown in Supplementary Fig. 12, and illustrates the essential characteristics of the dissipative coupling contribution to PPIT.

For $g_\kappa = 0$, the PPIT resonance is a symmetric peak in $|S_{11}|$, sitting at the bottom of the HF absorption dip; it becomes increasingly asymmetric and Fano-like with growing g_κ/g_ω. The origin of this asymmetry is the interference of the sidebands with amplitude $\propto g_\omega$ and $\propto i g_\kappa/2$, which leads to a rotation of the PPIT resonance circle in the complex plane. The PPIT resonance peak-height and circle diameter, respectively, also grow with increasing g_κ due to the increased total coupling rate. As a measure for g_κ/g_ω we can consider the tilt angle γ in the complex plane, which is given by

$$\gamma = \arctan\frac{g_\kappa}{2g_\omega} \tag{S227}$$

and which could also be explicitly introduced into Eq. (S226) by

$$g = g_\omega + i\frac{g_\kappa}{2} \tag{S228}$$

$$= |g|e^{i\gamma} \tag{S229}$$

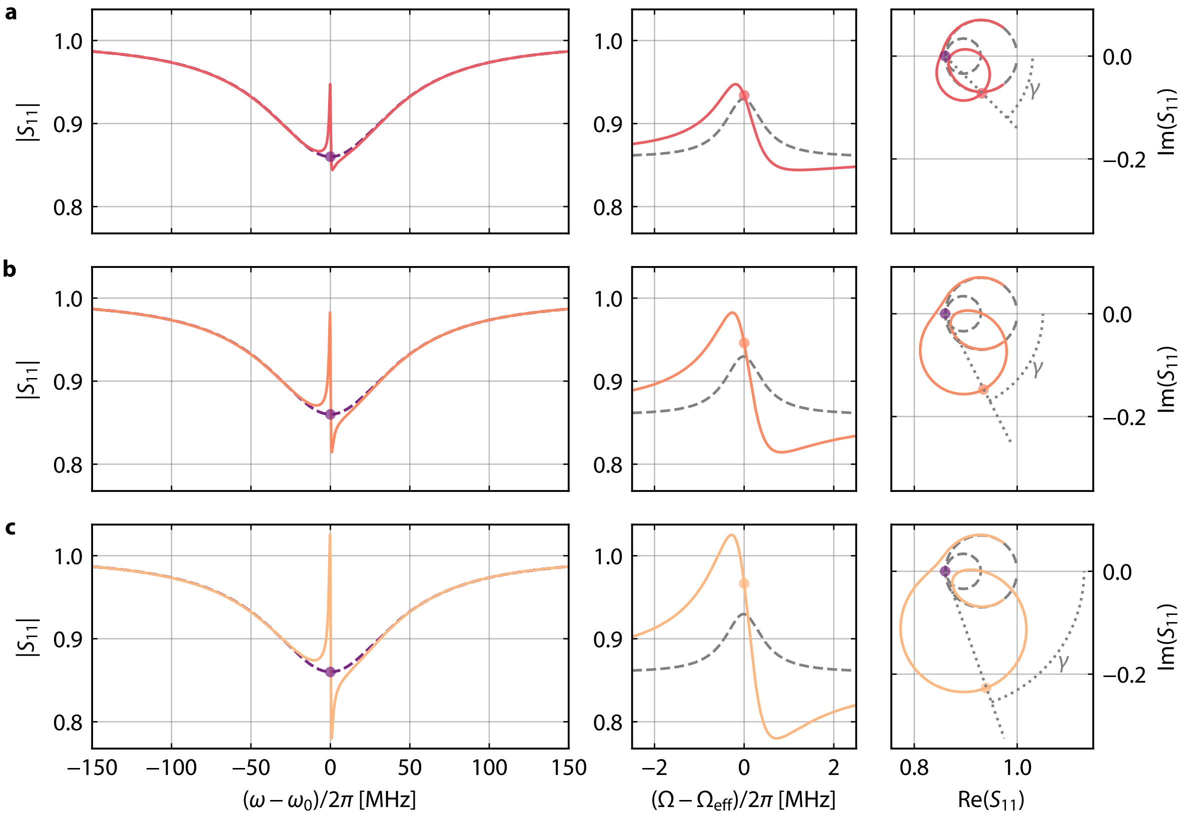

Supplementary Figure 12. **Photon-pressure-induced transparency for increasing contribution of internal-dissipative coupling.** All subpanels show the calculated HF cavity reflection S_{11} using Eq. (S226) in a resonant PPIT setting, i.e., with a pump tone at $\omega_\mathrm{p} = \omega_0 - \Omega_\mathrm{eff}$. Parameters for HF mode, LF mode and the coupling rates are given in subsection 6 A. From top column to bottom column, the dissipative coupling increases, in **a** $g_\kappa/g_\omega = -2$, in **b** $g_\kappa/g_\omega = -4$, and in **c** $g_\kappa/g_\omega = -6$. Left image shows magnitude of S_{11} in a frequency span $3\kappa_0$ around ω_0, center image shows a magnification of the transparency window with a frequency span $10\Gamma_0$, and right image shows the circle plot of the PPIT resonance and cavity resonance in the complex plane. Purple disks in left and right images show the point of S_{11} on HF cavity resonance without the pump tone, orange disks in center and right panels show the point of PPIT resonance. Dotted straight lines connecting the two disks in the circle plots define the angle γ between that line and the real axis. For reference, each zoom and complex plot (center and right, respectively) also shows the case $g_\kappa = 0$ as gray dashed line; the left images contain the cavity resonance for $g_\kappa = g_\omega = 0$ as purple dashed line. With increasing g_κ/g_ω, the PPIT circle tilts more and more away from the real axis, i.e., the angle $\gamma = \arctan g_\kappa/2g_\omega$ increases, leading to an increasing Fano lineshape of the transparency resonance in $|S_{11}|$. For large g_κ/g_ω, the PPIT circle gets larger than the cavity circle, despite being in the sideband-resolved regime, even leading to output gain $|S_{11}| > 1$ in **c**.

and so

$$S_{11} = 1 - \kappa_\mathrm{ext}\chi_\mathrm{c}\left(1 - g_\omega|g|e^{i\gamma}\chi_\mathrm{c}\chi_0^\mathrm{eff}\right). \tag{S230}$$

This form very much illustrates that the resonance circle of the PPIT, described by the second term in parenthesis, is rotated by γ around its anchor point.

C. The strong-coupling regime

Similar to the dispersive-only case, we can calculate the normal modes of the system in the presence of dissipative coupling (even though we cannot yet reach the strong-coupling regime in the experiment). We do this for simplicity again for the sideband-resolved regime $4\Omega_0^2 \gg \kappa_0^2$. The effective susceptibility of the HF mode in this case is

$$\chi_\mathrm{c}^\mathrm{eff} = \frac{\chi_\mathrm{c}}{1 + g_\omega g\chi_\mathrm{c}\chi_0}. \tag{S231}$$

The resonances can be found by the condition

$$1 + g_\omega g \chi_c \chi_0 = 0 \tag{S232}$$

or

$$\frac{1}{\chi_c \chi_0} + g_\omega g = 0. \tag{S233}$$

The latter is equivalent to a quadratic equation in ω and we can find the two complex-valued solutions $\tilde{\omega}_\pm$ from

$$0 = \left[\frac{\Gamma_0}{2} + \mathrm{i}\left(\tilde{\Omega} - \Omega_0\right)\right]\left[\frac{\kappa_0}{2} + \mathrm{i}\left(\tilde{\omega} - \omega_0\right)\right] + g_\omega^2 + \mathrm{i}\frac{g_\omega g_\kappa}{2} \tag{S234}$$

$$= \left[\frac{\Gamma_0}{2} + \mathrm{i}\left(\tilde{\omega} - \omega_0 - \delta\right)\right]\left[\frac{\kappa_0}{2} + \mathrm{i}\left(\tilde{\omega} - \omega_0\right)\right] + g_\omega^2 + \mathrm{i}\frac{g_\omega g_\kappa}{2} \tag{S235}$$

$$= -\tilde{\omega}^2 + \tilde{\omega}\left[2\omega_0 + \delta + \mathrm{i}\frac{\kappa_0 + \Gamma_0}{2}\right] + \frac{\kappa_0 \Gamma_0}{4} - \mathrm{i}\frac{\kappa_0 + \Gamma_0}{2}\omega_0 - \mathrm{i}\frac{\kappa_0}{2}\delta - \omega_0^2 - \omega_0\delta + g_\omega^2 + \mathrm{i}\frac{g_\omega g_\kappa}{2} \tag{S236}$$

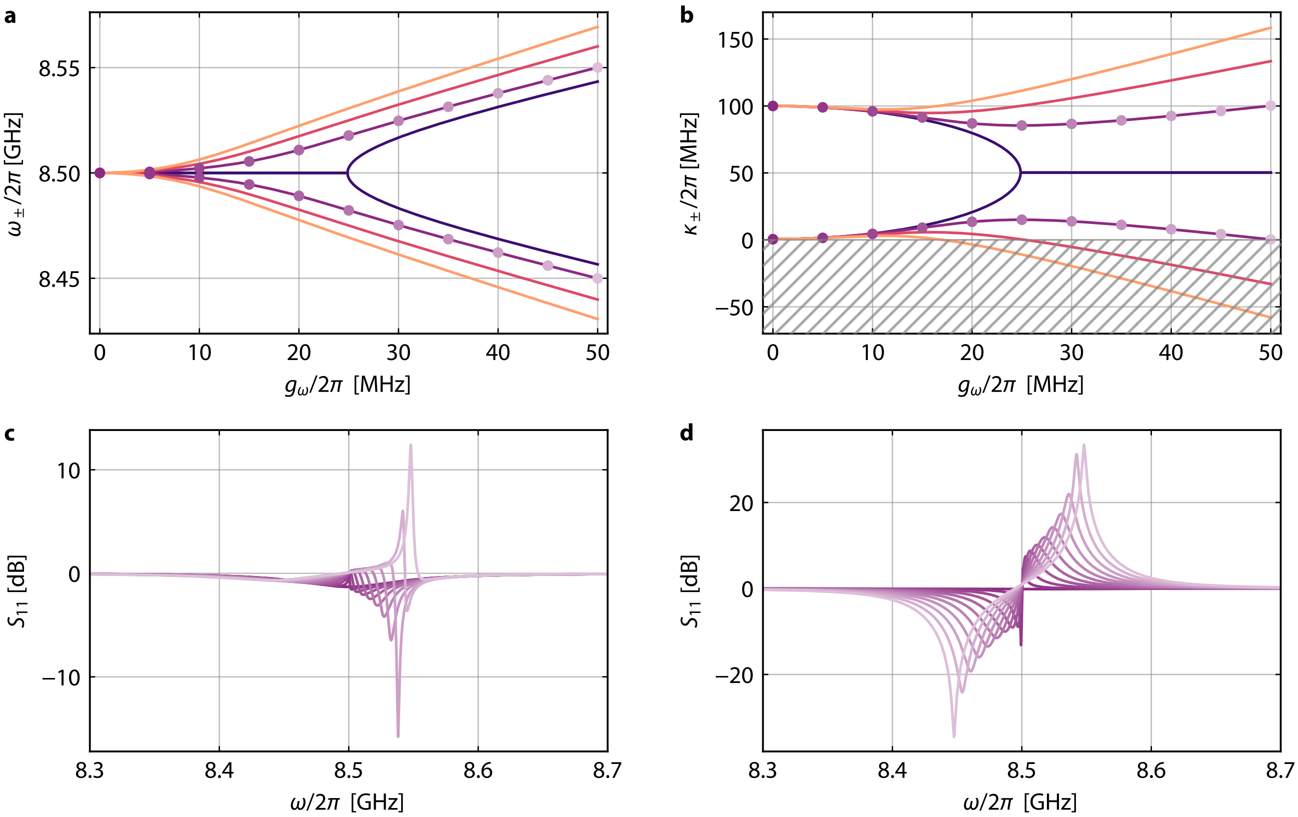

Supplementary Figure 13. **Normal modes and amplification in the dispersive-plus-dissipative strong-coupling regime. a** Normal-mode resonance frequencies $\omega_\pm = \mathrm{Re}(\tilde{\omega}_\pm)$ and **b** normal-mode linewidths $\kappa_\pm = 2\mathrm{Im}(\tilde{\omega}_\pm)$ of a photon-pressure system with dispersive-plus-dissipative coupling for the four ratios $g_\kappa/g_\omega = (0, -2, -4, -6)$ according to Eq. (S237); from innermost to outermost curve g_κ/g_ω increases. System parameters are chosen close to those of our device, cf. sections 6 A and 6 B, pump frequency is $\omega_\mathrm{p} = \omega_0 - \Omega_0$. For vanishing g_κ (dark blue curves) the usual normal-mode splitting is observed, where for the coupling strength $g_\omega < (\kappa_0 - \Gamma_0)/4$ the resonance frequencies of the two modes coincide and the two linewidths differ, while for $g_\omega > (\kappa_0 - \Gamma_0)/4$ it is vice versa with both modes having the linewidth $(\kappa_0 + \Gamma_0)/2$. For finite $g_\kappa > 0$ these degeneracies are lifted and neither the frequencies nor the linewidths coincide for any g_ω. Instead, both quantities show a linear splitting for large g_ω, which naturally leads to a critical g_ω, above which $\kappa_- < 0$, i.e., to an instability. When this point is approached from below, one of the two normal modes becomes a parametric amplifier, as can also be seen in panels **c** and **d**, where S_{11} of the HF mode is plotted for eleven different g_ω between $2\pi \times 50$ Hz and $2\pi \times 50$ MHz for the case $g_\kappa/g_\omega = -2$. The corresponding points are also marked in **a** and **b** with circles. In **c** we show the case of $\kappa_\mathrm{ext} = 0.07\kappa_0$, which corresponds approximately to our current device, and in **d** we use $\kappa_\mathrm{ext} = 0.99\kappa_0$, as one would choose for a dedicated parametric amplifier; $\kappa_0 = 2\pi \times 100$ MHz.

where $\delta = \omega_\mathrm{p} - (\omega_0 - \Omega_0)$.

The two solutions are

$$\tilde{\omega}_\pm = \omega_0 + \frac{\delta}{2} + \mathrm{i}\frac{\kappa_0 + \Gamma_0}{4} \pm \sqrt{g_\omega^2 + \mathrm{i}\frac{g_\omega g_\kappa}{2} - \left(\frac{\kappa_0 - \Gamma_0 + 2\mathrm{i}\delta}{4}\right)^2} \tag{S237}$$

which differ from the dispersive normal-mode-splitting only via the term $\mathrm{i}g_\omega g_\kappa/2$ in the square-root. The actual resonance frequencies of the two normal modes are then given by $\omega_\pm = \mathrm{Re}(\tilde{\omega}_\pm)$ and the linewidths by $\kappa_\pm = 2\mathrm{Im}(\tilde{\omega}_\pm)$.

The new term in the square root has considerable consequences, and we will discuss them for the most common case $\delta = 0$. The additional term leads to the two normal modes splitting in resonance frequency for any $g_\omega > 0$ and never snapping to the same linewidth as would be the case for $g_\kappa = 0$. Furthermore, the linewidth of one normal mode will decrease again after an initial increase until it becomes negative for sufficiently large g_ω. The two solutions of Eq. (S237) in the case $\delta = 0$ are shown in Supplementary Fig. 13 for the usual four different $g_\kappa/g_\omega = (0, -2, -4, -6)$, showing the just described signatures. Note that it is always possible to find a detuning $\delta = 2g_\omega g_\kappa/(\kappa_0 - \Gamma_0)$ for any $g_\omega g_\kappa/2$, such that the imaginary parts in the square root cancel and $\kappa_+ = \kappa_-$. This however, is still different from the dispersive-only case for $\delta = 0$ and the two normal-modes will not be equal in e.g. $|S_{11}|$ as would be the case for $g_\omega = 0$; they will furthermore not split linearly in frequency for large g_ω.

We also plot S_{11} of the HF mode for various pump strengths in the case $g_\kappa/g_\omega = -2$. For comparison, we show the reflection for two different cases $\kappa_\mathrm{ext} = 2\pi \times 7\,\mathrm{MHz}$, i.e., our strongly undercoupled experimental device, but also for $\kappa_\mathrm{ext} = 2\pi \times 99\,\mathrm{MHz}$, i.e., a hypothetical overcoupled device. All other parameters are identical to the subsections 6 A and 6 B. For both cases, one of the two normal modes becomes an amplifier, when the coupling is increased, a direct consequence of the decreasing decay rate κ_- approaching an instability point. It will be interesting to test this prediction in future devices and to investigate the noise properties of such an amplifier, especially since it could be implemented with mechanical oscillators, i.e., Josephson-free and therefore with a large dynamic range, and with a larger bandwidth than many other optomechanical amplifiers.

D. Impact of an additional external-dissipative coupling

As a last subsection here, we briefly discuss the expected modifications in case the external-dissipative coupling were not negligible. For simplicity, we restrict ourselves again to the sideband-resolved regime with a highly undercoupled HF mode, since this is the relevant case for our experiment. Also, we will only consider the case of a pump tone around the red HF mode sideband and omit all HF mode nonlinearities.

The derivation of the equations of motion with both $g_{0\kappa_\mathrm{int}}$ and $g_{0\kappa_\mathrm{ext}}$ would be completely analogous to the one presented in Supplementary Note 5. The only difference in Eqs. (S130) and (S131) would be that each term containing $g_{0\kappa}$ will show up twice, once with $g_{0\kappa_\mathrm{int}}$ and once with $g_{0\kappa_\mathrm{ext}}$. Correspondingly, the input terms for the $g_{0\kappa_\mathrm{ext}}$ terms will be $\hat{a}_\mathrm{ext,in}$ instead of $\hat{a}_\mathrm{int,in}$. The main difference is, that these new input terms cannot be dropped in the linearization, since $\hat{a}_\mathrm{ext,in}$ contains the high-amplitude coherent pump tone and not just noise. Hence, after linearization and dropping irrelevant terms, we get in the sideband-resolved limit

$$\dot{\hat{c}} = \left(-\mathrm{i}\Delta_\mathrm{p} - \frac{\kappa_0}{2}\right)\hat{c} - \mathrm{i}\left(g_\omega + \mathrm{i}\frac{g_\kappa}{2}\right)\hat{d} - \frac{\mathrm{i}g_{0\kappa_\mathrm{ext}}\alpha_\mathrm{in}e^{\mathrm{i}\phi_\mathrm{p}}}{2\sqrt{\kappa_\mathrm{ext}}}\hat{d} + \mathrm{i}\sqrt{\kappa_\mathrm{ext}}\hat{c}_\mathrm{in} \tag{S238}$$

$$\dot{\hat{d}} = \left(\mathrm{i}\Omega_0 - \frac{\Gamma_0}{2}\right)\hat{d} - \mathrm{i}g_\omega\hat{c} - \frac{\mathrm{i}g_{0\kappa_\mathrm{ext}}\alpha_\mathrm{in}e^{-\mathrm{i}\phi_\mathrm{p}}}{2\sqrt{\kappa_\mathrm{ext}}}\hat{c} + \mathrm{i}\sqrt{\Gamma_\mathrm{ext}}\hat{d}_\mathrm{in} - \mathrm{i}\frac{g_{\kappa_\mathrm{ext}}}{2\sqrt{\kappa_\mathrm{ext}}}\hat{c}_\mathrm{in} \tag{S239}$$

with the total dissipative multi-photon coupling rate

$$g_\kappa = g_{\kappa_\mathrm{int}} + g_{\kappa_\mathrm{ext}} \tag{S240}$$

The two new terms with α_in originate from a direct coupling between the pump tone on the HF mode feedline and the LF circuit. The third new term, the last one in the EOM for the LF mode, describes a direct excitation of the LF mode by the beating between the intracavity pump field and the probe tone on the feedline. We can obtain the relation between α_0 and α_in in good approximation for the bare cavity as

$$\alpha_0 = \frac{\mathrm{i}\sqrt{\kappa_\mathrm{ext}}}{\frac{\kappa_0}{2} + \mathrm{i}\Delta_\mathrm{p}}\alpha_\mathrm{in}e^{\mathrm{i}\phi_\mathrm{p}} \tag{S241}$$

or in the sideband-resolved limit and with red-sideband pumping $|\Delta_\mathrm{p}| \gg \kappa_0/2$

$$\alpha_\mathrm{in} \approx \frac{\Delta_\mathrm{p}}{\sqrt{\kappa_\mathrm{ext}}}\alpha_0, \qquad \phi_\mathrm{p} \approx 0. \tag{S242}$$

Plugging this result and its complex conjugate into the equations of motion leads to

$$\dot{\hat{c}} = \left(-\mathrm{i}\Delta_\mathrm{p} - \frac{\kappa_0}{2}\right)\hat{c} - \mathrm{i}\left(g_\omega + \mathrm{i}\frac{g_\kappa}{2}\right)\hat{d} - \mathrm{i}g_{\kappa_\mathrm{ext}}\frac{\Delta_\mathrm{p}}{2\kappa_\mathrm{ext}}\hat{d} + \mathrm{i}\sqrt{\kappa_\mathrm{ext}}\hat{c}_\mathrm{in} \tag{S243}$$

$$\dot{\hat{d}} = \left(\mathrm{i}\Omega_0 - \frac{\Gamma_0}{2}\right)\hat{d} - \mathrm{i}g_\omega\hat{c} - \mathrm{i}g_{\kappa_\mathrm{ext}}\frac{\Delta_\mathrm{p}}{2\kappa_\mathrm{ext}}\hat{c} + \mathrm{i}\sqrt{\Gamma_\mathrm{ext}}\hat{d}_\mathrm{in} - \mathrm{i}\frac{g_{\kappa_\mathrm{ext}}}{2\sqrt{\kappa_\mathrm{ext}}}\hat{c}_\mathrm{in} \tag{S244}$$

and after defining the effective dispersive coupling rate

$$\overline{g}_\omega = g_\omega + g_{\kappa_\mathrm{ext}}\frac{\Delta_\mathrm{p}}{2\kappa_\mathrm{ext}} \tag{S245}$$

the EOMS read

$$\dot{\hat{c}} = \left(-\mathrm{i}\Delta_\mathrm{p} - \frac{\kappa_0}{2}\right)\hat{c} - \mathrm{i}\left(\overline{g}_\omega + \mathrm{i}\frac{g_\kappa}{2}\right)\hat{d} + \mathrm{i}\sqrt{\kappa_\mathrm{ext}}\hat{c}_\mathrm{in} \tag{S246}$$

$$\dot{\hat{d}} = \left(\mathrm{i}\Omega_0 - \frac{\Gamma_0}{2}\right)\hat{d} - \mathrm{i}\overline{g}_\omega\hat{c} + \mathrm{i}\sqrt{\Gamma_\mathrm{ext}}\hat{d}_\mathrm{in} - \mathrm{i}\frac{g_{\kappa_\mathrm{ext}}}{2\sqrt{\kappa_\mathrm{ext}}}\hat{c}_\mathrm{in}. \tag{S247}$$

Hence, aside from the additional input term in the LF EOM $\propto\hat{c}_\mathrm{in}$, we recover the equations of motion without external-dissipative coupling, just with a re-scaled and explicitly detuning-dependent dispersive coupling rate $\overline{g}_\omega$. If the signs of Δ_p and $g_{0\kappa_\mathrm{ext}}$ are identical, the effective dispersive coupling rate $\overline{g}_\omega$ is enhanced compared to g_ω, if the signs are opposite $\overline{g}_\omega$ is reduced. Note that this result is in agreement with Ref. [10], and also explains why the external-dissipative coupling there did not lead to significant Fano interference in the transparency data, since there $g_{\kappa_\mathrm{int}} = 0$ and $g_{\kappa_\mathrm{ext}}/g_\omega \sim 0.01$, i.e., g_κ in Eq. (S246) can be neglected. In other words, the only relevant coupling term was the detuning-enhanced $\overline{g}_\omega$.

For the calculation of dynamical backaction we can set $\hat{c}_\mathrm{in} = 0$, and we get an identical result to vanishing external coupling, just with $\overline{g}_\omega$ instead of g_ω. For PPIT the situation is slightly more complicated, since have not only the additional probe-tone term in the LF EOM, but also a modified input-output relation at the probe tone frequency

$$\hat{c}_\mathrm{out} = \hat{c}_\mathrm{in} + \mathrm{i}\sqrt{\kappa_\mathrm{ext}}\hat{c} - \mathrm{i}\frac{g_{\kappa_\mathrm{ext}}}{2\sqrt{\kappa_\mathrm{ext}}}\hat{d}. \tag{S248}$$

Solving the EOMs by Fourier transformation, using $\overline{g} = \overline{g}_\omega + \mathrm{i}g_\kappa/2$ as well as $\hat{d}_\mathrm{in} = 0$, and combining the equations leads to

$$\hat{c} = \mathrm{i}\sqrt{\kappa_\mathrm{ext}}\chi_\mathrm{c}^\mathrm{eff}\left[1 + \mathrm{i}\frac{\overline{g}g_{\kappa_\mathrm{ext}}}{2\kappa_\mathrm{ext}}\chi_0\right]\hat{c}_\mathrm{in} \tag{S249}$$

$$\hat{d} = \sqrt{\kappa_\mathrm{ext}}\chi_0^\mathrm{eff}\left[\overline{g}_\omega\chi_\mathrm{c} - \mathrm{i}\frac{g_{\kappa_\mathrm{ext}}}{2\kappa_\mathrm{ext}}\right]\hat{c}_\mathrm{in} \tag{S250}$$

with

$$\chi_\mathrm{c}^\mathrm{eff} = \frac{\chi_\mathrm{c}}{1 + \overline{g}_\omega\overline{g}\chi_0\chi_\mathrm{c}}, \qquad \chi_0^\mathrm{eff} = \frac{\chi_0}{1 + \overline{g}_\omega\overline{g}\chi_0\chi_\mathrm{c}}. \tag{S251}$$

Finally, we can combine everything to find

$$S_{11} = \frac{\langle\hat{c}_\mathrm{out}\rangle}{\langle\hat{c}_\mathrm{in}\rangle} \tag{S252}$$

$$= 1 - \frac{g_{\kappa_\mathrm{ext}}^2}{2\kappa_\mathrm{ext}}\chi_0^\mathrm{eff} - \kappa_\mathrm{ext}\chi_\mathrm{c}^\mathrm{eff}\left[1 + \mathrm{i}\frac{\left(\overline{g}_\omega + \overline{g}\right)g_{\kappa_\mathrm{ext}}}{2\kappa_\mathrm{ext}}\chi_0\right]. \tag{S253}$$

For parameters like in our experiment, we can approximate this further, if we reasonably assume that $|g_{0\kappa_\mathrm{ext}}|/2\pi < 0.55\,\mathrm{kHz}$, cf. Supplementary Note 3. This would correspond to $|g_{\kappa_\mathrm{ext}}|/2\pi < 30\,\mathrm{kHz}$ at the highest experimental pump photon numbers of $\sim$2500, and for the second term in the reflection equation we would find $g_{\kappa_\mathrm{ext}}^2|\chi_0^\mathrm{eff}|/2\kappa_\mathrm{ext} < 10^{-3}$. At least for the lower pump photon number range, also the second term in the square brackets can be neglected, which is $\lesssim 0.05 + 0.04\mathrm{i}$ for $n_\mathrm{c} \sim 2500$ (calculated at $\Omega = \Omega_0$) and is linear in n_c. Hence, in this approximation, we get

$$S_{11} = 1 - \kappa_\mathrm{ext}\chi_\mathrm{c}^\mathrm{eff} \tag{S254}$$

which is formally identical to the case $g_{\kappa_\mathrm{ext}} = 0$.

It is not the same though as for $g_{\kappa_{\mathrm{ext}}} = 0$, the coupling rate $\overline{g}_{\omega}$ inside of $\chi_{\mathrm{c}}^{\mathrm{eff}}$ scales differently as a function of pump detuning compared to g_{ω}, and so would the dynamical backaction, which even has a contribution $\propto\overline{g}_{\omega}^{2}$, cf. Eq. (S169). The difference would be expected to be maximal for the largest detunings between pump and HF mode and the dispersive coupling rates g_{ω} (we omit the primes here to avoid confusion and remain consistent within this section) we extracted from the PPIT data would not fall on top of a single line as in main paper Fig. 4**c**, but spread out due to the detuning-dependent scaling. Within each power (color in main paper Fig. 4), the point of the lowest photon number would be pushed to higher g_{ω}, while the points for the largest photon numbers would be pulled to smaller g_{ω} compared to the fit line through all of them, in case $g_{0\kappa_{\mathrm{ext}}} < 0$. For $g_{\kappa_{\mathrm{ext}}} > 0$ the trends would be opposite. Furthermore, for the lowest photon numbers and largest detunings, i.e., the leftmost points in main paper Fig. 4**c**, $\overline{g}_{\omega}$ would be more than 1.5 times as large as g_{ω} if $g_{0\kappa_{\mathrm{ext}}}/2\pi \sim -0.55\,\mathrm{kHz}$ as suggested by the fit line in Supplementary Fig. 6. The dissipative coupling rate g_{κ} would be essentially unmodified though, and hence the experimentally determined ratio g_{κ}/g_{ω} would not approach the value obtained from the flux arcs.

Since we observe none of these consequences, we believe that our conclusions – the flux tuning of κ_{ext} is an analysis artifact and the external-dissipative coupling rate is truly negligible – are well justified. It will be very interesting to design and investigate such an external-dissipative coupling in the future though, also beyond the sideband-resolved limit and with adjustable sign, since there seems rich physics waiting to be explored.

7. SUPPLEMENTARY NOTE 7: DATA ANALYSIS AND FITTING ROUTINES

A. Background correction and basic fitting routine

In an ideal world, the reflection data of LF mode, HF cavity and PPIT experiment would be described by the response functions Eqs. (S45, S73, S182), respectively. However, the actual signal is considerably modified by various accumulating effects of the experimental setup and multiple system components. The cables have a finite length and a frequency-dependent attenuation, we have attenuators, directional couplers, amplifiers, dc blocks, wirebonds, a PCB and lots of SMA connectors mounted into the signal path, which all combined lead to attenuation, cable resonances, impedance mismatches and parasitic reflections or imperfect isolation, cf. e.g. Refs. [2, 3]. As a consequence, the actual reflection is approximately described by

$$S_{11}^{\mathrm{real}} = A(\omega)\left[1 - f(\omega)e^{i\theta(\omega)}\right]e^{i\alpha(\omega)}, \tag{S255}$$

where the ideal-world response would be

$$S_{11}^{\mathrm{ideal}} = 1 - f(\omega) \tag{S256}$$

and $f(\omega)$ is defined by the experiment performed. Here $A(\omega)$ is a real-valued and frequency-dependent attenuation/gain of the signal, $\alpha(\omega)$ is a frequency-dependent and real-valued phase accumulated by the signal on the path and $\theta(\omega)$ is a potentially frequency-dependent Fano-angle originating from impedance mismatches and/or interference with parasitic signal paths. If the cavity has a high Q, i.e., its resonance is spread over a small frequency range $\sim 2\kappa_0$, it is usually sufficient to approximate $A(\omega)$ as a second-order polynomial, and α as a linear function of ω. Here, this is true for the direct measurement of the LF mode, but not for the HF cavity, and so we need a more elaborate approach to handle HF data. The HF cavity has a linewidth of $\kappa_0 \sim 2\pi \times 100\,\mathrm{MHz}$ and the background over such a large span can have a much more complicated shape than a second-order polynomial, cf. Supplementary Fig. 14**a**. To compensate for this, we perform a multi-step background correction for the HF cavity and the PPIT zoom-ins, which is illustrated step-by-step in Supplementary Fig. 14.

To facilitate our approach, we take a high-power VNA trace $S_{11}^{\mathrm{bg,exp}}$ in addition to each low-power VNA dataset S_{11}^{exp} during the experiments, and the two are completely identical with respect to frequency points. We choose the VNA power of the high-power trace sufficiently large, that the cavity absorption dip has vanished due to its nonlinear damping and its Kerr frequency shift (we verify the vanished cavity by adding even more power and check if the transmission is still changing). In other words, $f(\omega) = 0$ and we measure the background reflection

$$S_{11}^{\mathrm{bg,exp}} = \tilde{A}(\omega)e^{i\tilde{\alpha}(\omega)}. \tag{S257}$$

We label the (new) functions $\tilde{A}$, $\tilde{\alpha}$ with a tilde to indicate that they might be slightly different from the ones in the low-power regime, in our case for instance due to a slight cryo-HEMT compression. The two traces, low-power and high-power, respectively, are shown for one of our datasets in Supplementary Fig. 14**a-c** in amplitude, phase and complex representation. The gray line is the high-power trace and the purple symbols are the low-power trace with the cavity resonance.

For the PPIT zooms, we go a slightly different way to have fully consistent backgrounds for wideband and narrowband data. We take the high-power dataset of the HF cavity and select from it the corresponding narrow frequency range measured in each PPIT zoom. Since this narrow frequency window of the HF dataset has a much smaller frequency resolution than the PPIT dataset, we fit the HF background in this span with a polynomial for both the amplitude and the phase. This works very

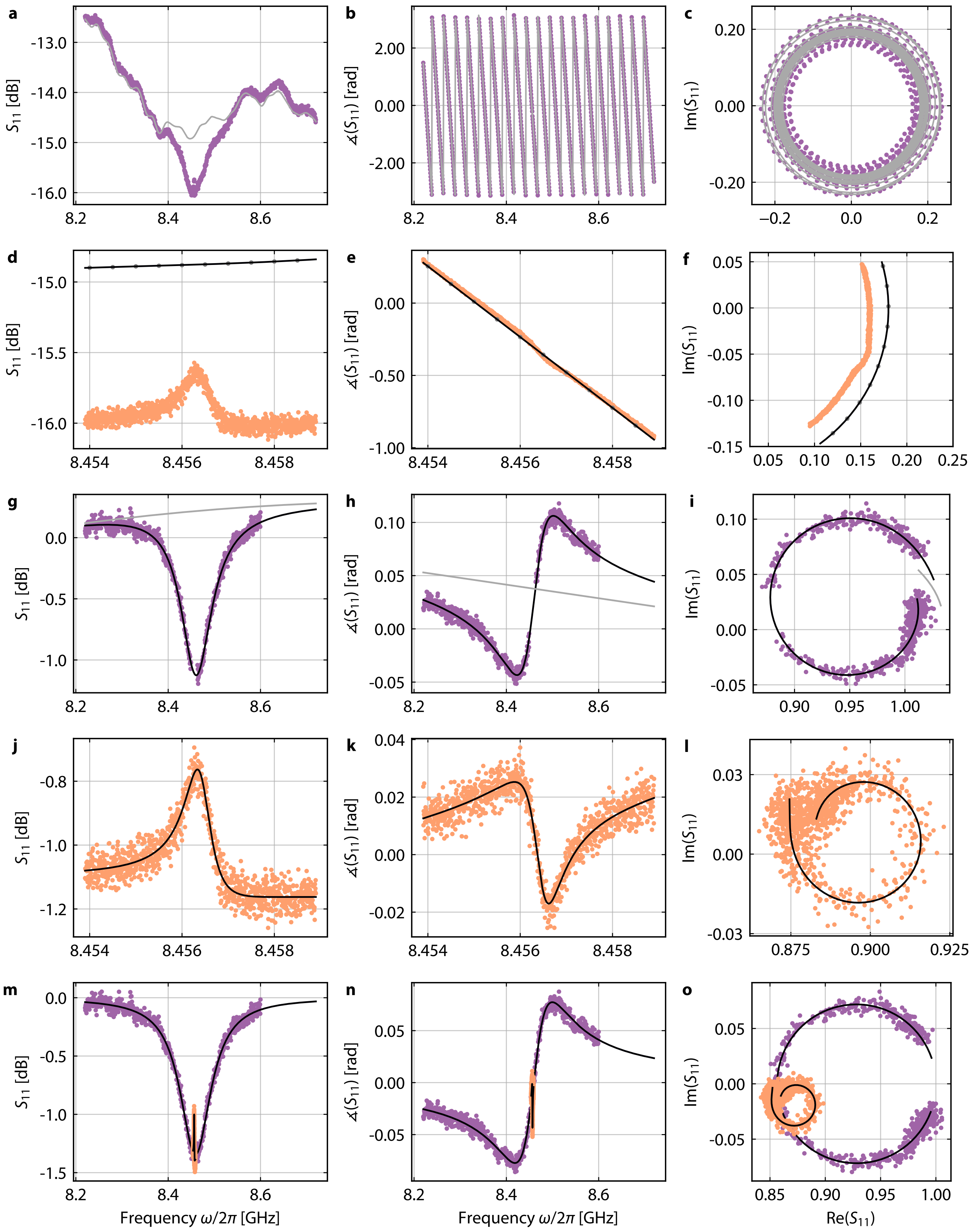

Supplementary Figure 14. **Multi-step background correction procedure.** Details, including a description of all panels **a**-**o**, are given in the text. Each panel row shows reflection data in amplitude (**a**, **d**, **g**, **j**, **m**), phase (**b**, **e**, **h**, **k**, **n**) and in the complex plane (**c**, **f**, **i**, **l**, **o**).

well, since the background does not vary much in such small frequency windows. With the obtained fit parameters, we can calculate the background for all the experimental PPIT frequency points. One example for a measured PPIT zoom with both its corresponding HF-snipped experimental background and its fit are shown in panels **d**-**f**. Circle symbols are HF background frequency points, gray line is the fit for the HF background, and orange symbols are the low-power PPIT data.

Using the two datasets for each VNA scan (measured data and measured or fitted background, respectively), we perform as a next step a complex division and get as result

$$S_{11}^{\mathrm{cor}} = \frac{S_{11}^{\mathrm{exp}}}{S_{11}^{\mathrm{bg,exp}}} \tag{S258}$$

$$= \frac{A(\omega)}{\tilde{A}(\omega)} \left[1 - f(\omega)\mathrm{e}^{\mathrm{i}\theta(\omega)}\right] \mathrm{e}^{\mathrm{i}[\alpha(\omega)-\tilde{\alpha}(\omega)]} \tag{S259}$$

$$= B(\omega) \left[1 - f(\omega)\mathrm{e}^{\mathrm{i}\theta(\omega)}\right] \mathrm{e}^{\mathrm{i}\phi(\omega)} \tag{S260}$$

where we usually get a very weak frequency dependence of the remaining parameters $B(\omega) \approx 1$ and $\phi(\omega) \approx 0$ for the cavity, cf. panels **g**-**i**, which show S_{11}^{cor} in amplitude, phase and complex representation of the original data in **a**-**c**. The corresponding first-step corrected PPIT data are shown in panels **j**-**l**.

In principle, we can now fit the cavity dataset to obtain $B(\omega)$, $\phi(\omega)$ and $\theta(\omega)$, but in practice we need to set $\theta(\omega) = \theta$ to be frequency-independent for our fit routine to run properly. Furthermore, we remove the data points, for which $\omega > 2\pi \times 8.6\,\mathrm{GHz}$, since there seems to be another small resonance, which modifies the cavity fits. For the first cavity fit, we remove the PPIT window from the cavity dataset, i.e., we remove all data points within a span of $15\Gamma_{\mathrm{eff}}$ around Ω_{eff} from the cavity scan. Then, we fit the remaining datapoints with

$$S_{11}^{\mathrm{cor,fit}} = B(\omega) \left[1 - f(\omega)\mathrm{e}^{\mathrm{i}\theta}\right] \mathrm{e}^{\mathrm{i}\phi(\omega)}, \tag{S261}$$

where

$$B(\omega) = b_0 + b_1\omega + b_2\omega^2 \tag{S262}$$

$$\phi(\omega) = \phi_0 + \phi_1\omega \tag{S263}$$

$$f(\omega) = \frac{\kappa_{\mathrm{ext}}}{\frac{\kappa_0}{2} + \mathrm{i}(\omega - \omega_0)} \tag{S264}$$

and get b_0, b_1, b_2, ϕ_0, ϕ_1 and θ as fit parameters. Simultaneously, we get first values for the cavity parameters ω_0, κ_{ext} and κ_0 (or ω_0', κ_0', and κ_{ext}', but we do not explicitly mention the primed quantities here).

We perform this procedure for all sideband pump powers and detunings. During this procedure, we find a small and nearly constant $\theta = 0.08$ for most datasets, only for the highest pump powers and largest PPIT signatures, the values deviate considerably from that, which we attribute to the large PPIT signature modifying the shape of the cavity even beyond the cutout window of $15\Gamma_{\mathrm{eff}}$. To deal with this, we re-run the fitting routine with Eqs. (S261)-(S264), but keep $\theta = \theta_0 = 0.08$ fixed for all fits this time. As a result, we have the updated background $S_{11}^{\mathrm{bg,fit}} = (b_0 + b_1\omega + b_2\omega^2)\mathrm{e}^{\mathrm{i}(\phi_0+\phi_1\omega)}$ as well as the updated cavity parameters ω_0, κ_{ext} and κ_0 from this second fit round. Then, we divide all experimentally corrected datasets by this fit background and rotate off the θ_0 angle

$$S_{11}^{\mathrm{final}} = \left(\frac{S_{11}^{\mathrm{cor}}}{S_{11}^{\mathrm{bg,fit}}} - 1\right)\mathrm{e}^{-\mathrm{i}\theta_0} + 1. \tag{S265}$$

We do this for all HF datasets equally, wideband or narrowband alike, and the result of this multi-step correction $S_{11} = S_{11}^{\mathrm{final}}$ is what is plotted as HF resonance or PPIT resonance in all figures of the main paper and Supplementary Material, except for the resonance before cJJ cutting in Supplementary Fig. 3. The fully adjusted and rotated data and fits are also included for the exemplary dataset of Supplementary Fig. 14 as panels **m**-**o**, wideband and narrowband data are overlaid in the graph to also show that they form one consistent dataset.

B. PPIT fitting routine

As described in the previous section, we first remove the PPIT signature from the cavity resonance data, and then fit the background-corrected cavity twice with Eqs. (S261)-(S264), in the first step with θ as fit parameter, in the second step with fixed θ_0 obtained from the θ analysis. From this routine, we obtain ω_0, κ_0 and κ_{ext} in the last step, and we keep those parameters fixed for all subsequent analysis steps. Note that we just generically use the unprimed variables in this and the previous subsection, in reality of course we obtain e.g. ω_0', κ_0' and κ_{ext}' in case of a sideband pump.

Similarly, we fit the PPIT zoom-ins with Eqs. (S261)-(S264) once and with θ being a fit parameter, and obtain from that fit $\Gamma_{\rm eff}$ and $\Omega_{\rm eff}$. All further fit parameters like the background contributions, the Fano-angle and $\Gamma_{\rm ext}$ are meaningless for the zoom fit and will be dropped, they just describe the small part of the cavity absorption dip, in which the PPIT signature is located for each pump frequency and power. This cavity-resonance-background we of course want to keep and thus we do not remove it from the PPIT datasets.

Then, we generate a combined single datafile from the completely background-corrected wideband and narrowband datasets and fit this combined dataset with the PPIT reflection Eq. (S226). The individual resonance parameters ω_0, κ_0, $\kappa_{\rm ext}$, $\Gamma_{\rm eff}$ and $\Omega_{\rm eff}$ are fixed from the previous fits and the remaining two unknowns g_ω and g_κ are obtained as fit parameters. The result of combining the data and the corresponding S_{11} PPIT-fits are used for main paper Fig. 3 and in Sec. 8 A.

Now, we have the complete multi-photon parameter set to describe the PPIT data $\omega_0, \kappa_0, \kappa_{\rm ext}, \Omega_{\rm eff}, \Gamma_{\rm eff}, g_\omega$ and g_κ. We limit our PPIT analysis to the pump detuning range $\delta \in [-\kappa_0'/4, +\kappa_0'/4]$, because for $\delta < \kappa_0'/4$ the transparency resonance gets very small and for $\delta > \kappa_0'/4$ we approach the dissipative red-sideband instability at high pump powers, cf. Sec. 6 A.

C. LF reflection fitting routine for dynamical backaction

The LF reflection datasets taken during the experiment for the characterization of dynamical backaction, cf. main paper Fig. 5**b** for one example, are first fitted using Eqs. (S261)-(S264), i.e., with a linear circuit response. Of course, we use different variables and quantities here, i.e. $\kappa_0 \to \Gamma_{\rm eff}$, $\kappa_{\rm ext} \to \Gamma_{\rm ext}$, $\omega \to \Omega$ and $\omega_0 \to \Omega_{\rm eff}$. In a next step, the background is divided off and a possible θ is rotated off; the background-corrected datasets are stored. Although the data were taken at a sufficiently low LF probe power that without dynamical backaction there are no signs of the intrinsic LF Kerr nonlineaerity in the response resonances, we found that close to the parametric instability, the resonances slightly and asymmetrically tilt to the left as typical for Duffing/Kerr nonlinearities. Hence, we re-fit the background-corrected resonances using Eqs. (S48)-(S50) with the replacements $\Gamma_0 \to \Gamma_{\rm eff}$, $\Omega_0 \to \Omega_{\rm eff}$ and $P_\beta = -97\,$dBm, which is our best estimate for the LF on-chip probe power. The fit parameters in this fit are $\Gamma_{\rm ext}$, $\Gamma_{\rm eff}$, $\Omega_{\rm eff}$ and $\mathcal{K}_{\rm LF}$, of which $\Gamma_{\rm eff}$ and $\Omega_{\rm eff}$ are used for plotting and further analyses, cf. main paper Fig. 5 and Supplementary Fig. 17. The data points, that are actually impacted by the slight LF self-Kerr tilt are roughly the last 10 points of each pump power before instability sets in; note also that mainly $\Omega_{\rm eff}$ is modified by this, but not $\Gamma_{\rm eff}$.

D. Photon number calibration

For much of our analysis, it is important to know the pump photon number $n_{\rm c}$, in particular for the analysis of the nonlinearity-enhanced coupling rates and the dynamical backaction evaluation. In principle, we could calculate it via Eqs. (S65) numerically, if we knew the on-chip pump power, i.e., the attenuation between the signal generator providing the pump tone and the chip, as well as the nonlinear coefficients $\mathcal{K}$, κ_1, κ_2 and κ_3. Unfortunately, we do not. This is obvious for the nonlinear coefficients, especially for the $\kappa_1, \kappa_2, \kappa_3$, since there is no theory available that could be used to estimate their values, and to extract their values from data, we would need to know $n_{\rm c}$ in the first place. Regarding the attenuation, it can in principle be calibrated with e.g. a temperature-controlled white noise source at the input of the HEMT, but since we do not have space for a microwave switch in the setup and measure directly in liquid helium (making accurate T-control impractical) this was also not a reasonable option. Furthermore, the attenuation is considerably frequency-dependent over the more than 300 MHz wide range of relevant $\omega_{\rm p}$ for main paper Fig. 5, which is supported by the frequency-dependence of the VNA probe tone reflection, cf. Supplementary Fig. 14**a**.

Therefore, we use a different approach to get $n_{\rm c}$, which only requires the value for $g_{0\omega}$, that we get from the HF flux arc derivative and the LF zero-point-fluctuations, and that we assume the only dispersive nonlinearity of the HF mode to be a Kerr anharmonicity. From the experiment with a sideband pump tone, we know that the increase of κ_0' with respect to κ_0 is sufficiently small compared to the other frequencies that we can apply the approximations of Sec. 4 H and use Eq. (S96) to calculate

$$\mathcal{K} n_{\rm c} = \frac{\delta\omega_0\left(2 - \delta\omega_0/\Delta_{\rm p}\right)}{4} \tag{S266}$$

where $\delta\omega_0 = \omega_0' - \omega_0$ is the pump-induced HF frequency shift, $\Delta_{\rm p} = \omega_{\rm p} - \omega_0$ is the detuning between pump tone and unpumped HF cavity and $\mathcal{K}$ is the Kerr anharmonicity.

As a next step, we normalize the total dispersive multiphoton coupling rate data g_ω' (obtained from the PPIT experiments) as

$$\frac{g_\omega'}{\sqrt{\mathcal{K} n_{\rm c}}} = g_{0\omega}\alpha_{\mathcal{K}} + g_{0\mathcal{K}}\mathcal{K} n_{\rm c}\alpha_{\mathcal{K}}^3 \tag{S267}$$

where $\alpha_{\mathcal{K}} = 1/\sqrt{\mathcal{K}}$, and then fit the normalized coupling rates as a function of $\mathcal{K} n_{\rm c}$ with $\alpha_{\mathcal{K}}$ and $g_{0\mathcal{K}}$ as the fit parameters and $g_{0\omega} = 2\pi \times 27.1\,$kHz. From this approach, we obtain $\mathcal{K} = 2\pi \times -5.4\,$kHz and subsequently $n_{\rm c}$ for all datasets. Note, that equivalently we could plug Eq. (S266) into the equation for g_ω' and then fit g_ω' as a function of $\delta\omega_0$ with $\mathcal{K}$ (and $g_{0\mathcal{K}}$) as fit

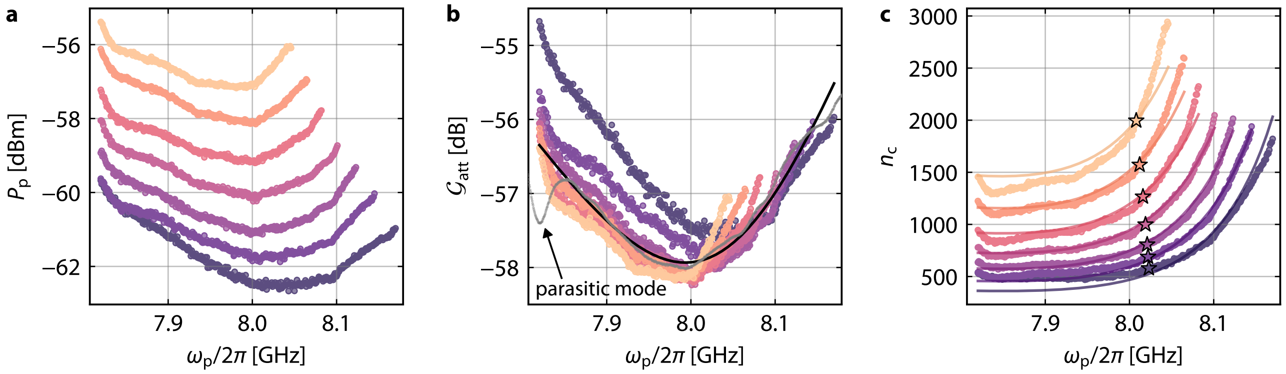

Supplementary Figure 15. **On-chip pump power, pump-line attenuation and intracavity photon number as a function of frequency.** **a** Inferred on-chip pump power as a function of pump frequency calculated with Eq. (S268), using the experimental HF mode parameters ω_0', κ_0' as well as their fits as a function of n_c. Different colors correspond to different signal generator powers between $P_{sg}^{min} = -5$ dBm and $P_{sg}^{max} = +1$ dBm in steps of 1 dB. Lowest P_p corresponds to lowest P_{sg}, highest P_p corresponds to highest P_{sg}. **b** Pump-line attenuation $\mathcal{G}_{att} = P_p/P_{sg}$ as a function of pump frequency ω_p and for seven different P_{sg}, color-code is analogous to **a**. Symbols are data, black line is a simultaneous fit of the six highest P_p datasets with a fourth-order polynomial, which we use as average pump attenuation $\overline{\mathcal{G}}_{att}$ for further processing. Small gray symbols show the VNA probe tone attenuation in that frequency window, shifted manually by −43.4 dB to get to the same level of magnitude; despite the much longer path of the probe tone, that includes also the way back from the sample to the VNA, the qualitative agreement in shape is considerable. It also shows a parasitic on-chip mode at ~7.82 GHz, which might have an impact to the power-dependence of $\mathcal{G}_{att}$. **c** Experimentally obtained (symbols) and numerically calculated (lines) pump photon n_c vs. pump frequency ω_p. For the calculated numbers, we use Eq. (S65) and the mean attenuation $\overline{\mathcal{G}}_{att}$ shown in **b** as fit line. Star symbols correspond to an effective pump-sideband detuning $\delta' = 0$, cf. main paper Fig. 5 and Supplementary Fig. 17.

parameter. This approach would give slightly different weights to the data points, but the result for $\mathcal{K}$ and n_c only differs by <5%.

To model the dynamical backaction in main paper Fig. 5, which is measured and presented as a function of pump detuning Δ_p', we furthermore need a function for $n_c(\Delta_p')$. Combined with the fit/theory functions for $g_\omega'(n_c)$, $g_\kappa'(n_c)$, $\omega_0'(n_c)$, and $\kappa_0'(n_c)$, which can be performed/calculated now that n_c is calibrated, we have everything we need to model $\Gamma_{eff}(\Delta_p')$ and $\Omega_{eff}(\Delta_p')$. The fit values for the nonlinear coefficients are $\kappa_1 = 2\pi \times 3.7$ kHz, $\kappa_2 \approx 0$, $\kappa_3 = 2\pi \times 0.37$ mHz, $g_{nl1} = 2\pi \times -22.1$ Hz, $g_{nl2} \approx 0$, $g_{nl3} = 2\pi \times -3.5$ µHz, and $g_{0\mathcal{K}} = 2\pi \times 10.0$ Hz. The procedure to obtain $n_c(\Delta_p')$ will be discussed in the next subsection.

E. On-chip pump power calibration

To calibrate the on-chip pump power, we need to find an estimate for the pump attenuation between signal generator and device. For this, we use Eq. (S97) and solve for the pump power

$$P_p = \frac{n_c \hbar \omega_p}{4} \frac{\kappa_{eff}^2 + 4\tilde{\Delta}_p^2}{\kappa_{ext}}. \tag{S268}$$

For each datapoint (ω_p, P_p), we take now the experimental values for n_c, ω_p and κ_{ext}, and we calculate

$$\kappa_{eff} = \kappa_0 + \kappa_1 n_c + \kappa_2 n_c^2 + \kappa_3 n_c^3 \tag{S269}$$

$$\tilde{\Delta}_p = \Delta_p - \mathcal{K} n_c \tag{S270}$$

using the experimental value for $\Delta_p = \omega_p - \omega_0$ and the fit values for $\mathcal{K}$, κ_1, κ_2 and κ_3.

The frequency-dependent attenuation $\mathcal{G}_{att}$ can then be calculated for each datapoint individually by

$$\mathcal{G}_{att} = \frac{P_p}{P_{sg}} \tag{S271}$$

with the signal generator output power P_{sg}. The result of this calculation is shown for all data points in Supplementary Fig. 15. Interestingly, we find not only a frequency-dependent attenuation, but it seems also to be a pump-power dependent attenuation. We believe this is not just an artifact, but at least partly the result of a second chip mode located at around 7.82 GHz. This second mode is much less nonlinear compared to the target HF mode, but since the pump is much closer to it, it will still absorb photons from the pump tone and if it shifts as a function of pump power, this absorption itself is nonlinear. Of course, other

factors might also contribute to a seemingly power-dependent attenuation, such as a nonlinear response of the HF feedline or the fingers in the interdigitated coupling capacitor, a power-dependent input impedance of the cryogenic HEMT amplifier, a power-dependent $\kappa_{\rm ext}$, higher-order dispersive nonlinearities in the HF mode, or deviations of the actual cavity parameters κ_0' and ω_0' from their fit values due to uncertainties in the background correction routine.

For the purpose of modeling the dynamical backaction, however, and to demonstrate that theory and experiment are consistent with each other, we believe it is most convincing to work with a single averaged attenuation. To have a general smooth function for $\mathcal{G}_{\rm att}$, we fit all the obtained data points combined with a fourth order polynomial

$$\overline{\mathcal{G}}_{\rm att}(\omega_{\rm p}) = \mathcal{G}_0 + \mathcal{G}_1\omega_{\rm p} + \mathcal{G}_2\omega_{\rm p}^2 + \mathcal{G}_3\omega_{\rm p}^3 + \mathcal{G}_4\omega_{\rm p}^4, \tag{S272}$$

the result is overlaid to the data points in Supplementary Fig. 15 and shows acceptable agreement. We neglect the lowest-power dataset for this fit, since it deviates strongly from the others for $\omega_{\rm p} \lesssim 2\pi \times 8\,$GHz. Whenever we give on-chip pump power values in the main manuscript, we calculate this value using the generator output power and $\overline{\mathcal{G}}_{\rm att}(\omega = 2\pi \times 8\,\text{GHz})$, and we round the power values to integers.

To finally calculate $n_{\rm c}(\omega_{\rm p})$, we numerically solve the polynomial Eq. (S65) with all the parameters as obtained from the fit of κ_0' and the analysis of ω_0', while using as input photon flux

$$n_{\rm in} = \overline{\mathcal{G}}_{\rm att}(\omega_{\rm p})\frac{P_{\rm sg}}{\hbar\omega_{\rm p}}. \tag{S273}$$

The result of this calculation in direct comparison with the values obtained from the experiment and $g_{0\omega}$ is shown in Supplementary Fig. 15**c**. In particular for the lower generator powers, the agreement is very good over the most important range of $\omega_{\rm p}$ between 7.9 GHz and 8.1 GHz. The remaining power-dependent deviations of up to ~15% originate mainly from the deviation between the individual $\mathcal{G}_{\rm att}$ and $\overline{\mathcal{G}}_{\rm att}$.

8. SUPPLEMENTARY NOTE 8: ADDITIONAL DATA

A. Resonant PPIT data for all pump powers

In Fig. 3 of the main paper, we present the PPIT data for $\delta_{\rm eff} \approx 0$ and an on-chip pump power of $P_{\rm p} = -58\,$dBm. In Supplementary Fig. 16 we show for completeness the datasets and fits for all pump powers between $P_{\rm p} = -63\,$dBm and $P_{\rm p} = -57\,$dBm. First, we notice that with increasing $P_{\rm p}$ the (internal) cavity linewidth increases as also discussed in main paper Fig. 4. Furthermore, we observe that the PPIT signature both in reflection magnitude and in the complex plane grows with $P_{\rm p}$ due to the increased coupling rates g_ω' and g_κ'. For all pump powers, the PPIT circle is considerably rotated away from the real axis. However, a graphical and quantitative γ-analysis analogous to main paper Fig. 3 is difficult here, especially for the low pump-powers, due to the small but still finite $\delta_{\rm eff}$ of all datasets (pump frequency step width was 2 MHz). A small offset between PPIT and cavity resonance is particularly noticeable for small PPIT circles, i.e., low pump powers, and using the actual reference point of the cavity circle ($-\delta_{\rm eff}$ away from resonance) is not very insightful, since then none of the resonances is on the real axis and the definition of γ requires two angled lines instead of one and the real axis. Therefore, we omit γ in the plot.

B. Dynamical backaction for all pump powers

Finally, we show the effective LF linewidth $\Gamma_{\rm eff}$ and effective LF resonance frequency $\Omega_{\rm eff}$, which include the dynamical backaction $\Gamma_{\rm pp}$ and $\delta\Omega_{\rm pp}$, for all pump powers in Supplementary Fig. 17. As described in the main paper in the context of Fig. 5, we obtain the experimental values for $\Gamma_{\rm eff}$ and $\Omega_{\rm eff}$ by probing directly the LF resonance with a VNA, while stepping the pump tone in steps of 2 MHz through the red sideband of the HF mode. The on-chip LF probe-tone power we estimate to be $P_\beta = -97\,$dBm, which at $\Omega = \Omega_0 = 2\pi \times 446\,$MHz, $\Gamma_0/2\pi = 600\,$kHz and $\Gamma_{\rm ext}/2\pi = 37\,$kHz corresponds to a intracircuit probe-photon number $n_{\rm pr} \sim 44000$. This is sufficiently low to stay below the onset of a LF self-Kerr nonlinearity or a modification of the dynamical backaction, except for the ~10 datasets closest to the parametric instability, where slight Duffing-like deformations of the LF resonances appear, cf. Supplementary Note 7 C for the corresponding fitting routine. Then, we model the data using

$$\Gamma_{\rm eff} = \Gamma_0 + \kappa_{\rm c} n_{\rm c} + \Gamma_{\rm pp} \tag{S274}$$

$$\Omega_{\rm eff} = \Omega_0 + \mathcal{K}_{\rm c} n_{\rm c} + \delta\Omega_{\rm pp} \tag{S275}$$

with the cross-Kerr constant $\mathcal{K}_{\rm c}$, the cross-nonlinear-damping $\kappa_{\rm c}$ and Ω_0 and Γ_0 as fit parameters. The photon-pressure dynamical backaction contributions $\Gamma_{\rm pp}$ and $\delta\Omega_{\rm pp}$ we calculate using the independently obtained functions for $g_\omega'(n_{\rm c})$, $g_\kappa'(n_{\rm c})$, $\omega_0'(n_{\rm c})$ and $\kappa_0'(n_{\rm c})$ in combination with $n_{\rm c}(\omega_{\rm p})$ as described in the main paper Fig. 4 and Sec. 7 of the Supplementary Material. The fit is performed with a single function for $\Gamma_{\rm eff}$ and $\delta\Omega_{\rm eff}$ and simultaneously for all powers, and the result agrees well with the experimental data. The remaining deviations originate from uncertainties in the pump attenuation and the intracavity pump photon number, respectively, and especially for high $n_{\rm c}$ from possible higher-order nonlinearities and the onset of parametric instability.

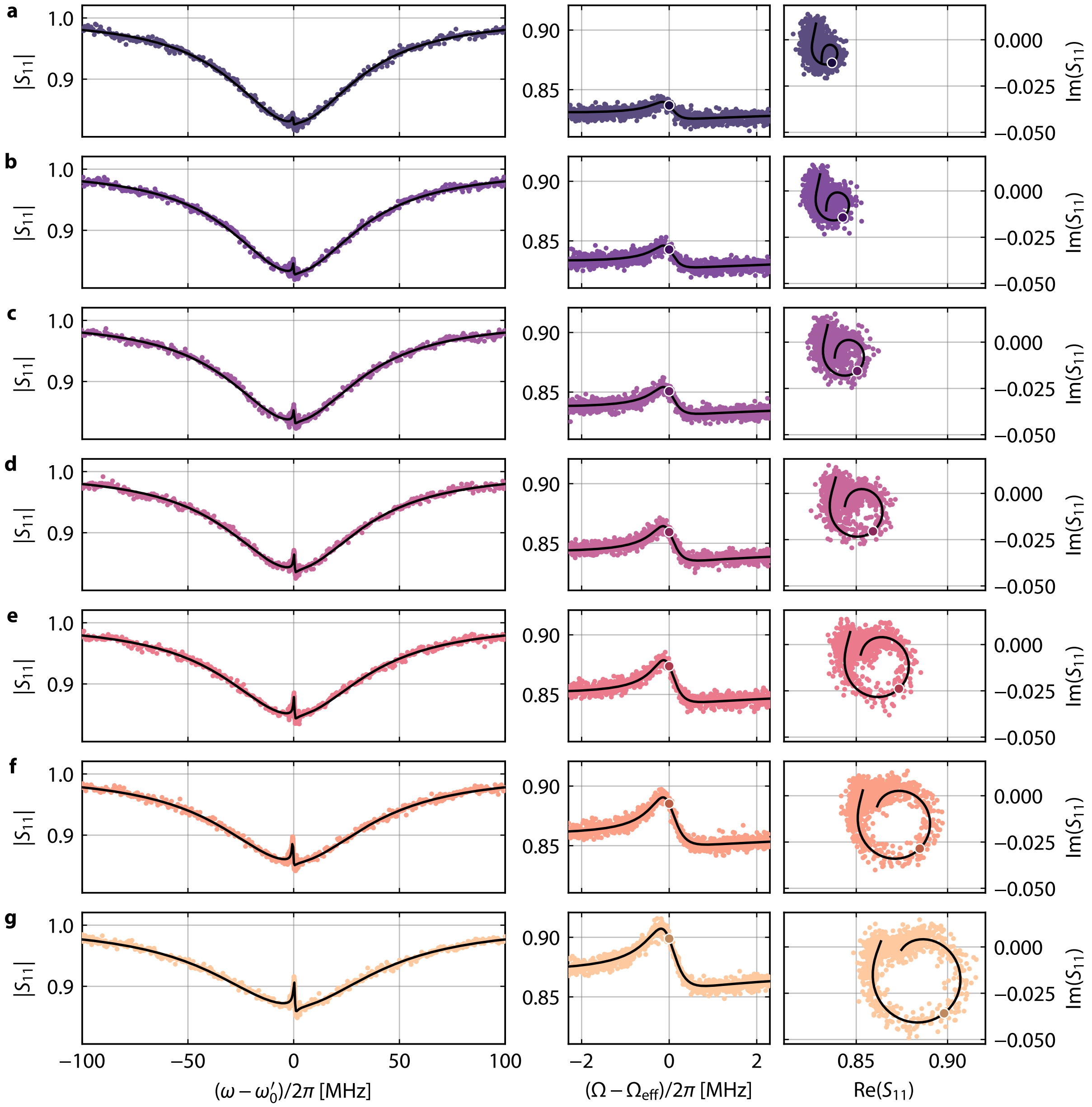

Supplementary Figure 16. **Near-resonant PPIT datasets for all investigated pump powers.** All panels show S_{11} of a photon-pressure-induced transparency experiment, from **a** to **g** the on-chip pump power increases from $P_\mathrm{p} = -63\,\mathrm{dBm}$ to $P_\mathrm{p} = -57\,\mathrm{dBm}$ in steps of 1 dB. Left column shows $|S_{11}|$ with a wide frequency span of 200 MHz, middle column shows corresponding zooms to the PPIT resonance with a narrow frequency span of 4.5 MHz, right column shows the zoom of S_{11} in the complex plane. Colored dots are experimental data, lines are fit curves, and colored disks with white boundaries mark the points of the PPIT resonances. With increasing P_p, the transparency window grows and becomes more asymmetric, indicating increasing coupling rates g_ω', g_κ' and a slightly increasing coupling rate ratio g_κ'/g_ω'. Since $\delta_\mathrm{eff} \approx 0$, but still $\neq 0$ for most of the datasets, the angle γ is omitted here.

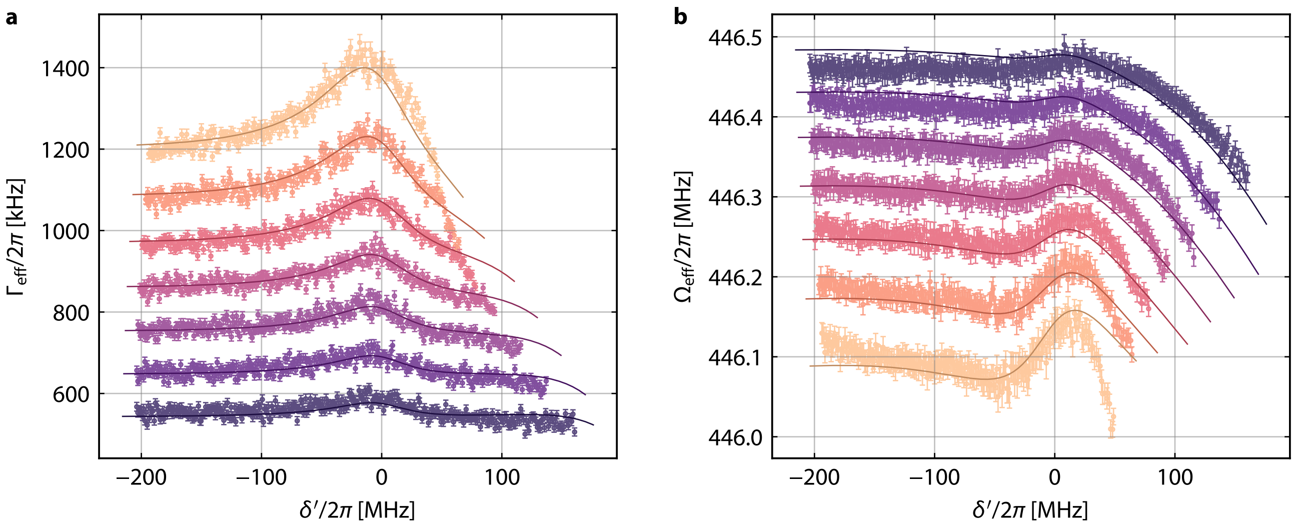

Supplementary Figure 17. **Dynamical backaction for all investigated pump powers.** **a** Effective LF mode linewidth $\Gamma_{\rm eff}$ and **b** effective LF mode resonance frequency $\Omega_{\rm eff}$ for all investigated pump powers, plotted vs. pump detuning from the red sideband $\delta' = \omega_{\rm p} - \left(\omega_0' - \Omega_0'\right)$. On-chip pump powers are between −63 dBm (dark blue) and −57 dBm (yellow) in steps of 1 dB (power values at 8 GHz). Data acquisition was identical to main paper Fig. 5, error bars show the standard error from the fit routine. Symbols are data points, solid lines are simultaneous fit curves of all $\Gamma_{\rm eff}$ and $\Omega_{\rm eff}$ with the free parameters $\Omega_0 = 2\pi \times 446.53$ MHz, $\Gamma_0 = 2\pi \times 527.0$ kHz, $\mathcal{K}_{\rm c} = 2\pi \times -130.8$ Hz, and $\kappa_{\rm c} = 2\pi \times 38.4$ Hz. Adjacent curves are manually shifted by +100 kHz each for $\Gamma_{\rm eff}$ and by −40 kHz each for $\Omega_{\rm eff}$ for higher visibility.

9. SUPPLEMENTARY REFERENCES

[1] Igreja, R. and Dias, C. J., Analytical evaluation of the interdigital electrodes capacitance for a multi-layered structure. Sensors and Actuators A **112**, 291-301 (2004)

[2] Wenner, J., Neeley, M., Bialczak, Radoslav C., Lenander, M., Lucero, E., O'Connell, A. D., Sank, D., Wang, H., Weides, M., Cleland, A. N., and Martinis, J. M., Wirebond crosstalk and cavity modes in large chip mounts for superconducting qubits. Superconductor Science and Technology **24**, 065001 (2011)

[3] Rieger, D., Günzler, S., Spiecker, M., Nambisan, A., Wernsdorfer, W., and Pop, I. M., Fano Interference in Microwave Resonator Measurements. Physical Review Applied **20**, 014059 (2023)

[4] Khapaev, M. M., Kidiyarova-Shevchenko, A. Yu., Magnelind, P., and Kupriyanov, M. Yu., 3D-MLSI: software package for inductance calculation in multilayer superconducting integrated circuits. IEEE Transactions on Applied Superconductivity **11**, 1090-1093 (2001)

[5] Rodrigues, I. C., Bothner, D., and Steele, G. A., Cooling photon-pressure circuits into the quantum regime. Science Advances **7**, eabg6653 (2021)

[6] Uhl, K., Hackenbeck, D., Füger, C., Kleiner, R., Koelle, D., and Bothner, D., A flux-tunable $YBa_2Cu_3O_7$ quantum interference microwave circuit. Applied Physics Letters **122**, 182603 (2023)

[7] Uhl, K., Hackenbeck, D., Peter, J., Kleiner, R., Koelle, D., and Bothner, D., Niobium quantum interference microwave circuits with monolithic three-dimensional nanobridge junctions. Physical Review Applied **21**, 024051 (2024)

[8] Uhl, K., Hackenbeck, D., Koelle, D., Kleiner, R., and Bothner, D., Extracting the current-phase relation of a monolithic three-dimensional nanoconstriction using a dc-current-tunable superconducting microwave cavity. Physical Review Applied **22**, 064052 (2024)

[9] Kazouini, M., Peter, J., Guo, Z. E., Wilde, B., Uhl, K., Koelle, D., Kleiner, R., and Bothner, D., Data and scripts for: Tunable and nonlinearity-enhanced dispersive-plus-dissipative coupling in photon-pressure circuits. DOI:10.5281/zenodo.18269705 (2026)

[10] Primo, A. G., Pinho, P. V., Benevides, R., Gröblacher, S., Wiederhecker, G. S., and Mayer Alegre, T. P., Dissipative optomechanics in high-frequency nanomechanical resonators. Nature Communications **14**, 5793 (2023)

[11] Gely, M. F., Sanz Mora, A., Yanai, S., van der Spek, R., Bothner, D., and Steele, G. A., Apparent nonlinear damping triggered by quantum fluctuations. Nature Communications **14**, 7566 (2023)

[12] Rodrigues, I. C., Steele, G. A., and Bothner, D., Parametrically enhanced interactions and nonreciprocal bath dynamics in a photon-pressure Kerr amplifier. Science Advances **8**, eabq1690 (2022)

[13] Rodrigues, I. C., Steele, G. A., and Bothner, D., Photon Pressure with an Effective Negative Mass Microwave Mode. Physical Review Letters **132**, 203603 (2024)

[14] Gardiner, C. W. and Collett, M. J., Input and output in damped quantum systems: Quantum stochastic differential equations and the master equation. Physical Review A **31**, 3761 (1985)

[15] Elste, F., Girvin, S. M., and Clerk, A. A., Quantum Noise Interference and Backaction Cooling in Cavity Nanomechanics. Physical Review Letters **102**, 207209 (2009)

[16] Xuereb, A., Schnabel, R., and Hammerer, K., Dissipative Optomechanics in a Michelson-Sagnac Interferometer. Physical Review Letters **107**, 213604 (2011)

[17] Weiss, T. and Nunnenkamp, A., Quantum limit of laser cooling in dispersively and dissipatively coupled optomechanical systems. Physical Review A **88**, 023850 (2013)

[18] Bothner, D., Rodrigues, I. C., and Steele, G. A., Four-wave-cooling to the single phonon level in Kerr optomechanics. Communications Physics **5**, 33 (2022)
